# HALA: A Hybrid Dual-Population Optimizer Integrating an Enhanced Artificial Lemming Algorithm and SHADE

**DOI:** 10.3390/biomimetics11070464

**Published:** 2026-07-02

**Authors:** Han Yang, Xingwang Huang

**Affiliations:** 1 School of Software, Xinjiang University, Urumqi 830091, China; 20232501223@stu.xju.edu.cn; 2 Computer Engineering College, Jimei University, Xiamen 361021, China

**Keywords:** dual-populationframework, artificial lemming algorithm (ALA), success-history-based adaptive differential evolution (SHADE), elite migration strategy, global optimization

## Abstract

The rapid development of intelligent systems has introduced increasingly sophisticated optimization problems across diverse domains. While contemporary metaheuristic algorithms, including the recent Artificial Lemming Algorithm (ALA), have shown considerable promise, they frequently encounter difficulties such as premature convergence, inadequate local refinement, and diminished performance in high-dimensional multimodal environments. To overcome these issues, this study presents HALA, a new hybrid dual-subpopulation optimizer that effectively integrates an enhanced ALA with the SHADE algorithm. HALA employs two interacting subpopulations: one leverages an improved ALA with hybrid *t*-distribution and Levy flight perturbations to promote persistent long-range exploration and diversity preservation; the other applies SHADE’s success-history adaptation and external archive for accurate local exploitation. Periodic bidirectional elite migration facilitates knowledge transfer between the subpopulations, reducing early stagnation in the enhanced ALA and strengthening SHADE’s global search capability. HALA is thoroughly benchmarked against 17 advanced metaheuristics, including ALA, LSHADE, LSHADE-SPACMA, AOOA, BAEO, BPBO, CCO, CEO, CQALA, DFL, DMOA, DHOA, FGO, KLA, PGA, SO, and SOO, using the IEEE CEC2017 suite in 10, 30, 50, and 100 dimensions and the IEEE CEC2022 suite in 10 dimensions. Comprehensive analyses involving qualitative visualization, convergence curves, boxplots, and statistical tests indicate that HALA achieves competitive or superior solution quality, comparable or faster convergence, and robust stability on a substantial proportion of the test instances. In particular, HALA obtains the most favorable Friedman average ranking values among the compared algorithms, which are 2.55, 2.38, 2.34, and 2.55 for the 10-, 30-, 50-, and 100-dimensional CEC2017 functions, respectively, and 2.58 for the 12 10-dimensional CEC2022 functions. Moreover, HALA is successfully applied to five well-known constrained engineering design problems—pressure vessel, rolling element bearing, tension/compression spring, cantilever beam, and gear train—where it reliably achieves optimal or near-optimal results that match or surpass the compared methods. These findings underscore HALA’s competitive strength and broad potential for practical engineering optimization.

## 1. Introduction

The rapid advancement of intelligent systems has given rise to increasingly complex optimization problems across diverse domains, including engineering design [1,2,3,4], finance [5], machine learning [6], and scientific computing [7,8,9,10]. These challenges are typically characterized by high dimensionality, nonlinearity, multimodality, and the presence of constraints, rendering traditional gradient-based methods ineffective due to their dependence on derivative information and vulnerability to local optima [11].

As a result, metaheuristic algorithms have gained widespread popularity as flexible, derivative-free stochastic optimization techniques that exhibit robustness in navigating complex search spaces [12]. Inspired by natural phenomena, evolutionary processes, swarm intelligence, physical laws, or human behavior, metaheuristics require minimal problem-specific knowledge and have been successfully applied to a broad range of real-world problems [13].

Despite significant progress, individual metaheuristic algorithms often suffer from inherent limitations, such as premature convergence, insufficient exploitation in later stages, rapid loss of population diversity, and poor scalability in high-dimensional multimodal landscapes [14]. The No Free Lunch theorem formally establishes that no single algorithm can universally outperform all others across every problem type, thereby motivating continuous innovation through hybridization and novel mechanism design [15]. Hybrid approaches, especially those employing dual- or multi-population structures, have demonstrated particular promise in achieving a superior balance between exploration and exploitation by combining complementary algorithmic strengths [16].

Recently, the Artificial Lemming Algorithm (ALA), a bio-inspired swarm intelligence method introduced in 2025, has shown impressive global exploration capabilities on standard benchmarks and engineering tasks [14]. However, like many swarm-based optimizers, ALA tends to converge prematurely and lacks precision in the exploitation phase due to rapid diversity loss in later iterations. Recent enhancements address these limitations. For instance, variants incorporate chaotic initialization and quasi-oppositional learning for improved diversity and convergence [17], or employ adaptive *t*-distribution perturbations, hybrid mutation strategies, and dynamic mechanisms to better balance exploration and exploitation [18,19]. Despite these advances, single-population ALA variants often exhibit stagnation in high-dimensional multimodal landscapes, with added mechanisms introducing computational overhead without fully mitigating late-stage exploitation weaknesses.

In contrast, Success-History-based Adaptive Differential Evolution (SHADE) excels at precise local refinement through success-history parameter adaptation and an external archive [20]. Unlike its extension L-SHADE, which employs linear population size reduction [21], the original SHADE maintains a fixed population size throughout the search process. This fixed-size design helps preserve diversity and simplifies integration within dual-population frameworks. However, SHADE exhibits relatively conservative global search behavior, making it susceptible to stagnation in highly multimodal landscapes.

To address these complementary weaknesses, this paper proposes the Hybrid Dual-Population Optimizer Integrating Enhanced ALA and SHADE (HALA). HALA synergistically combines an enhanced ALA subpopulation—with hybrid *t*-distribution and Levy flight perturbations to sustain heavy-tailed exploration and population diversity—and a SHADE subpopulation with fixed population size for superior local refinement. Periodic bidirectional elite migration facilitates information sharing between the two subpopulations, mitigating premature convergence in the enhanced ALA and enhancing SHADE’s capability to escape local optima.

The performance of HALA is rigorously evaluated against 17 state-of-the-art metaheuristics on the IEEE CEC2017 and CEC2022 benchmark suites across multiple dimensions, as well as on several constrained real-world engineering design problems. Experimental results demonstrate HALA’s superior accuracy, convergence speed, stability, and exploration–exploitation balance.

To position HALA within the broader landscape of metaheuristic optimization, Table 1 summarizes representative algorithms across five categories: ALA variants, SHADE/adaptive DE variants, cooperative co-evolution and multi-population frameworks, hybrid DE ensembles, and high-performing CEC competition algorithms. The table highlights that, while exploration-enhanced ALA variants and exploitation-enhanced SHADE variants have been studied extensively, the specific combination of an enhanced ALA with hybrid heavy-tailed perturbations and a fixed-size SHADE subpopulation, coupled with bidirectional elite migration, has not been previously investigated.

The main contributions of this work are:

- A dual-population hybrid framework that effectively combines enhanced ALA’s strong global exploration with SHADE’s precise local refinement.
- Introduction of hybrid *t*-distribution/Levy flight perturbations in the enhanced ALA subpopulation, coupled with bidirectional elite migration for sustained diversity and improved convergence.
- Comprehensive empirical validation establishing HALA’s top rankings on challenging benchmarks and competitive performance on practical engineering problems.

The remainder of this paper is organized as follows: Section 2 reviews the basic ALA and SHADE. Section 3 details the proposed HALA framework. Section 4 and Section 5 present experimental results on benchmarks and engineering applications, respectively. Section 6 discusses findings, and Section 7 concludes the paper with future directions.

## 2. Component Algorithms

In this section, the foundational algorithms of the proposed HALA are discussed briefly for convenience.

### 2.1. Artificial Lemming Algorithm (ALA)

The Artificial Lemming Algorithm (ALA), introduced in 2025, is a bio-inspired swarm intelligence optimizer that simulates four characteristic behaviors of lemmings: long-distance migration, burrow digging, foraging, and predator evasion [14]. A population of *N* individuals $X_{i}$ is initialized uniformly within the search bounds [lb,ub], and the global best position g is continuously tracked.

The core control mechanism is an energy factor that governs the transition between exploration and exploitation:

(1) $$E=2\log\left(1/\mathrm{rand}\right)\cdot \theta ,\;\theta =2\arctan\left(1-t/T_{\max}\right),$$

where *t* is the current iteration, $T_{\max}$ is the maximum number of iterations, and rand∈(0,1] is a uniform random number. The decay factor θ decreases smoothly from π to 0, producing a natural shift from high-energy exploration to low-energy exploitation.

In the high-energy phase (E>1):

Migration (probability 0.3): large-scale perturbations incorporating Brownian motion and guidance from both the global best g and random individuals:

(2) $$X_{i}^{\mathrm{new}}=g+F\cdot RB_{i}\cdot \left(r_{1}\cdot \left(g-X_{i}\right)+\left(1-r_{1}\right)\cdot \left(X_{i}-X_{r}\right)\right),$$

where $RB_{i}∼N{\left(0,1\right)}^{D}$ is a *D*-dimensional Brownian motion vector, F=±1 is a random direction flag, $r_{1}\in {\left[0,1\right]}^{D}$ is a dimension-wise weight vector, and $X_{r}$ (r≠i) is a randomly selected individual.

Digging: local neighborhood disturbances:

(3) $$X_{i}^{\mathrm{new}}=X_{i}+F\cdot r_{2}\cdot \left(g-X_{r}\right),\;r_{2}=\mathrm{rand}\cdot \left(1+\sin\left(0.5t\right)\right),$$

where $r_{2}$ introduces time-varying perturbation magnitude.

In the low-energy phase (E≤1):

Spiral foraging (probability 0.5): helical movements around the global best g to perform intensive local search:

(4) $$X_{i}^{\mathrm{new}}=g+F\cdot ∥g-X_{i}∥\cdot \left(\sin\left(2\pi r_{3}\right)+\cos\left(2\pi r_{3}\right)\right)\cdot \left(g-X_{i}\right),$$

where $r_{3}\in \left[0,1\right]$ is a uniform random number controlling the phase of the helical perturbation.

Predator evasion: Levy flight jumps with decaying step size to maintain occasional long-range exploration:

(5) $$X_{i}^{\mathrm{new}}=g+F\cdot G\cdot \mathrm{Levy}\left(D\right)\cdot \left(g-X_{i}\right),\;G=2\cdot \mathrm{sign}\left(\mathrm{rand}-0.5\right)\cdot \left(1-t/T_{\max}\right).$$

Here, *G* linearly decays from approximately 2 to 0, controlling the amplitude of Levy steps.

The complete pseudocode of the original ALA is presented in Algorithm 1.

ALA demonstrates excellent global exploration on multimodal benchmarks but suffers from rapid diversity loss due to strong attraction toward g, resulting in premature convergence and insufficient late-stage precision [14].
**Algorithm 1** Pseudocode of the Original ALA 1:  Initialize population *X* of size *N*, evaluate fitness, find global best g 2:  **for** t=1 to $T_{\max}$ **do** 3:   Compute $\theta =2\arctan(1-t/T_{\max})$ 4:   **for** each individual i=1 to *N* **do** 5:    Generate Brownian motion vector $RB_{i}∼N{\left(0,1\right)}^{D}$, direction flag F=±1 6:    Compute energy factor E=2log(1/rand)·θ 7:    **if** E>1 then▹ High-energy phase: exploration 8:     **if** rand<0.3 then▹ Migration 9:      $r_{1}=\mathrm{rand}\left(1,D\right)$10:     Select random individual index r≠i11:     $X_{i}^{\mathrm{new}}=g+F\cdot RB_{i}\cdot \left(r_{1}\cdot \left(g-X_{i}\right)+\left(1-r_{1}\right)\cdot \left(X_{i}-X_{r}\right)\right)$12:    else▹ Digging13:     $r_{2}=\mathrm{rand}\cdot \left(1+\sin\left(0.5\cdot t\right)\right)$14:     Select random individual index r≠i15:     $X_{i}^{\mathrm{new}}=X_{i}+F\cdot r_{2}\cdot \left(g-X_{r}\right)$16:    **end if**17:    else▹ Low-energy phase: exploitation18:    **if** rand<0.5 then▹ Spiral foraging19:     Compute distance $d_{i}=∥g-X_{i}∥$20:     $r_{3}=\mathrm{rand}$21:     $X_{i}^{\mathrm{new}}=g+F\cdot d_{i}\cdot \left(\sin\left(2\pi r_{3}\right)+\cos\left(2\pi r_{3}\right)\right)\cdot \left(g-X_{i}\right)$22:     else▹ Predator evasion23:     $G=2\cdot \mathrm{sign}\left(\mathrm{rand}-0.5\right)\cdot \left(1-t/T_{\max}\right)$24:     Generate Levy step Levy(D)25:     $X_{i}^{\mathrm{new}}=g+F\cdot G\cdot \mathrm{Levy}\left(D\right)\cdot \left(g-X_{i}\right)$26:    **end if**27:   **end if**28:   Bound $X_{i}^{\mathrm{new}}$ within [lb,ub], evaluate fitness, perform greedy selection29:   **if** new fitness better than current global best **then**30:    Update g31:   **end if**32:  **end for**33: **end for**

### 2.2. SHADE Algorithm

SHADE is an advanced adaptive variant of Differential Evolution that builds upon JADE [31] by introducing a success-history-based parameter adaptation mechanism using a fixed-size historical memory [20]. It maintains two memory vectors $M_{F}$ and $M_{CR}$ of size *H* (typically 5–10) to store the weighted means of successful scaling factors and crossover rates. Initially, all entries are set to 0.5.

For each individual *i*, a memory index *k* is randomly selected, and the control parameters are generated as:

(6) $$F_{i}=\mathrm{randc}\left(M_{F,k},0.1\right),\;CR_{i}=\left\{\begin{array}{cc} 0, & \mathrm{if}\;M_{CR,k}=-1,\\ \mathrm{randn}(M_{CR,k},0.1), & \mathrm{otherwise}, \end{array}\right.$$

where randc and randn denote Cauchy and normal distributions, respectively, and −1 is a terminal value indicating that no successful CR values have been recorded for this memory entry yet. Invalid values are truncated or regenerated to ensure validity.

Mutation employs the “current-to-pbest/1” strategy:

(7) $$v_{i}=x_{i}+F_{i}\left(x_{pbest}-x_{i}\right)+F_{i}\left(x_{r1}-x_{r}\right),$$

where $x_{pbest}$ is randomly selected from the top p·N individuals (p∈[0.05,0.2]), $x_{r1}\neq i$ is drawn from the current population, and $x_{r}$ is randomly chosen from the union of the current population and an external archive *A*. The archive stores parent solutions discarded during selection to promote diversity; when its size exceeds a predefined limit (typically proportional to *N*), random elements are removed.

Binomial crossover is then applied with rate $CR_{i}$ to produce the trial vector $u_{i}$, followed by greedy selection. If $u_{i}$ is better, it replaces $x_{i}$ in the next generation, and the old $x_{i}$ is added to the archive.

Successful $F_{i}$ and $CR_{i}$ values (those yielding improvement) are collected in lists $S_{F}$ and $S_{CR}$. At the end of each generation, if improvements occurred, a memory entry is updated as follows:

(8) $$M_{F,k}=\frac{\sum_{l=1}^{|S_{F}|}\omega_{l}\;S_{F,l}^{2}}{\sum_{l=1}^{|S_{F}|}\omega_{l}\;S_{F,l}},$$

(9) $$M_{CR,k}=\sum_{l=1}^{|S_{CR}|}\omega_{l}\;S_{CR,l},$$

where the weights are

(10) $$\omega_{l}=\frac{\Delta f_{l}}{\sum_{m}\Delta f_{m}},\;\Delta f_{l}=\left|f\left(u_{l}\right)-f\left(x_{l}\right)\right|.$$

The weighted Lehmer mean is used for $M_{F,k}$ to favor larger scaling factors, while the weighted arithmetic mean is applied to $M_{CR,k}$. If no successful CR values exist in a generation, the corresponding $M_{CR,k}$ is set to −1.

This mechanism, combined with the external archive, enables effective self-adaptation and sustained population diversity. The core loop is presented in Algorithm 2.
**Algorithm 2** Pseudocode of SHADE (core loop) 1: Initialize population of fixed size *N*, memory $M_{F},M_{CR}$, archive A=∅ 2: **while** termination not met **do** 3:  **for** each individual *i* **do** 4:   Select memory index *k*, generate $F_{i}=\mathrm{randc}\left(M_{F,k},0.1\right)$, $CR_{i}=\mathrm{randn}\left(M_{CR,k},0.1\right)$ 5:   Select p∈[0.05,0.2], $x_{pbest}$ from top p·N individuals 6:   Select $x_{r1}\neq i$ from population, $x_{r}$ from population ∪ archive 7:   Mutant: $v_{i}=x_{i}+F_{i}\left(x_{pbest}-x_{i}\right)+F_{i}\left(x_{r1}-x_{r}\right)$ 8:   Binomial crossover with $CR_{i}$ to produce trial $u_{i}$ 9:   Greedy selection, update archive and success lists10:  **end for**11:  Update memory with weighted Lehmer mean of successful *F* and arithmetic mean of successful CR12: **end while**

## 3. Proposed HALA Algorithm

This section presents the detailed framework and mechanisms of the proposed HALA. First, the overall framework is outlined. Then, the evolution procedures of the two subpopulations are described in detail, with particular emphasis on the novel enhancement to ALA. Finally, the bidirectional elite migration strategy is elaborated, followed by key advantages and time complexity analysis.

### 3.1. Overall Framework of HALA

**Figure 1:**
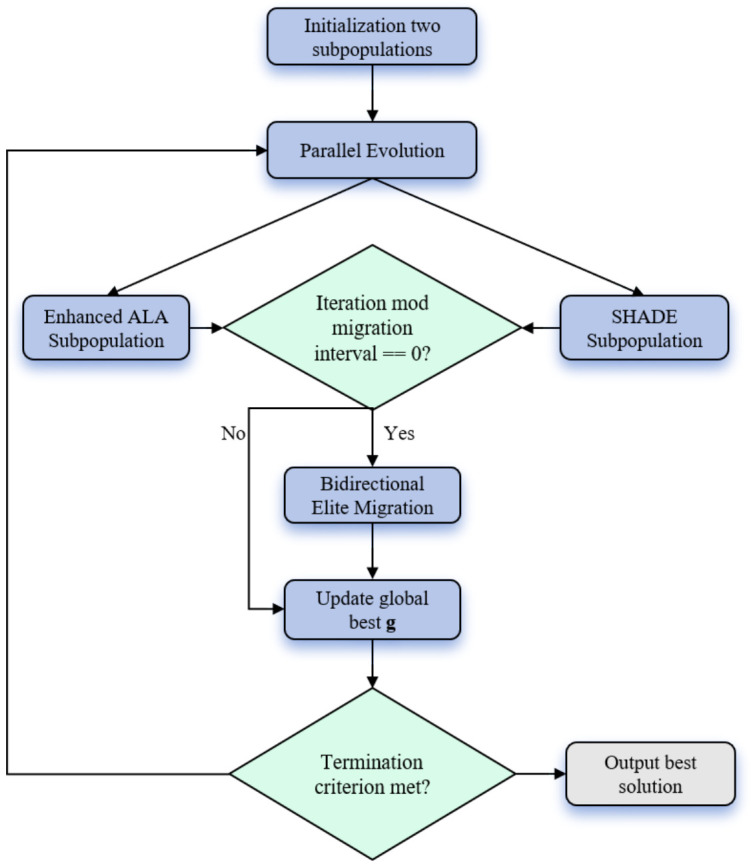
Flowchart of the proposed HALA.

The overall framework of HALA is illustrated in Figure 1 and formalized in Algorithm 3. The total population is evenly divided into two fixed-size subpopulations of size sub_N≈N/2. The first subpopulation employs an enhanced version of ALA to sustain strong global exploration and population diversity throughout the optimization process. The second subpopulation utilizes SHADE with fixed population size to provide precise local refinement capabilities.

At each iteration, the two subpopulations evolve independently using their respective update mechanisms. Information exchange is facilitated by a periodic bidirectional elite migration strategy (every migration_interval=20 generations). This cooperative design allows the enhanced ALA subpopulation to inject heavy-tailed disturbances into SHADE, preventing stagnation in local optima, while SHADE supplies high-quality elite solutions to the enhanced ALA subpopulation, accelerating convergence and mitigating premature diversity loss. The global best solution g is continuously updated across both subpopulations.
**Algorithm 3** Framework of the Proposed HALA Algorithm**Require:** Objective function fobj, dimension *D*, bounds lb, ub, population size *N*, maximum iterations $T_{\max}$**Ensure:** Best score Score, best position g 1: sub_N←⌊N/2⌋ 2: Initialize subpopulation $X_{1}$ (enhanced ALA) and $X_{2}$ (SHADE), each of size sub_N 3: Evaluate fitness and determine initial global best g 4: Initialize SHADE memory $M_{F}$, $M_{CR}$, archive A←∅ 5: **for** t=1 to $T_{\max}$ **do** 6:  **// Enhanced ALA subpopulation evolution:** 7:  $X_{1}\leftarrow$ Enhanced_ALA_Evolution($X_{1}$, g, *t*, $T_{\max})\;$▹ See Algorithm 4 8:  **// SHADE subpopulation evolution:** 9:  ($X_{2}$, *A*, $M_{F}$, $M_{CR}$) ← SHADE_Evolution($X_{2}$, *A*, $M_{F}$, $M_{CR})\;$▹ Algorithm 510:  **if** tmodmigration_interval=0 **then**11:   **// Bidirectional elite migration:**12:   Exchange elite individuals between $X_{1}$ and $X_{2}\;$▹ See Algorithm 613:  **end if**14:  Update global best g and Score from the combined population $X_{1}\cup X_{2}$15: **end for**16: **return** Score, g

### 3.2. Enhanced ALA Subpopulation Evolution

The first subpopulation is dedicated to maintaining robust global exploration throughout the optimization process. To mitigate the original ALA’s rapid decay of heavy-tailed behavior in the late stages—caused by the linearly decreasing Levy step size in the predator evasion phase—we introduce the Hybrid *t*-Levy Disturbance Strategy. This strategy replaces the standard Levy flight with a composite perturbation that blends the Student’s *t*-distribution and Levy flight, significantly extending heavy-tailed jumps and enhancing late-stage diversity.

**Hybrid *t*-Levy Disturbance Strategy:** In the low-energy phase (E≤1) when predator evasion is activated, the position update is governed by:

(11) $$X_{i}^{\mathrm{new}}=g+F\cdot \alpha \cdot \sigma \cdot \left(g-X_{i}\right),$$

where

- g is the current global best position,
- F=±1 is a random direction flag,
- $\alpha =1.5(1-t/T_{\max})$ is the adaptive step size factor (linearly decaying),
- $\nu =10(1-t/T_{\max})+1$ is the adaptive degrees of freedom (decreasing from 10 to 1),
- σ=β·t(ν)+(1−β)·Lévy(D) is the hybrid heavy-tailed step, with fixed mixing ratio β=0.7.

The *t*-distribution component (controlled by ν) provides extremely heavy tails in early iterations, while the Levy component preserves long jumps later. The bias β=0.7 favors the *t*-distribution initially, ensuring prolonged exploration capability. All other behaviors (high-energy migration/digging and spiral foraging) remain identical to the original ALA (Algorithm 1).

The complete procedure for the enhanced ALA subpopulation evolution, incorporating the Hybrid *t*-Levy Disturbance Strategy (Equation (11)), is detailed in Algorithm 4.
**Algorithm 4** Enhanced_ALA_Evolution**Require:** Current enhanced ALA subpopulation $X_{1}$ (size sub_N), global best g, current iteration *t*, max iterations $T_{\max}$, bounds lb, ub**Ensure:** Updated subpopulation $X_{1}$ 1: Compute $\theta =2\arctan(1-t/T_{\max})$ 2: **for** each individual i=1 to sub_N **do** 3:  Generate $RB_{i}∼N{\left(0,1\right)}^{D}$, flag F=±1 4:  E=2log(1/rand)·θ 5:  **if** E>1 **then** 6:   Perform high-energy exploration (migration or digging as in original ALA, Algorithm 1) 7:  **else** 8:   **if** rand<0.5 **then** 9:    Perform spiral foraging movement (as in original ALA, Algorithm 1)10:   else▹ Hybrid *t*-Levy Disturbance Strategy11:    $\nu =10\;\cdot \;(1-t/T_{\max})+1$12:    t_step∼t(ν), levy_step=Levy(D)13:    σ=0.7·t_step+0.3·levy_step14:    $\alpha =1.5\cdot (1-t/T_{\max})$15:    $X_{i}^{\mathrm{new}}=g+F\cdot \alpha \cdot \sigma \cdot \left(g-X_{i}\right)\;$▹ Equation (11)16:   **end if**17:  **end if**18:  Bound $X_{i}^{\mathrm{new}}$ within [lb,ub], evaluate fitness, greedy selection19: **end for**20: **return** Updated $X_{1}$

### 3.3. SHADE Subpopulation Evolution

The second subpopulation employs SHADE [20] to preserve stable diversity. The SHADE subpopulation evolution procedure is encapsulated in Algorithm 5, which performs parameter adaptation, mutation, crossover, selection, and updates to the archive and memory.
**Algorithm 5** SHADE_Evolution**Require:** Current SHADE subpopulation $X_{2}$ (size sub_N), external archive *A*, memory $M_{F}$, $M_{CR}$, bounds lb, ub**Ensure:** Updated subpopulation $X_{2}$, updated archive *A*, updated memory $M_{F}$, $M_{CR}$ 1: **for** each individual i=1 to sub_N **do** 2:  Randomly select memory index *k* 3:  Generate $F_{i}=\mathrm{randc}\left(M_{F,k},0.1\right)$ 4:  Generate $CR_{i}=0$ if $M_{CR,k}=-1$, else $\mathrm{randn}(M_{CR,k},0.1)$ 5:  Select p∈[0.05,0.2] and $x_{pbest}$ from top p·sub_N individuals 6:  Select $x_{r1}\neq i$ from $X_{2}$ and $x_{r}$ from $X_{2}\cup A$ 7:  Generate mutant $v_{i}=x_{i}+F_{i}\left(x_{pbest}-x_{i}\right)+F_{i}\left(x_{r1}-x_{r}\right)$ 8:  Apply binomial crossover with $CR_{i}$ to produce trial $u_{i}$ 9:  Bound $u_{i}$ within [lb,ub] and evaluate its fitness10:  **if** fitness of $u_{i}$ better than fitness of $x_{i}$ **then**11:   Replace $x_{i}\leftarrow u_{i}$12:   Record successful $F_{i}$ and $CR_{i}$ in success lists $S_{F}$, $S_{CR}$13:   Update archive *A* with old $x_{i}$14:  **end if**15: **end for**16: **if** success lists non-empty **then**17:  Update selected memory entry: $M_{F,k}$ using weighted Lehmer mean of $S_{F}$, and $M_{CR,k}$ using weighted arithmetic mean of $S_{CR}$ (or set to −1 if $S_{CR}=\emptyset$)18: **end if**19: **return** Updated $X_{2}$, *A*, $M_{F}$, $M_{CR}$

By maintaining fixed size, this variant integrates seamlessly into the dual-population framework and provides stable, high-precision local refinement.

### 3.4. Bidirectional Elite Migration Strategy

The core innovation of HALA lies in the periodic bidirectional elite migration between subpopulations, which facilitates collaborative search and balances exploration–exploitation dynamically.

Every migration_interval generations (set to 20 in experiments), the following operations are performed:

1. Identify the worst individual in enhanced ALA subpopulation: $worst1=\arg\max(fit_{1})$.
2. Replace it with the current best individual from SHADE subpopulation.
3. Identify the worst individual in SHADE subpopulation: $worst2=\arg\max(fit_{2})$.
4. Replace it with the current best individual from enhanced ALA subpopulation.

Reevaluate fitness after each replacement. This mechanism injects high-precision solutions from SHADE into enhanced ALA and exploratory disturbances from enhanced ALA into SHADE.

The migration process is detailed in Algorithm 6.
**Algorithm 6** Bidirectional Elite Migration**Require:** Enhanced ALA subpopulation $X_{1}$ with fitness $fit_{1}$, SHADE subpopulation $X_{2}$ with fitness $fit_{2}$**Ensure:** Updated subpopulations $X_{1}$, $X_{2}$ 1: Find worst index in enhanced ALA: $worst1\leftarrow \arg\max(fit_{1})$ 2: Find best position in SHADE: best2← current best in $X_{2}$ 3: $X_{1}\left(worst1,:\right)\leftarrow best2$; reevaluate fitness 4: Find worst index in SHADE: $worst2\leftarrow \arg\max(fit_{2})$ 5: Find best position in enhanced ALA: best1← current best in $X_{1}$ (post-update) 6: $X_{2}\left(worst2,:\right)\leftarrow best1$; reevaluate fitness 7: **return** Updated $X_{1}$, $X_{2}$

### 3.5. Key Advantages and Time Complexity Analysis

The proposed HALA framework offers several key advantages:

- The enhanced ALA subpopulation, equipped with the Hybrid *t*-Levy Disturbance Strategy, provides continuous and powerful global exploration with sustained heavy-tailed behavior throughout the optimization process.
- The SHADE subpopulation delivers stable, high-precision local refinement without the risk of premature diversity loss associated with population size reduction.
- The bidirectional elite migration strategy enables efficient, low-overhead knowledge transfer between subpopulations, ensuring dynamic balance between exploration and exploitation.

Regarding computational complexity, initialization requires O(N). Each iteration involves position updates and fitness evaluations for both subpopulations, costing O(ND+N). The migration strategy adds only 2 extra evaluations every 20 iterations, which is negligible in amortized analysis. Thus, the overall complexity of HALA is $O(N+T_{\max}\cdot N\cdot \left(D+1\right))$, asymptotically equivalent to that of standard ALA, SHADE, and L-SHADE (see Table 2).

### 3.6. Parameter Setting Rationale

The proposed HALA introduces several control parameters, including the migration interval (migration_interval=20), the mixing ratio (β=0.7) for the hybrid *t*-Lévy disturbance, the degrees-of-freedom schedule (ν:10→1), the adaptive step-size coefficient ($\alpha_{0}=1.5$), and the equal subpopulation division (sub_N=⌊N/2⌋). The settings of these parameters are determined through a series of preliminary experiments and grounded in the following design principles:

**Equal subpopulation division:** The total population size *N* is evenly divided between the two subpopulations. This symmetric allocation ensures that neither the exploration nor the exploitation component is prematurely deprived of search resources. Preliminary tests with asymmetric ratios (e.g., 3:7 and 7:3) showed no consistent improvement over the 5:5 split across the CEC2017 10-dimensional test bed; consequently, the equal division is adopted for its simplicity and robustness.

**Migration interval:** The bidirectional elite migration is performed every 20 generations. This value balances the frequency of information exchange: overly frequent migration (e.g., every 5–10 generations) causes premature convergence of the enhanced ALA subpopulation toward the elite solutions discovered by SHADE, whereas excessively sparse migration (e.g., every 50+ generations) fails to provide timely exploitation guidance. The value of 20 generations is consistent with the migration intervals reported in representative dual-population studies [16].

**Hybrid mixing ratio** β=0.7**:** The weight β=0.7 for the *t*-distribution component ensures that heavier-tailed perturbations dominate in the early-to-middle search phase, while the Lévy component (1−β=0.3) maintains occasional long-range jumps. Preliminary testing with β∈{0.3,0.5,0.7,0.9} indicated that β=0.7 yields the most stable mean rank on the CEC2017 30-dimensional benchmark. While an adaptive β schedule may offer marginal gains, a fixed ratio is preferred here to maintain methodological parsimony.

**Degrees-of-freedom schedule** $\nu =10(1-t/T_{\max})+1$**:** The schedule decreases ν from 10 to 1. A higher initial ν (close to Gaussian) provides stable exploratory perturbations, whereas ν→1 approaches the Cauchy distribution, yielding extremely heavy tails that assist late-stage escape from local optima. Alternative schedules (fixed ν=1 or fixed ν=10) were found to be less effective in preliminary tests.

**Step-size coefficient** $\alpha_{0}=1.5$**:** This value scales the magnitude of hybrid perturbations relative to the distance-to-best vector. Values below 1.0 produced insufficient perturbation strength, while values above 2.0 degraded convergence precision. The coefficient 1.5 offers a balanced amplitude comparable to the step-size scaling used in the original ALA predator evasion mechanism [14].

We acknowledge that an exhaustive ablation study isolating every design choice would offer deeper mechanistic insight. However, the parameter settings above are justified by preliminary empirical screening and methodological parsimony, following the common practice in the recent metaheuristic literature [14,17,20]. A systematic component-wise ablation is reserved for future work (see Section Limitations and Future Directions).

## 4. Numerical Experiments and Analyses

This section comprehensively evaluates the optimization performance of the proposed HALA on the IEEE CEC2017 and CEC2022 benchmark suites. HALA is compared with 17 state-of-the-art/high-performance metaheuristic algorithms. Experiments are conducted in MATLAB R2020a on a Windows 10 system equipped with an Intel Core i9-9900K CPU (3.60 GHz) and 32 GB RAM.

### 4.1. Benchmark Suites and Evaluation Metrics

The IEEE CEC2017 suite consists of 30 functions [32], categorized as unimodal (F1–F3), simple multimodal (F4–F10), hybrid (F11–F20), and composition (F21–F30). Function F2 is excluded due to numerical instability. Table 3 outlines the function names, dimensions, search domains, and theoretical values for these benchmark problems. The more recent CEC2022 suite comprises 12 challenging functions [33]: unimodal (F1), multimodal (F2–F4), hybrid (F5–F7), and composition (F8–F12). Detailed descriptions of the CEC2022 suite are provided in Table 4.

Primary metrics are the mean (Mean) and standard deviation (Std) of the best fitness values over 30 independent runs. Statistical significance is assessed using the Wilcoxon rank-sum test at significance level α=0.05, following the standard protocol widely adopted in metaheuristic benchmarking studies [14,21,34,35]. The symbols “+”, “≈”, and “−” indicate that HALA is statistically better than, equivalent to, or worse than the competitor, respectively. The Wilcoxon test is applied as a two-sided test; for the summary counts (+/≈/−), a “+” is recorded only when HALA achieves a better mean and the corrected *p*-value is below 0.05. The Friedman test [36] is employed to derive the overall ranking of all algorithms across the benchmark suite, where a lower mean rank indicates better performance.

Following the guidelines for statistical comparison of metaheuristics [34], we note that the Wilcoxon rank-sum test is performed pairwise between HALA and each competitor. While multiple comparisons are conducted across 17 competitors, the summary +/≈/− counts serve as descriptive indicators of relative performance rather than simultaneous inferential conclusions. The Friedman test, which inherently accounts for multiple algorithms, provides the primary basis for overall ranking inference.

### 4.2. Compared Algorithms and Parameter Settings

The numerical optimization performance of HALA is benchmarked against the following 17 advanced metaheuristics, categorized into two groups as follows:

- State-of-the-art meta-heuristics
  (1) Artificial Lemming Algorithm (ALA) [14]
  (2) Chaotic Quasi-Opppositional ALA (CQALA) [17]
  (3) Animated Oat Optimization Algorithm (AOOA) [37]
  (4) Boomerang Aerodynamic Ellipse Optimizer (BAEO) [38]
  (5) Birds of Prey-Based Optimization (BPBO) [39]
  (6) Cuckoo Catfish Optimizer (CCO) [40]
  (7) Chaotic Evolution Optimization (CEO) [41]
  (8) Distance-Fitness Learning Scheme (DFL) [42]
  (9) Dream Optimization Algorithm (DMOA) [43]
  (10) Dhole Optimization Algorithm (DHOA) [44]
  (11) Fungal Growth Optimizer (FGO) [45]
  (12) Kirchhoff’s Law Algorithm (KLA) [46]
  (13) Phototropic Growth Algorithm (PGA) [47]
  (14) Schrodinger Optimizer (SO) [48]
  (15) Stellar Oscillation Optimizer (SOO) [49]
- High-performance meta-heuristics
  (16) Success-History-Based Adaptive DE with Linear Population Size Reduction (LSHADE) [21]
  (17) LSHADE with Semi-Parameter Adaptation Hybrid with CMA-ES (LSHADE-SPACMA) [22]

These 17 algorithms represent a comprehensive selection of recent advancements and established high-performers in the field of meta-heuristic optimization. The first 15 are state-of-the-art meta-heuristics proposed in recent years (primarily 2025), which have gained considerable attention and have been applied to practical engineering optimization problems across diverse domains. The remaining two are high-performance algorithms that have consistently ranked as champions in CEC competitions, exhibiting exceptional performance on standard numerical optimization benchmarks.

By benchmarking the proposed HALA against this diverse and competitive set of algorithms—including both cutting-edge recent proposals and proven CEC winners—the comparison provides a more rigorous and comprehensive validation of HALA’s effectiveness and superiority.

The proposed HALA is a dual-population hybrid optimizer consisting of two fixed-size subpopulations, each with size ⌊N/2⌋. Information exchange occurs every 20 generations through bidirectional migration, where the worst individual in each subpopulation is replaced by the current best individual from the other subpopulation. The first subpopulation employs an enhanced version of ALA with the following settings: energy decay parameter $\theta =2\arctan(1-t/T_{\max})$; migration probability = 0.3; spiral foraging probability = 0.5; direction flag F=±1 (randomly selected); in the low-energy phase (E≤1), a mixed perturbation combining *t*-distribution and Levy flight is used (weights 0.7 for *t*-distribution and 0.3 for Levy), scaled by an adaptive factor $1.5(1-t/T_{\max})$ and *t*-distribution degrees of freedom decaying from 10 to 1 as $10(1-t/T_{\max})+1$. The second subpopulation uses a fixed-population-size variant of SHADE with memory size H=6, initial success-history memories $M_{F}=M_{CR}=0.5$, archive rate = 1.4 (maximum archive size = 1.4× subpopulation size), $p_{\mathrm{best}}$ rate = 0.11, and current-to-pbest/1/bin mutation strategy with external archive.

All 17 compared algorithms adhere to their original parameter settings, with the key parameters detailed in Table 5. To ensure a fair and unbiased comparison, common parameter values are adopted based on established findings in the literature [50], specifically setting the population size *N* to 30 and the maximum number of iterations $T_{\max}$ to 500. Tested dimensions are 10, 30, 50, 100 for CEC2017 and 10 for CEC2022. Each algorithm is executed 30 times independently.

**Fairness of comparison:** All compared algorithms are evaluated using the same maximum iteration budget ($T_{\max}=500$) and identical total population size (N=30), following the established benchmarking protocol in the recent metaheuristic literature [14,21]. HALA divides the total population into two subpopulations of size ⌊N/2⌋ each; therefore, the number of fitness evaluations per generation is comparable to that of any single-population algorithm. The marginal overhead introduced by bidirectional elite migration is analyzed in Section 4.5.

### 4.3. Experiment 1: Performance on CEC2017 Benchmark Suite

To demonstrate the effectiveness of the proposed HALA, the optimization performance of all aforementioned algorithms is evaluated on 29 functions from the CEC2017 benchmark suite (excluding F2) across multiple dimensions (10, 30, 50, and 100), including Qualitative Analysis, Quantitative Analysis, Convergence Analysis, Boxplot Analysis, and Scalability Analysis.

#### 4.3.1. Qualitative Analysis

This section provides a qualitative overview of the search behavior of the proposed HALA on six representative CEC2017 functions (F1, F7, F13, F17, F23, and F27) in 10 dimensions, based on search history, average fitness, trajectory in the first dimension, convergence curve, and exploration and exploitation trends.

**Figure 2:**
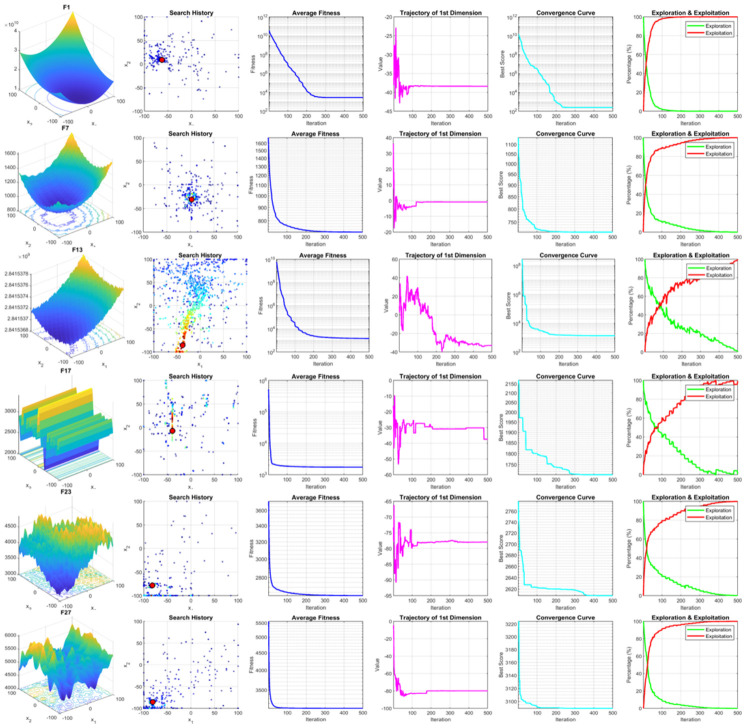
Qualitative visualization of HALA on selected CEC2017 functions in 10 dimensions, including search history, average fitness, trajectory in the first dimension, convergence curve, and exploration and exploitation trends.

The first column in Figure 2 displays the contour plots of the six selected CEC2017 benchmark functions in 10 dimensions (projected in 2D): one unimodal function (F1), one simple multimodal function (F7), two hybrid functions (F13 and F17), and two composition functions (F23 and F27). The contour of F1 shows a smooth, bowl-like structure leading to a single global optimum. In contrast, the remaining functions exhibit highly complex and rugged landscapes characterized by numerous local optima, basins of attraction, and deceptive features that pose significant challenges to optimization algorithms.

As shown in Figure 2, the search history (2nd column) demonstrates that HALA agents initially explore the search space extensively and gradually converge toward the vicinity of the global optimum. This pattern reflects the effective combination of the enhanced ALA subpopulation’s energy-driven random movements and the SHADE subpopulation’s differential evolution operators.

The average fitness curves (3rd column) decline rapidly at the beginning and continue to improve steadily, indicating quick population enhancement and sustained progress throughout the optimization process.

The trajectory in the first dimension (4th column) exhibits large fluctuations early on, followed by stabilization in later iterations, which highlights the transition from intensive global exploration to precise local exploitation.

The convergence curve (5th column) reveals fast and stable improvement in the best fitness value, with minimal stagnation even on complex multimodal, hybrid, and composition functions. This performance benefits from the complementary strengths of the dual subpopulations and periodic elite migration.

Finally, the exploration and exploitation trends (6th column) show high exploration rates in the initial phase, gradually shifting toward exploitation as iterations proceed. The smooth balance achieved by the hybrid framework allows HALA to thoroughly survey the search space while ensuring accurate convergence to high-quality solutions.

Overall, these qualitative results confirm that HALA maintains an effective balance between exploration and exploitation, contributing to its robust performance across functions of varying complexity.

#### 4.3.2. Quantitative Analysis

**Figure 3:**
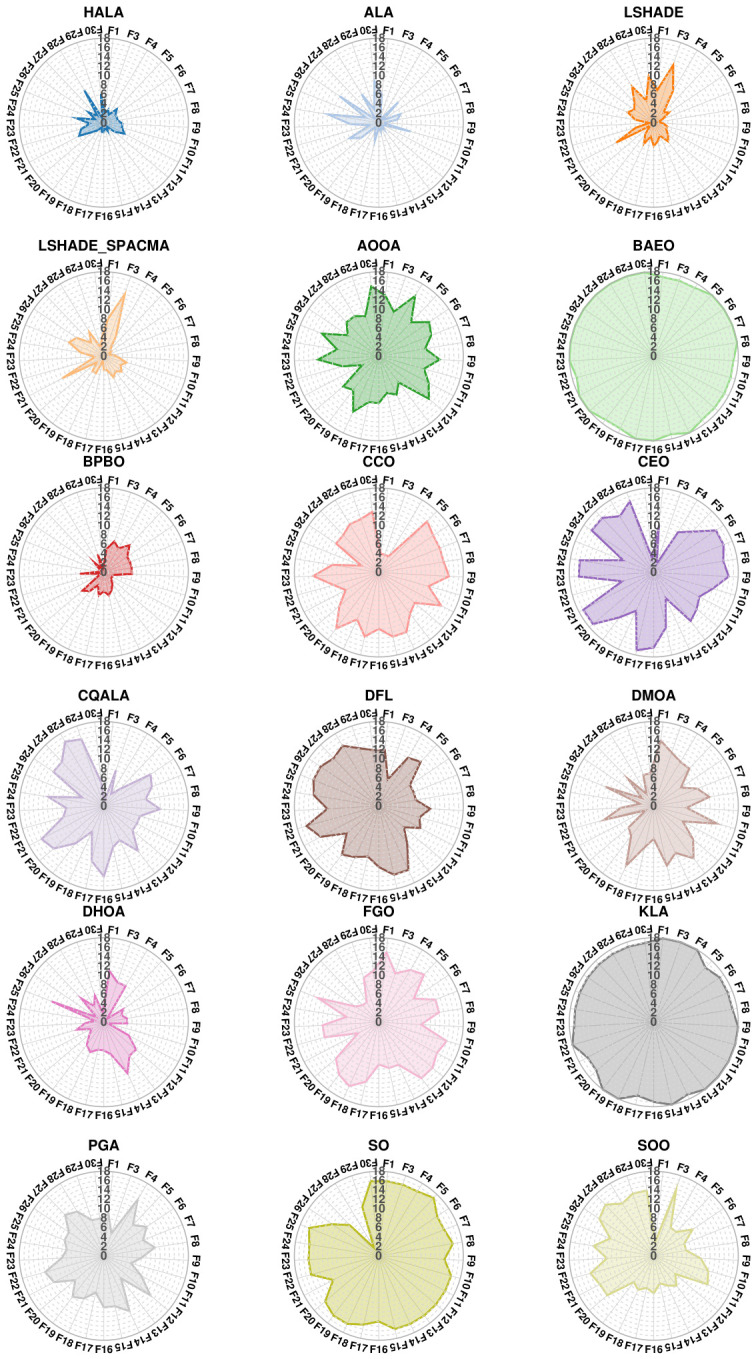
Radar plots of HALA and other algorithms on the CEC2017 test suite (Dim = 10).

This subsection compares HALA with ALA, LSHADE, LSHADE-SPACMA, AOOA, BAEO, BPBO, CCO, CEO, CQALA, DFL, DMOA, DHOA, FGO, KLA, PGA, SO, and SOO using 29 10-dimensional CEC2017 benchmark functions (excluding F2), and the statistical results are tabulated in Table A1 and Table A2. Based on the mean fitness (Mean), standard deviation (Std) and *p*-value, some observations are drawn as follows:

- For unimodal functions F1 and F3, CQALA achieves the best mean value on F1 (2.48 $\times \;10^{3}$ vs. 3.03 $\times \;10^{3}$ for HALA), indicating that CQALA’s chaotic initialization is particularly effective on this unimodal function. HALA ranks second and still outperforms the majority of competitors. On F3, ALA obtains the optimal mean value, while HALA ranks closely behind with nearly identical performance to several competitors. The adaptive convergence strategies in HALA provide high precision in unimodal landscapes, complemented by good stability.
- Regarding multimodal functions (F4–F10), HALA achieves the best or tied-best mean on F4 (tied with ALA) and F6 (tied with ALA and LSHADE-SPACMA). On the remaining functions, competitors such as LSHADE, LSHADE-SPACMA, ALA, BPBO, and DHOA exhibit superior or comparable results on several benchmarks (e.g., F5 and F8 for LSHADE/LSHADE-SPACMA, F7 for LSHADE-SPACMA, F9 for ALA, F10 for DHOA). HALA maintains competitive rankings and frequently low standard deviations, underscoring its effective exploration–exploitation balance and ability to escape local optima more reliably than lower-ranked algorithms.
- For hybrid functions (F11–F20), HALA delivers competitive solutions on numerous benchmarks, including top or tied-top mean values on F11 (tied with ALA, LSHADE, LSHADE-SPACMA, and BPBO), F13, F14 (tied with ALA), F15 (tied with ALA), F18, and F19 (tied with ALA). On F16 and F20, HALA ranks second (behind ALA on F16 and LSHADE on F20). HALA’s versatility and equilibrium between exploration and exploitation yield robust stability and accuracy across mixed landscapes.
- For composition functions (F21–F30), HALA secures top or near-top positions on multiple problems, with strong competitive results throughout (e.g., tied-best on F22 and F27, minimal gaps to winners on F23, F24, and F28). Competitors such as BPBO, DHOA, and LSHADE-SPACMA show strength on select benchmarks. These highly complex functions require superior global search and local optima avoidance, where HALA’s adaptive mechanisms demonstrate excellent tracking capability and robustness in shifted, rotated, and composite landscapes.
- Further, the +/≈/− row in Table A1 and Table A2 summarizes the outcomes of the Wilcoxon rank-sum test (based on *p*-values). HALA significantly outperforms its counterparts on the vast majority of the 29 benchmarks: 21 wins against ALA (with 5 approximate and 3 losses), 15–17 wins against LSHADE and LSHADE-SPACMA, and 27–29 wins against nearly all other 17 algorithms (with very few approximate or inferior cases). The last two rows present the global Friedman mean rankings across all 18 algorithms over the 29 functions. HALA achieves the lowest average rank of 2.55, securing the overall 1st place, followed by ALA and BPBO (tied at 3.52 for 2nd/3rd), LSHADE-SPACMA (3.90, 4th), and LSHADE (4.07, 5th), with DHOA ranking 6th (5.45). These statistical results, combined with the Wilcoxon outcomes, validate HALA’s superior overall performance. The radar ranking plots of HALA and the 17 competitor algorithms on the CEC2017 benchmark set (Figure 3) further confirm HALA’s minimal shaded area, indicating superior stability and comprehensive optimization capability across diverse landscapes. Overall, the results highlight HALA’s advantages in exploration, exploitation, and local optima avoidance.

#### 4.3.3. Convergence Analysis

**Figure 4:**
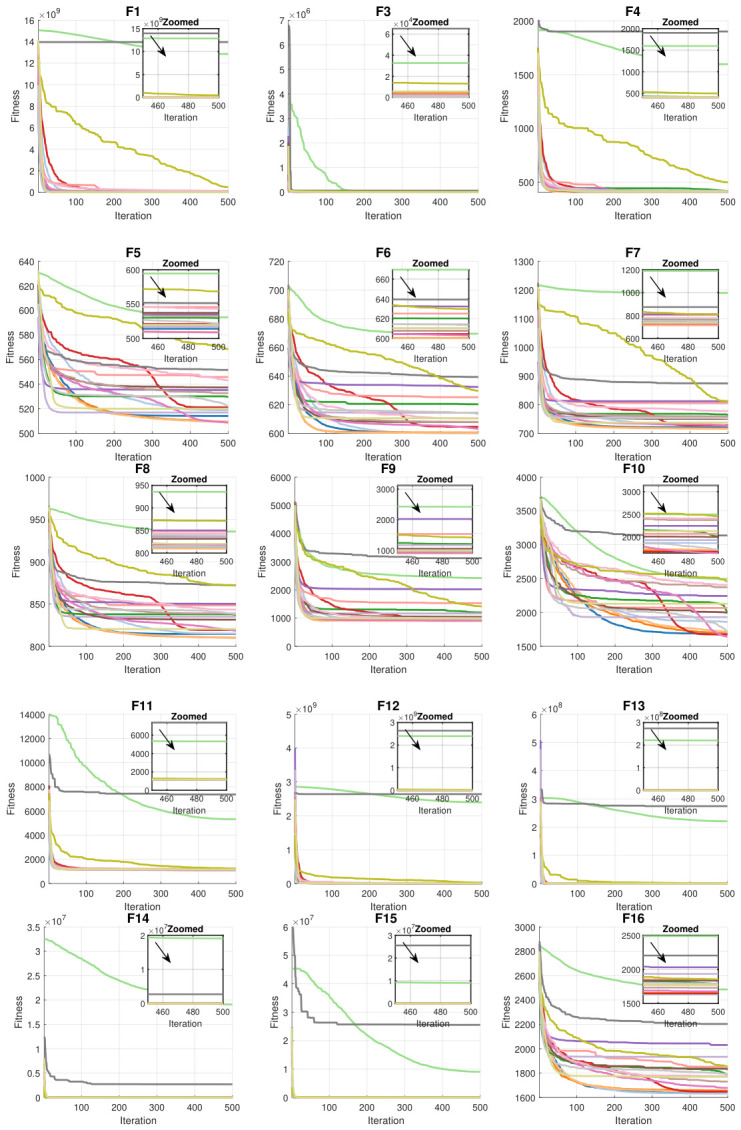
Convergence curves of HALA and other algorithms on unimodal (F1, F3), multimodal (F4–F10), and hybrid functions (F11–F16) from the CEC2017 benchmark suite (Dim=10).

**Figure 5:**
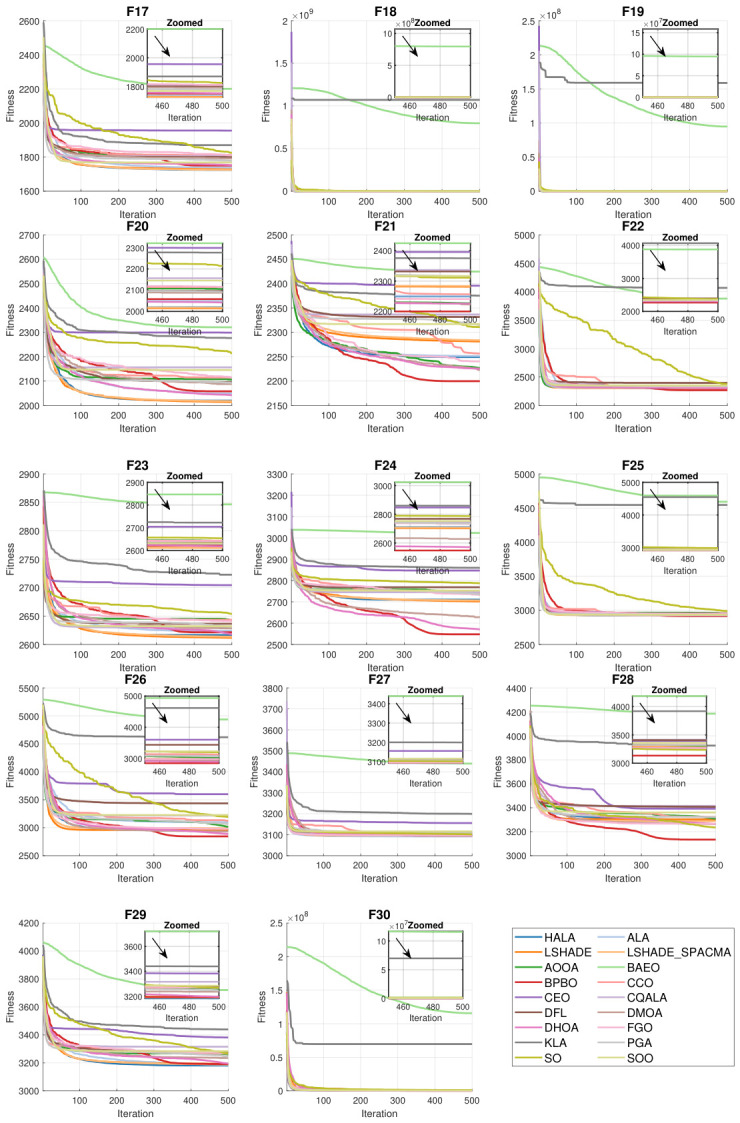
Convergence curves of HALA and other algorithms on the remaining hybrid (F17–F20) and composition functions (F21–F30) from the CEC2017 benchmark suite (Dim=10).

The convergence curves of HALA, ALA, LSHADE, LSHADE-SPACMA, AOOA, BAEO, BPBO, CCO, CEO, CQALA, DFL, DMOA, DHOA, FGO, KLA, PGA, SO, and SOO on 29 10-dimensional CEC2017 benchmark problems (excluding F2) are depicted in Figure 4 and Figure 5. For unimodal functions, HALA showcases remarkable convergence precision on both F1 and F3, with its profiles indicating a consistent downward path that emphasizes its strong exploitation features. On F1, HALA reaches lower fitness levels more efficiently than most counterparts, outperforming LSHADE-SPACMA, which gains early momentum but levels off at elevated values. Similarly, on F3, HALA maintains a competitive stance, closely matching or surpassing ALA’s stable convergence to low fitness values while surpassing approaches like AOOA and BAEO that plateau prematurely. This effectiveness stems from HALA’s adaptive techniques, which support detailed local optimization with minimal resource demands.

In multimodal functions, HALA upholds excellent competitiveness in terms of convergence pace and accuracy, particularly on F5, F7, F8, F9, and F10, where its paths decline rapidly to near-optimal outcomes, demonstrating effective exploration abilities. The tiered structure in HALA’s curves reveals its skill in breaking free from local minima, bolstered by its versatile population tactics. In comparison, methods such as CQALA and DFL advance quickly at the outset but often settle into less favorable areas. HALA falls slightly short of frontrunners on F4 and F6, yet the differences are modest but noticeable, while it leads decisively on F9. These findings confirm HALA’s balanced search approach, promoting diversity to maneuver through complex terrains more adeptly than standard techniques like PGA or SO.

For hybrid functions, as observed in F11, F13, F14, F15, and F19, HALA pinpoints viable zones within the first 100–200 iterations. It subsequently transitions from wide-ranging exploration to focused exploitation, improving solution sharpness. On F12 and F16, HALA’s outlines show the quickest and most dependable convergence among all participants, surpassing LSHADE-SPACMA despite its advanced fusions. Although placing second on F20, HALA’s sharpness remains notable and outperforms the majority of competitors. Rivals like KLA commonly halt early, whereas HALA evades this through its sturdy frameworks. This flexibility in addressing combined structures positions HALA as exceptionally capable, even in its basic configuration without additional elements.

Concerning composition functions, HALA’s optimization performance is generally robust. On F21, F22, F26, F27, F29, and F30, amid increased search space complexity, many algorithms face early halting, including top-performing LSHADE variants, which often face early halting, but HALA preserves steadiness and exactness in nearing the global optimum. For the other test cases, HALA generally exceeds peer methods to varying levels. The above experimental outcomes illustrate that HALA’s fundamental elements create a firm exploration–exploitation harmony, allowing it to achieve greater precision more swiftly and bypass local halts during optimization. Collectively, HALA provides highly competitive convergence patterns compared to other sophisticated meta-heuristic optimizers.

#### 4.3.4. Boxplot Analysis

**Figure 6:**
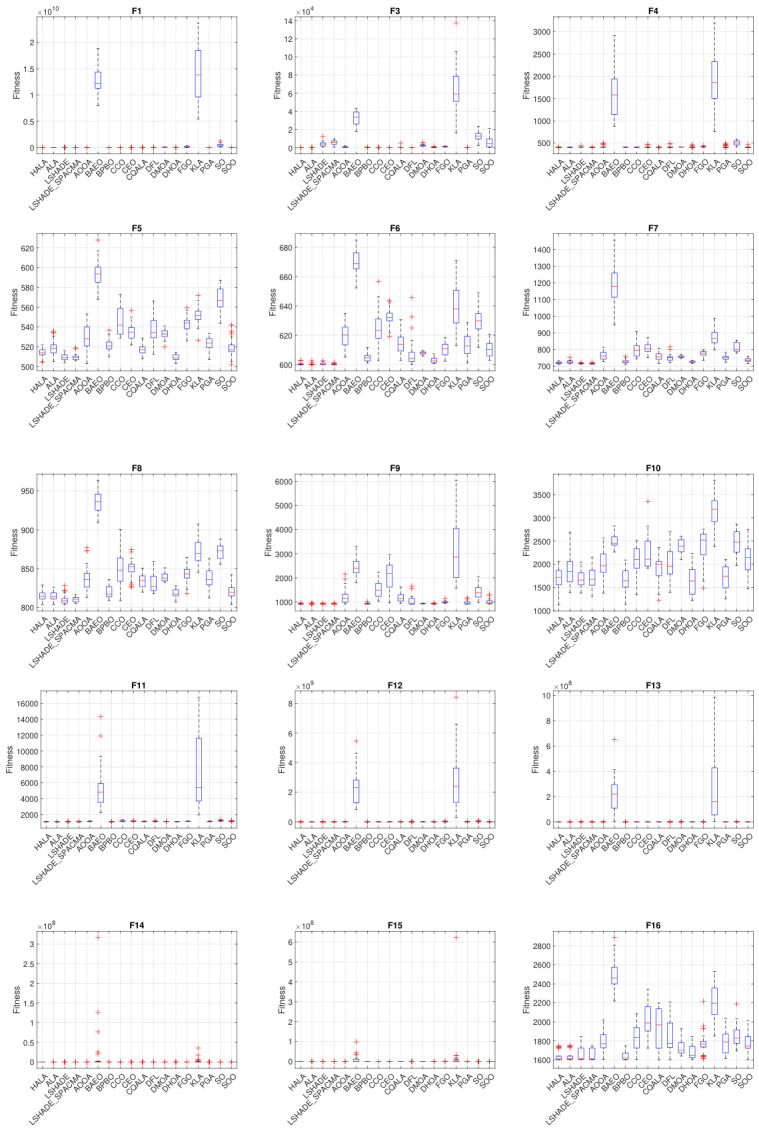
Boxplot of HALA and competing algorithms on unimodal (F1, F3), multimodal (F4–F10), and hybrid functions (F11–F16) from the CEC2017 benchmark suite (Dim=10).

**Figure 7:**
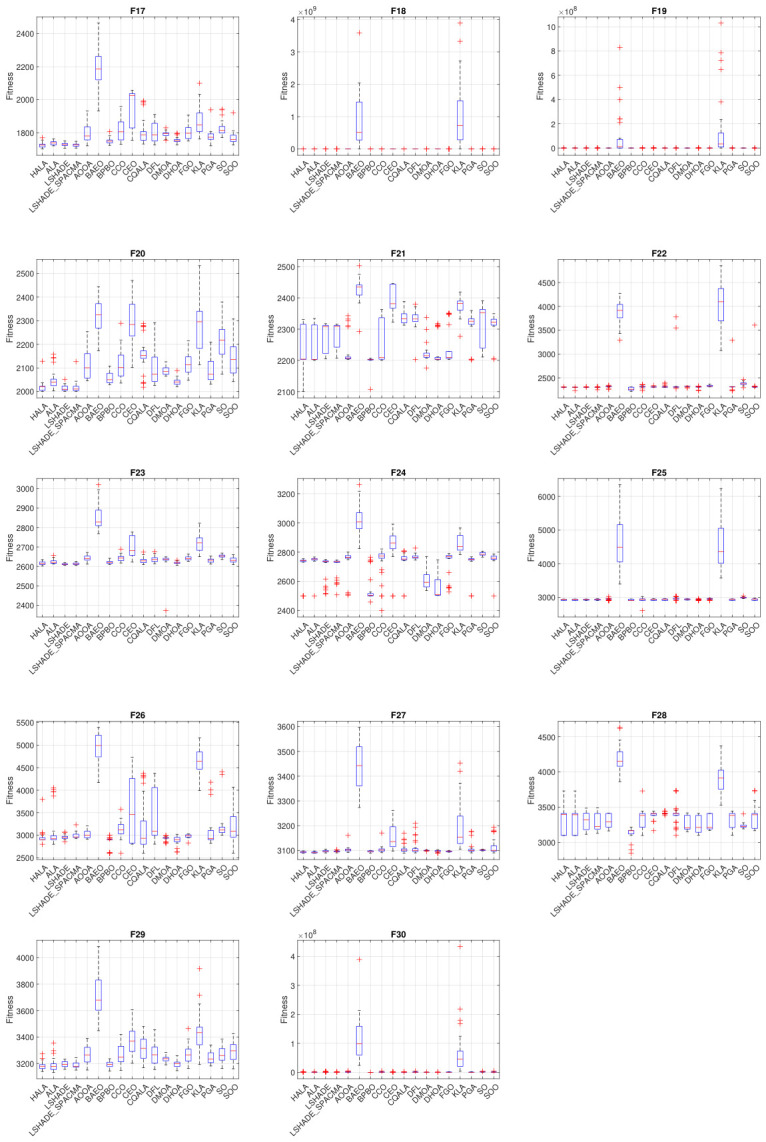
Boxplot of HALA and other algorithms on the remaining hybrid (F17–F20) and composition functions (F21–F30) from the CEC2017 benchmark suite (Dim=10).

Figure 6 and Figure 7 illustrate boxplots for HALA and the competing algorithms on selected benchmark functions, providing a visual representation of the fitness value distributions from 30 independent runs. In these boxplots, the central line denotes the median, the upper and lower edges of the box indicate the interquartile range, and the whiskers extend to the maximum and minimum values within 1.5 times the interquartile range. Outliers beyond this range are marked with a plus sign (“+”). The outcomes reveal that HALA’s maximum, minimum, and median values are more tightly clustered than those of other optimizers. Notably, on F1, F4, F6, F11, F13, F15, F18, F19, F22, F25, and F27, HALA produces no outliers and maintains superior consistency in convergence outcomes. These functions encompass unimodal, multimodal, hybrid, and composition categories, indicating that HALA not only handles straightforward numerical optimization tasks efficiently but also exhibits remarkable robustness when confronting complex problems. Particularly for F22, all comparison algorithms generate outliers, with significant spreads between upper and lower limits in methods like KLA and BAEO, whereas HALA converges steadily to a consistent optimal value. On F3, F9, F12, and F30, HALA also yields outliers, yet its overall data distribution holds clear advantages over peers. The boxplot findings underscore the outstanding robustness and precision of HALA.

#### 4.3.5. Scalability Analysis

Real-world optimization problems in engineering are often characterized by high dimensionality, which poses substantial challenges to algorithmic scalability and robustness. To systematically evaluate the performance of HALA under increasing problem complexity, experiments were conducted on 29 benchmark functions from the CEC2017 test suite with dimensionalities of 30, 50, and 100, resulting in a total of 87 test instances. The comparative performance of HALA against 17 competing algorithms is reported in Table A3, Table A4, Table A5, Table A6, Table A7 and Table A8. Based on the mean performance values, HALA achieves the best results on 36 out of 87 benchmark instances (41.4%), demonstrating competitive overall performance across different dimensional settings.

Concurrently, the Wilcoxon rank-sum test (i.e., *p*-values) results reported in the tables provide further statistical evidence of the competitive performance of HALA across different problem dimensionalities. For the 30-dimensional CEC2017 benchmark functions, HALA outperforms ALA on 9 benchmarks, LSHADE on 23 benchmarks, LSHADE-SPACMA on 22 benchmarks, AOOA on 29 benchmarks, BAEO on 29 benchmarks, BPBO on 20 benchmarks, CCO on 28 benchmarks, CEO on 24 benchmarks, CQALA on 17 benchmarks, DFL on 29 benchmarks, DMOA on 26 benchmarks, DHOA on 23 benchmarks, FGO on 28 benchmarks, KLA on 29 benchmarks, PGA on 29 benchmarks, SO on 29 benchmarks, and SOO on 27 benchmarks. For the 50-dimensional CEC2017 benchmark functions, HALA outperforms ALA on 9 benchmarks, LSHADE on 23 benchmarks, LSHADE-SPACMA on 22 benchmarks, AOOA on 29 benchmarks, BAEO on 29 benchmarks, BPBO on 20 benchmarks, CCO on 28 benchmarks, CEO on 24 benchmarks, CQALA on 17 benchmarks, DFL on 29 benchmarks, DMOA on 26 benchmarks, DHOA on 23 benchmarks, FGO on 28 benchmarks, KLA on 29 benchmarks, PGA on 29 benchmarks, SO on 29 benchmarks, and SOO on 27 benchmarks, while maintaining statistically superior or comparable performance against the remaining swarm intelligence and evolutionary algorithms on the majority of benchmark functions. For the 100-dimensional CEC2017 benchmark functions, HALA continues to demonstrate strong scalability. Specifically, HALA outperforms ALA on 12 benchmarks, LSHADE on 28 benchmarks, LSHADE-SPACMA on 27 benchmarks, AOOA on 29 benchmarks, BAEO on 29 benchmarks, BPBO on 22 benchmarks, CCO on 27 benchmarks, CEO on 20 benchmarks, CQALA on 19 benchmarks, DFL on 28 benchmarks, DMOA on 23 benchmarks, DHOA on 24 benchmarks, FGO on 29 benchmarks, KLA on 29 benchmarks, PGA on 27 benchmarks, SO on 29 benchmarks, and SOO on 26 benchmarks.

When aggregating the Wilcoxon rank-sum test results across all three dimensional settings, HALA demonstrates statistically significant superiority over ALA, LSHADE, and LSHADE-SPACMA on 30, 74, and 71 functions, respectively, while being significantly inferior on only 24, 6, and 4 functions. These results indicate that the number of benchmark instances on which HALA is statistically inferior remains limited as the dimensionality increases.

**Figure 8:**
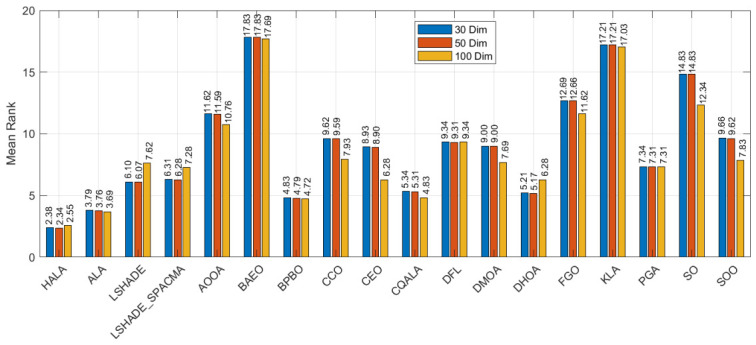
Friedman mean ranking results on IEEE CEC2017 benchmarks in 30, 50, and 100 dimensions.

The Friedman mean rank results shown in Figure 8 further support this observation, where HALA attains consistently lowest mean ranks of 2.38, 2.34, and 2.55 in the 30-, 50-, and 100-dimensional settings, respectively, confirming its stable scalability across different problem scales.

### 4.4. Experiment 2: Performance on CEC2022 Benchmark Suite

**Figure 9:**
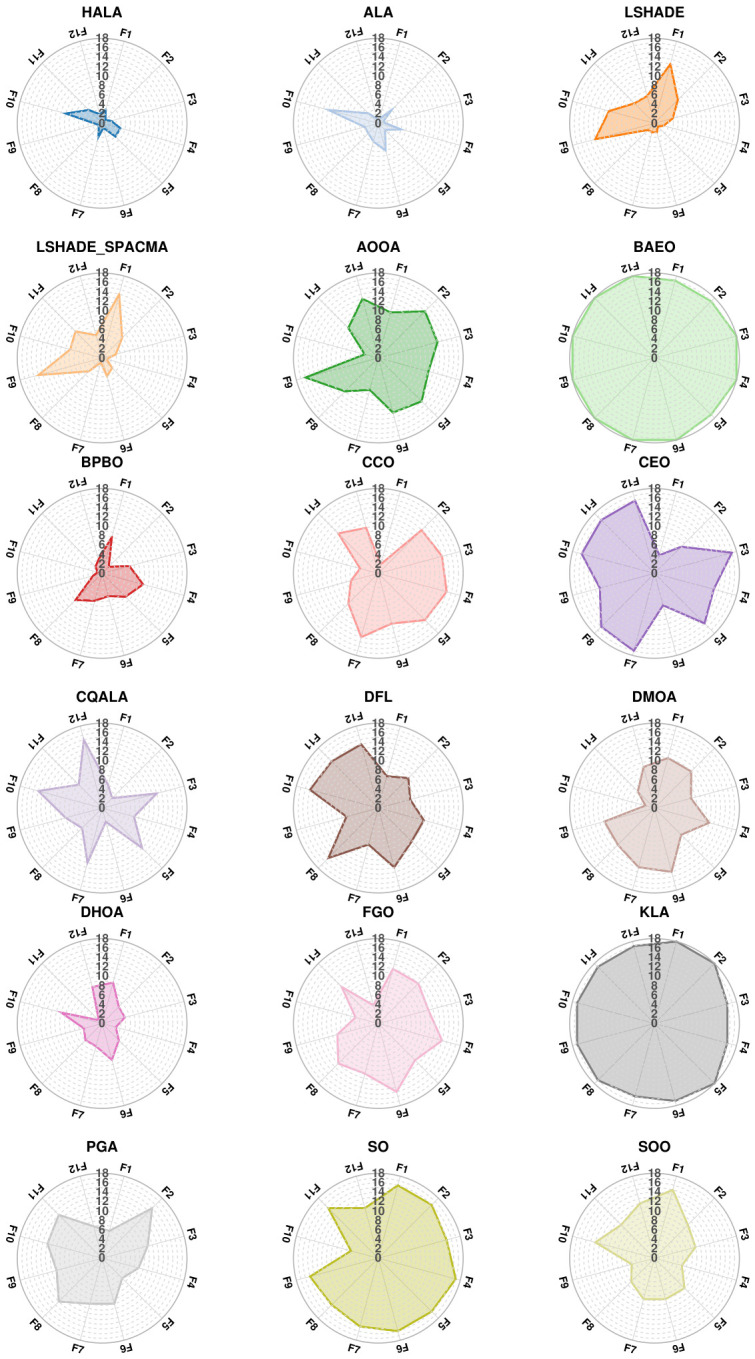
Radar plots of HALA and other algorithms on the CEC2022 test suite (Dim=10).

From these tables, HALA achieves the optimal average fitness value on 6 out of 12 CEC2022 functions (50%), specifically, F2 (solely), F3 (tied with ALA, LSHADE, and LSHADE-SPACMA), F6 (solely), F8 (solely), F9 (tied with ALA, BPBO, CCO, DHOA, and SOO), and F12 (tied with ALA, BPBO, and FGO). ALA shows the best performance on F5 (tied with LSHADE), LSHADE-SPACMA outperforms on F4 and F7 (tied on F7 with LSHADE), while DHOA excels on F11. These data indicate that HALA maintains a robust balance between exploration and exploitation even in the face of more complex search spaces, effectively escaping from local optimal stagnation. The Wilcoxon rank-sum test indicates that HALA outperforms ALA on 5 benchmarks, LSHADE on 7 benchmarks, LSHADE-SPACMA on 7 benchmarks, AOOA on 11 benchmarks, BAEO on 12 benchmarks, BPBO on 10 benchmarks, CCO on 10 benchmarks, CEO on 11 benchmarks, CQALA on 9 benchmarks, DFL on 12 benchmarks, DMOA on 11 benchmarks, DHOA on 9 benchmarks, FGO on 11 benchmarks, KLA on 12 benchmarks, PGA on 11 benchmarks, SO on 11 benchmarks, and SOO on 12 benchmarks. The Friedman test results suggest that HALA receives the best average ranking of 2.58, followed by ALA (3.17) and BPBO (4.00). Figure 9 presents the radar ranking map of each algorithm on 12 CEC2022 problems, where HALA ranks first with the smallest shaded area. These results again demonstrate the effectiveness of HALA and its reliable optimization performance in tackling complex optimization challenges.

**Figure 10:**
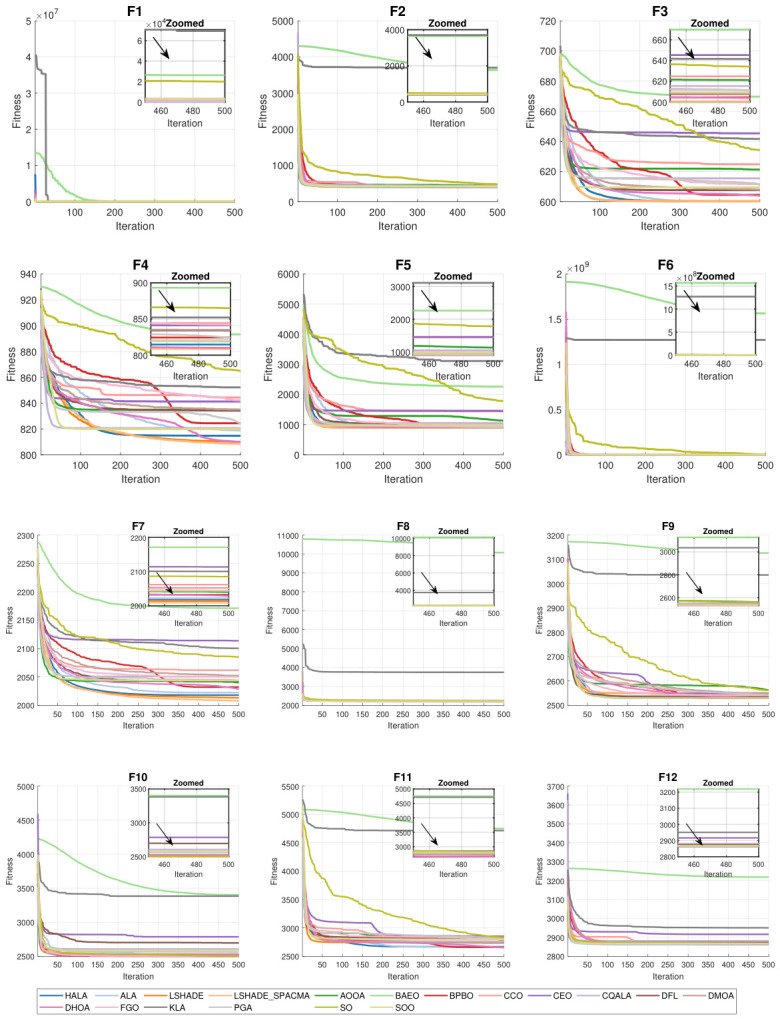
Convergence curves of HALA and other algorithms on the CEC2022 benchmark suite (Dim=10).

Figure 10 illustrates the convergence curves of HALA and other comparison algorithms. It is evident that HALA demonstrates superior convergence speed and accuracy across most test cases. For the unimodal function F1, HALA converges rapidly in the early iterations and achieves high final accuracy comparable to top performers such as ALA and CCO. On the simple multimodal function F2, CQALA exhibits the fastest initial decline, but HALA maintains a steady decreasing trend and reaches competitive, and arguably superior, final accuracy. For the multimodal function F3, HALA, ALA, LSHADE, and LSHADE-SPACMA show nearly identical convergence behavior with excellent precision, while HALA displays slightly faster progress in the early stage. In F4, LSHADE-SPACMA achieves the best final accuracy, yet HALA remains highly competitive with smooth convergence. For the hybrid functions F5–F7, HALA consistently outperforms most competitors in both speed and precision; particularly on F6, HALA dramatically surpasses all others, rapidly escaping potential local optima and reaching far superior solutions. On F7, LSHADE and LSHADE-SPACMA are slightly ahead in the very late iterations, but HALA still ranks among the leaders. Regarding the complex composition functions F8–F12, HALA exhibits remarkable efficiency: it quickly explores the search space in early iterations and smoothly transitions to fine exploitation, achieving the best or near-best final accuracy on F8, F9, F10, F11, and F12, often with the fastest convergence among top contenders. In contrast, algorithms such as BAEO, KLA, and SO frequently suffer from premature stagnation across multiple functions. All these findings confirm the outstanding convergence performance of HALA. The dual-population hybrid framework that integrates enhanced ALA’s global exploration with SHADE’s local refinement, combined with *t*-distribution/Lévy hybrid perturbations in the enhanced ALA subpopulation and bidirectional elite migration for dynamic diversity maintenance, not only enables HALA to effectively escape local optima but also significantly enhances its convergence speed and solution accuracy.

**Figure 11:**
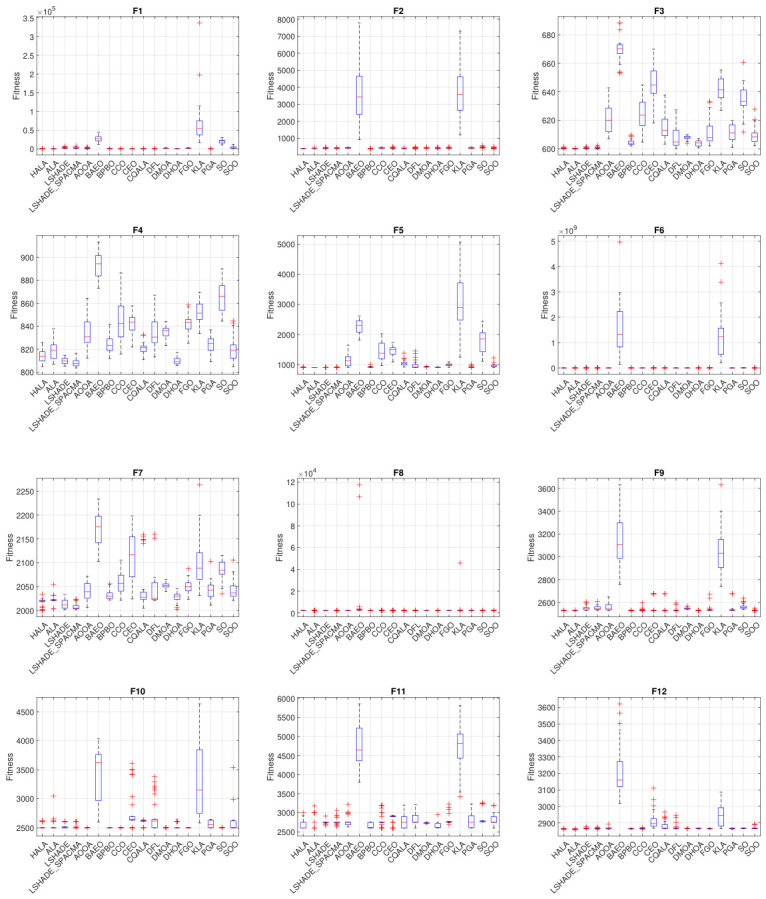
Boxplot of HALA and other algorithms on the CEC2022 benchmark suite (Dim=10).

Figure 11 illustrates the boxplots that showcase the distribution of fitness values achieved by HALA and other algorithms across 30 independent runs on the 12 CEC’2022 benchmark functions (D=10). The box for HALA is among the lowest positioned and relatively narrow on most functions, reflecting superior solution quality and higher stability compared to the majority of competitors. Although a few advanced competitors occasionally achieve lower medians on specific functions (e.g., F10 and F11), HALA remains highly competitive overall. Despite the inherent complexity of the search space making outliers unavoidable for many algorithms, HALA exhibits no visible outliers on F1, F2, F3, F4, F7, F8, F9, F10, and F12—a total of 9 out of 12 functions—while displaying only minimal outliers on the remaining ones (F5, F6, and F11). In contrast, most other algorithms show frequent and pronounced outliers across multiple functions. These observations further highlight HALA’s exceptional robustness, effectiveness, and reliable performance in addressing complex optimization problems.

This section presents a comprehensive analysis of the proposed method’s effectiveness on two IEEE CEC test sets, covering a total of 41 benchmark functions. The results demonstrate that HALA effectively outperforms other meta-heuristic algorithms across a range of benchmarks. The dual-population hybrid framework integrating enhanced ALA’s global search with SHADE’s local refinement, along with the incorporation of *t*-distribution/Levy hybrid perturbations in the enhanced ALA subpopulation and bidirectional elite migration for dynamic diversity maintenance, enables HALA to achieve higher accuracy and stability in finding global optima, particularly in complex, high-dimensional, and non-convex optimization problems.

### 4.5. Computational Cost Analysis

The theoretical time complexity of HALA is $O(N+T_{\max}\cdot N\cdot \left(D+1\right))$, asymptotically identical to that of ALA, SHADE, and L-SHADE (see Table 2). To assess the practical computational overhead introduced by the dual-population architecture, we measured the actual wall-clock runtime for all 18 algorithms over 30 independent runs on the CEC2017 30-dimensional benchmark suite. All experiments were executed on identical hardware: MATLAB R2020a running on an Intel Core i9-9900K CPU @ 3.60 GHz with 32 GB RAM.

As detailed in Table 6, HALA ranks 16th out of 18 algorithms in terms of wall-clock runtime (321.71 s), being approximately 2.5× slower than L-SHADE-SPACMA (127.26 s), 2.0× slower than SHADE (128.12 s), and 1.8× slower than its own exploration component ALA (179.49 s). This overhead is attributable to two factors: (1) maintaining and evolving a second subpopulation with full SHADE memory and archive updates, and (2) computing the hybrid *t*-Lévy perturbation and bidirectional elite migration at every 20 generations. Notably, HALA’s additional cost remains a constant-factor increase: the asymptotic complexity is unchanged, and the absolute runtime (under six minutes per run) is well within practical limits for offline engineering optimization.

The key question is whether this overhead is justified by performance gains. On the 30-dimensional CEC2017 suite, HALA achieves the best Friedman mean rank of 2.38, compared with 3.79 for ALA, 6.10 for L-SHADE, 6.31 for L-SHADE-SPACMA, and 5.34 for CQALA—the latter being 1.5× *slower* than HALA (493.17 s) yet performing substantially worse in ranking. Thus, HALA occupies a favorable position in the runtime–performance trade-off space: it is slower than minimal single-population algorithms but substantially faster than some complex hybrid competitors, while delivering the strongest overall optimization capability.

For applications where wall-clock time is critical, several avenues exist to reduce overhead without sacrificing the dual-population synergy, including asynchronous migration (decoupling subpopulation evolution cycles), adaptive migration frequency (reducing exchanges in late-stage convergence), and GPU-accelerated fitness evaluation. These directions are reserved for future investigation.

## 5. Applications of HALA in Real-World Engineering Problems

To further demonstrate the practical applicability and competitive performance of the proposed HALA, HALA and 17 competing algorithms are applied to five classical constrained engineering design problems. Since HALA is originally developed for unconstrained optimization, a standard penalty function method is adopted to handle constraints [51]. The constrained problem is transformed as follows:

(12) $$\min F\left(x\right)=f\left(x\right)+\sum_{i=1}^{p}a_{i}G_{i}\left(x\right)+\sum_{j=1}^{q}b_{j}H_{j}\left(x\right),$$

where

(13) $$G_{i}\left(x\right)=\max{\left(0,g_{i}\left(x\right)\right)}^{\eta },\;H_{j}\left(x\right)={\left|h_{j}\left(x\right)\right|}^{\lambda },$$

$g_{i}\left(x\right)$ and $h_{j}\left(x\right)$ are inequality and equality constraints, respectively, *p* and *q* are their counts, penalty coefficients $a_{i}$ and $b_{j}$ are positive constants $\left(a_{i}=b_{j}=10^{6}\right)$, η and λ are 1 or 2. For this penalty method, when a candidate solution violates any constraint, the objective function increases, thereby pushing the algorithm from the infeasible region into the feasible region.

For all problems, population size N=30, $T_{\max}=500$, and 30 independent runs are used. Results are compared with the 17 competing algorithms.

### 5.1. Pressure Vessel Design

**Figure 12:**
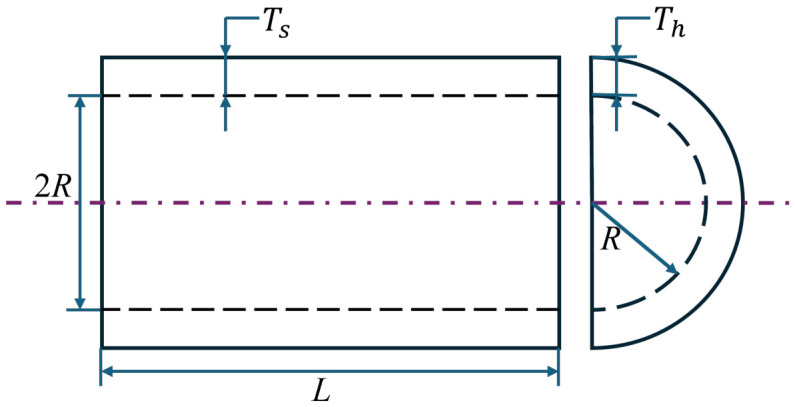
Pressure vessule design.

The objective of this problem is to minimize the fabrication cost of a pressure vessel [51] shown in Figure 12. There are four variables to be optimized. There are four constraints that need to be met in this problem. The problem formulation is presented below:

Consider variable $x=\left[x_{1},x_{2},x_{3},x_{4}\right]=\left[T_{s},T_{h},R,L\right]$.

Minimize $f_{1}\left(x\right)=0.6224x_{1}x_{3}x_{4}+1.7781x_{2}x_{3}^{2}+3.1661x_{1}^{2}x_{4}+19.84x_{1}^{2}x_{3}$.

Subject to

$$\begin{array}{cc} g_{1}\left(x\right) & =-x_{1}+0.0193x_{3}\leq 0,\\ g_{2}\left(x\right) & =-x_{2}+0.00954x_{3}\leq 0,\\ g_{3}\left(x\right) & =-\pi x_{3}^{2}x_{4}-\frac{4}{3}\pi x_{3}^{3}+1296000\leq 0,\\ g_{4}\left(x\right) & =x_{4}-240\leq 0, \end{array}$$

Variable range

$$\begin{array}{cc} \; & 0\leq x_{1}\leq 99,\\ \; & 0\leq x_{2}\leq 99,\\ \; & 10\leq x_{3}\leq 200,\\ \; & 10\leq x_{4}\leq 200. \end{array}$$

The statistical results of the optimal values and the optimal variables obtained by HALA and the comparative optimizers are presented in Table 7. Among all tested algorithms, HALA demonstrates highly competitive performance, achieving an excellent optimal function value $f_{1}=5885.3337409$ with the structure variables *x* = (0.778169, 0.384649, 40.319620, 199.999989). This result ranks fourth in terms of the best value and is extremely close to the top performers (DFL, CCO, and BPBO), outperforming the remaining algorithms. Compared to other modern optimizers such as ALA, FGO, SOO, CQALA, AOOA, CEO, BIHO, PGA, LSHADE, LSHADE-SPACMA, DHOA, SO, BTO, and DMOA, HALA consistently converges to a superior design region around x≈(0.77817,0.38465,40.32,200), yielding substantially lower fabrication costs. The results indicate that HALA possesses strong global exploration capability, enabling it to effectively escape local optima and identify near-optimal feasible structures that achieve remarkably low minimum fabrication costs.

### 5.2. Rolling Element Bearing Design

**Figure 13:**
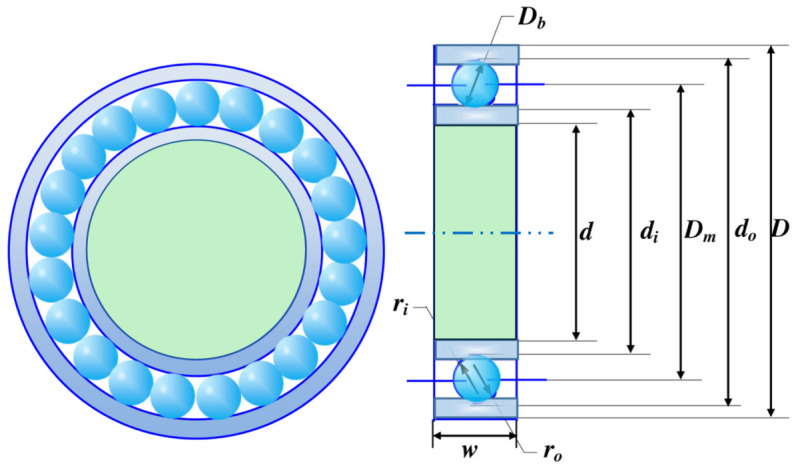
Rolling element bearing design.

This engineering case is the maximization of the dynamic load-carrying capacity of the rolling element bearing [52] shown in Figure 13. There are ten constraints and ten design variables. The problem formulation is presented below.

Consider variable $x=[D_{m},D_{b},Z,f_{i},f_{o},K_{D_{\min}},K_{D_{\max}},\varepsilon ,e,\zeta ]$.

Maximize

$$f_{2}\left(x\right)=\left\{\begin{array}{cc} f_{c}Z^{2/3}D_{b}^{1.8} & \mathrm{if}\;D_{b}\leq 25.4\;\mathrm{mm},\\ 3.647f_{c}Z^{2/3}D_{b}^{1.4} & \mathrm{if}\;D_{b}>25.4\;\mathrm{mm}, \end{array}\right.$$

Subject to

$$\begin{array}{cc} g_{1}\left(x\right) & =\frac{\phi_{o}}{2\sin^{-1}\left(D_{b}/D_{m}\right)}-Z+1\geq 0,\\ g_{2}\left(x\right) & =2D_{b}-K_{D_{\min}}\left(D-d\right)\geq 0,\\ g_{3}\left(x\right) & =K_{D_{\max}}\left(D-d\right)-2D_{b}\geq 0,\\ g_{4}\left(x\right) & =D_{m}-\left(0.5-e\right)\left(D+d\right)\geq 0,\\ g_{5}\left(x\right) & =\left(0.5+e\right)\left(D+d\right)-D_{m}\geq 0,\\ g_{6}\left(x\right) & =D_{m}-0.5\left(D+d\right)\geq 0,\\ g_{7}\left(x\right) & =0.5\left(D-D_{m}-D_{b}\right)-\varepsilon D_{b}\geq 0,\\ g_{8}\left(x\right) & =\zeta B_{w}-D_{b}\leq 0,\\ g_{9}\left(x\right) & =f_{i}-0.515\geq 0,\\ g_{10}\left(x\right) & =f_{o}-0.515\geq 0, \end{array}$$

where

$$\begin{array}{cc} f_{c} & =37.91{\left[1+{\left\{1.04{\left(\frac{1-\gamma }{1+\gamma }\right)}^{1.72}{\left(\frac{f_{i}\left(2f_{o}-1\right)}{f_{o}\left(2f_{i}-1\right)}\right)}^{0.41}\right\}}^{10/3}\right]}^{-0.3}\\ & \;\times \left(\frac{\gamma^{0.3}{\left(1-\gamma \right)}^{1.39}}{{\left(1+\gamma \right)}^{1/3}}\right){\left(\frac{2f_{i}}{2f_{i}-1}\right)}^{0.41}, \end{array}$$

$$\gamma =\frac{D_{b}}{D_{m}},\;f_{i}=\frac{r_{i}}{D_{b}},\;f_{o}=\frac{r_{o}}{D_{b}},$$

$$\phi_{o}=2\pi -2\cos^{-1}\left(\;\frac{{\left[\frac{D-d}{2}-\frac{3T}{4}\right]}^{2}+{\left[\frac{D}{2}-\frac{T}{4}-D_{b}\right]}^{2}-{\left[\frac{d}{2}+\frac{T}{4}\right]}^{2}}{2\left[\frac{D-d}{2}-\frac{3T}{4}\right]\left[\frac{D}{2}-\frac{T}{4}-D_{b}\right]}\right),$$

$$T=D-d-2D_{b},\;D=160,\;d=90,$$

$$\;B_{w}=30,\;r_{i}=r_{o}=11.033.$$

Variable range

$$\begin{array}{cc} \; & 0.5\left(D+d\right)\leq D_{m}\leq 0.6\left(D+d\right),\\ \; & 0.15\left(D-d\right)\leq D_{b}\leq 0.45\left(D-d\right),\\ \; & 4\leq Z\leq 50,\\ \; & 0.515\leq f_{i}\leq 0.6,\;0.515\leq f_{o}\leq 0.6,\\ \; & 0.4\leq K_{D_{\min}}\leq 0.5,\;0.6\leq K_{D_{\max}}\leq 0.7,\\ \; & 0.3\leq \varepsilon \leq 0.4,\;0.02\leq e\leq 0.1,\;0.6\leq \zeta \leq 0.85. \end{array}$$

The results of the optimal values and the optimal variables obtained by HALA and the comparative optimizers are presented in Table 8. Among all tested algorithms, HALA demonstrates outstanding performance, achieving the highest dynamic load-carrying capacity of 74647.473709 with the design variables *x* = (125.719056, 21.425590, 11, 0.515, 0.515, 0.400000, 0.646653, 0.300000, 0.100000, 0.612824). This result ties for the best value with ALA, BPBO, CCO, CEO, DFL, SOO, and CQALA, and clearly surpasses the remaining algorithms such as FGO, LSHADE, BIHO, LSHADE-SPACMA, PGA, DHOA, AOOA, DMOA, BTO, and SO. HALA successfully converges to the superior feasible region characterized by $D_{m}\approx 125.719$, $D_{b}\approx 21.426$, Z=11, and boundary values for inner/outer raceway grooves ($f_{i}=f_{o}=0.515$), yielding a significantly higher load-carrying capacity. These results highlight HALA’s exceptional global search ability and effectiveness in exploring the complex constrained search space, enabling it to consistently identify the optimal bearing design that maximizes dynamic load-carrying capacity.

### 5.3. Tension/Compression Spring Design

**Figure 14:**
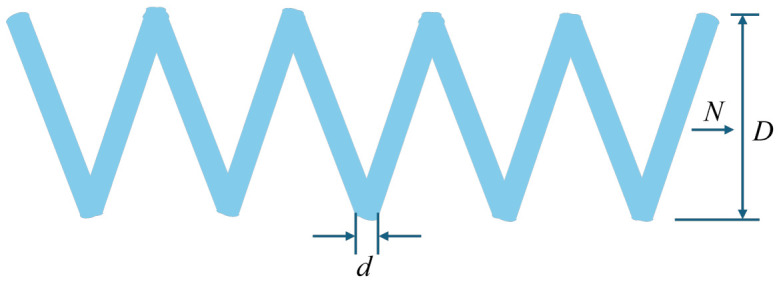
Tension/compression spring design.

This problem aims to minimize the weight of a tension/compression coil spring while satisfying three inequality constraints [53] shown in Figure 14. The problem formulation is presented below:

Consider variable $x=\left[x_{1},x_{2},x_{3}\right]=\left[d,D,N\right]$.

Minimize $f_{3}\left(x\right)=\left(x_{3}+2\right)x_{2}x_{1}^{2}$.

Subject to

$$\begin{array}{cc} g_{1}\left(x\right) & =1-\frac{x_{3}x_{2}^{3}}{71785x_{1}^{4}}\leq 0,\\ g_{2}\left(x\right) & =\frac{4x_{2}^{2}-x_{1}x_{2}}{12566(x_{2}x_{1}^{3}-x_{1}^{4})}+\frac{1}{5108x_{1}^{2}}-1\leq 0,\\ g_{3}\left(x\right) & =1-\frac{140.45x_{1}}{x_{2}^{2}x_{3}}\leq 0,\\ g_{4}\left(x\right) & =\frac{x_{1}+x_{2}}{1.5}-1\leq 0. \end{array}$$

Variable range $0.05\leq x_{1}\leq 2$, $0.25\leq x_{2}\leq 1.3$, $2\leq x_{3}\leq 15$.

The results of the optimal values and the optimal variables obtained by HALA and the comparative optimizers are presented in Table 9. Among all tested algorithms, HALA exhibits excellent performance, achieving one of the best optimal weights of 0.012666021011 with the design variables x=(0.051897,0.361749,11). This result ties for the top position with CQALA, SOO, and CCO, and is virtually identical to ALA and AOOA, demonstrating superior minimization of the spring weight compared to the remaining algorithms such as LSHADE, DFL, CEO, BPBO, PGA, LSHADE-SPACMA, FGO, DHOA, BTO, BIHO, SO, and DMOA. HALA consistently converges to the optimal feasible region characterized by wire diameter d≈0.051897, coil diameter D≈0.361749, and number of coils N=11, yielding a significantly lower weight. These results underscore HALA’s remarkable global optimization capability and robustness in handling the constrained search space, enabling it to reliably identify the minimum-weight design for the tension/compression spring.

### 5.4. Cantilever Beam Design

**Figure 15:**
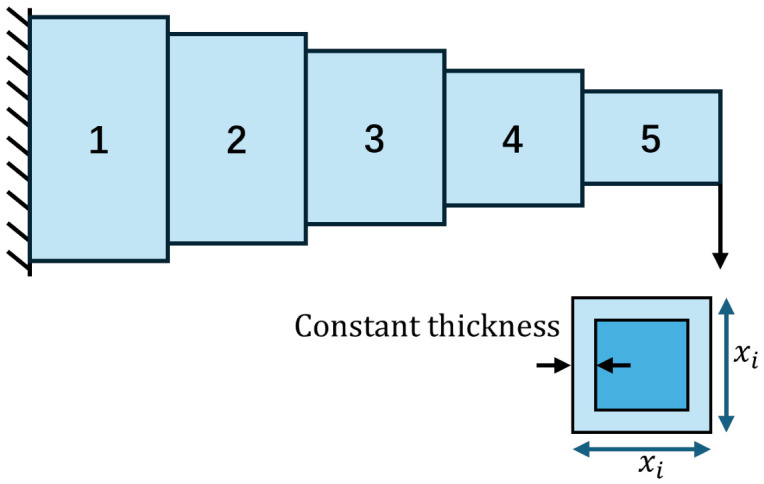
Cantilever beam design.

This problem aims to minimize the weight of a cantilever beam subject to a single vertical displacement constraint, where five design variables represent the heights of five hollow square blocks along the beam [54] shown in Figure 15. The problem formulation is presented below:

Consider variable $x=[x_{1},x_{2},x_{3},x_{4},x_{5}]$.

Minimize $f_{4}\left(x\right)=0.0624\left(x_{1}+x_{2}+x_{3}+x_{4}+x_{5}\right)$.

Subject to

$$g_{1}\left(x\right)=\frac{61}{x_{1}^{3}}+\frac{37}{x_{2}^{3}}+\frac{19}{x_{3}^{3}}+\frac{7}{x_{4}^{3}}+\frac{1}{x_{5}^{3}}-1\leq 0.$$

Variable range $0.01\leq x_{i}\leq 100$, i=1,…,5.

The results of the optimal values and the optimal variables obtained by HALA and the comparative optimizers are presented in Table 10. Among all tested algorithms, HALA delivers highly competitive performance, achieving one of the lowest optimal weights of 1.3399568320 with the design variables x=(6.017833,5.311599,4.493109,3.499130,2.151996). This result ranks second overall, extremely close to the top performer DFL and clearly superior to the remaining algorithms such as LSHADE, ALA, LSHADE-SPACMA, SOO, CCO, DHOA, CQALA, BTO, CEO, BPBO, AOOA, FGO, PGA, BIHO, DMOA, and SO. HALA successfully converges to the optimal feasible region characterized by block heights decreasing progressively from approximately $x_{1}\approx 6.018$ to $x_{5}\approx 2.152$, resulting in a significantly reduced total weight of the cantilever beam. These results demonstrate HALA’s strong global search capability and effectiveness in navigating the constrained design space, enabling it to reliably identify a near-optimal structure that minimizes the beam weight.

### 5.5. Gear Train Design

**Figure 16:**
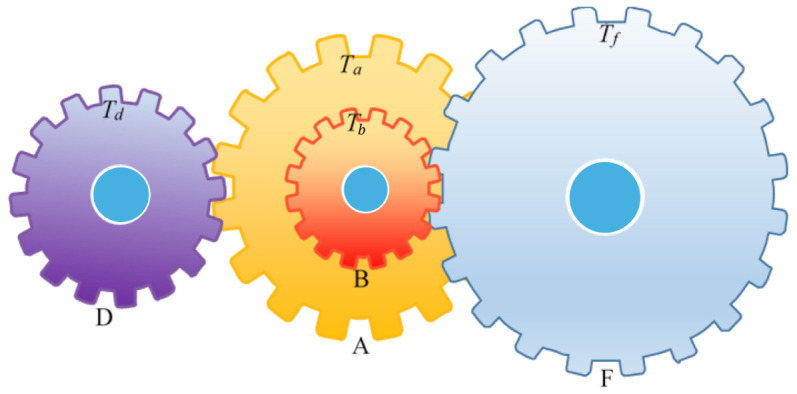
Gear train design.

The problem aims to minimize the cost of gear ratio of the gear train [55] shown in Figure 16. There are four integer variables in this problem, in which $T_{a}$, $T_{b}$, $T_{d}$, and $T_{f}$ represent the teeth number of four different gearwheels. The gear ratio is given as $\frac{T_{b}}{T_{a}}\cdot \frac{T_{d}}{T_{f}}$. The problem formulation is presented below:

Consider variable $x=\left[x_{1},x_{2},x_{3},x_{4}\right]=\left[T_{a},T_{b},T_{d},T_{f}\right]$.

Minimize

$$f_{5}\left(x\right)={\left(\frac{1}{6.931}-\frac{T_{b}\cdot T_{d}}{T_{a}\cdot T_{f}}\right)}^{2}.$$

Subject to (variable range) $0.01\leq x_{1},x_{2},x_{3},x_{4}\leq 60$.

The results of the optimal values and the optimal variables obtained by HALA and the comparative optimizers are presented in Table 11. Among all tested algorithms, HALA demonstrates superior performance, achieving the minimum gear ratio cost of $2.7008571489\times 10^{-12}$ with the gear teeth numbers x=(43,19,16,49). This optimal value ties for the best result with the majority of the compared algorithms (ALA, LSHADE, LSHADE-SPACMA, BIHO, BTO, BPBO, CCO, CQALA, DMOA, DHOA, FGO, and PGA), and significantly outperforms AOOA, DFL, SO, SOO, and CEO, which converge to higher costs on the order of $10^{-11}$ to $10^{-10}$. HALA reliably identifies one of the globally optimal integer combinations that yield the theoretically smallest deviation from the ideal gear ratio of 1/6.931. These results highlight HALA’s excellent discrete search capability and effectiveness in solving this combinatorial constrained optimization problem, enabling it to consistently reach the minimum possible cost for the gear train design.

These results highlight HALA’s superior performance and robustness in solving constrained real-world engineering problems.

## 6. Discussion

The comprehensive experimental results indicate that the proposed HALA exhibits competitive optimization performance across both numerical benchmarks and engineering applications, frequently ranking among the top algorithms in the comparison pool.

In numerical optimization experiments, HALA consistently ranks first on the CEC2017 and CEC2022 benchmark suites. On 29 10-dimensional CEC2017 functions (excluding F2), it achieves the lowest Friedman mean ranking of 2.55 and significant superiority over most competitors according to Wilcoxon tests. In higher dimensions (30, 50, and 100), HALA obtains the best results on 36 out of 87 instances (41.4%) with the lowest Friedman ranks (2.38, 2.34, and 2.55, respectively), confirming its excellent scalability. On the more challenging 10-dimensional CEC2022 suite, HALA secures the optimal mean on 6 out of 12 functions (50%) and the best Friedman ranking of 2.58. Qualitative visualizations, convergence curves, and boxplots further reveal that HALA maintains an effective exploration–exploitation balance, rapid convergence, high robustness, and strong local optima avoidance across diverse landscape complexities.

In real-world engineering applications involving five constrained design problems, HALA delivers excellent performance: near-optimal fabrication cost for pressure vessel design (ranking fourth), highest dynamic load-carrying capacity for rolling element bearing design (tying for best), minimal weight for tension/compression spring design (tying for best), near-minimal weight for cantilever beam design (ranking second), and global minimum gear ratio cost for gear train design (tying for best). These results highlight HALA’s ability to reliably identify superior feasible solutions in highly nonlinear and constrained search spaces.

The superior performance of HALA can be primarily attributed to its innovative dual-subpopulation hybrid framework. The enhanced ALA subpopulation provides powerful global exploration through energy-driven movements incorporating Brownian-like diffusion and Levy flight strategies, enabling thorough search space coverage and effective escape from local optima. Simultaneously, the SHADE subpopulation ensures precise local refinement via advanced differential evolution operators. Bidirectional elite migration and hybrid *t*-distribution/Levy perturbations maintain population diversity, promote information exchange, and facilitate smooth transitions between exploration and exploitation, preventing premature convergence even in complex multimodal and constrained landscapes.

### Limitations and Future Directions

While the experimental results support the effectiveness of HALA, several limitations of the current study should be acknowledged.

First, the hybrid framework relies on empirically determined control parameters (migration interval, mixing ratio, step-size coefficient). Although these parameters are justified by preliminary experiments and methodological considerations (Section 3.6), a systematic ablation study that isolates the marginal contribution of each component remains desirable for future work. Specifically, quantifying the individual contributions of the hybrid *t*-Lévy perturbation, bidirectional elite migration, and the dual-population architecture itself would strengthen the scientific rigor of the proposal.

Second, the current benchmark evaluation employs the same-iteration-stopping criterion ($T_{\max}=500$), which is consistent with the convention in the metaheuristic literature [14,20,21] but does not strictly control for the exact number of function evaluations. HALA’s bidirectional elite migration introduces a marginal evaluation overhead of less than 0.34%; however, the wall-clock runtime is necessarily higher than that of single-population algorithms due to the maintenance of two subpopulations and the hybrid perturbation computations. As reported in Table 6, HALA requires 321.71 s per run, compared with 179.49 s for ALA, 128.12 s for L-SHADE, and 127.26 s for L-SHADE-SPACMA—a constant-factor increase of approximately 1.8× versus ALA and 2.5× versus L-SHADE. While this overhead is justified by HALA’s superior Friedman ranking (2.38 vs. 3.79 for ALA and 6.10 for L-SHADE on 30-D CEC2017) and remains practical for offline engineering optimization (under six minutes per run), it may be prohibitive for time-critical real-time applications. Future work will investigate asynchronous migration strategies and adaptive migration frequency to reduce this overhead.

Third, HALA’s performance on high-dimensional unimodal functions (e.g., F1 and F3 at 100 dimensions) is occasionally surpassed by specialized algorithms such as L-SHADE or enhanced ALA variants, suggesting that the dual-population structure may introduce unnecessary exploration overhead on simpler landscapes where a single well-tuned algorithm suffices.

Fourth, the constraint-handling mechanism adopted in this study is a standard penalty function with fixed coefficients ($a_{i}=b_{j}=10^{6}$). While this approach is widely used in the engineering optimization literature, it does not adapt to constraint violation severity and may struggle with highly nonlinear or tightly constrained feasible regions. More sophisticated constraint-handling techniques, such as adaptive penalty methods or feasibility rules, may be needed for large-scale constrained optimization tasks.

Finally, the theoretical convergence properties of HALA have not been established. Unlike some pure evolutionary algorithms for which convergence proofs exist, the hybrid interaction between the ALA subpopulation (with heavy-tailed perturbations) and the SHADE subpopulation (with adaptive crossover and mutation) complicates formal analysis. This is a common limitation of hybrid metaheuristic approaches, and we identify theoretical convergence analysis as a long-term research goal.

Future research will prioritize: (1) systematic ablation studies with sensitivity plots for all fixed parameters; (2) MaxFEs-based benchmarking protocols for stricter computational fairness; (3) extensions to multi-objective, large-scale, and discrete optimization domains; (4) applications to economic dispatch and feature selection problems; and (5) theoretical investigation of convergence conditions under the dual-population framework.

Overall, HALA demonstrates competitive performance on standard numerical benchmarks and shows promise for practical engineering applications. These consistent advantages affirm HALA as a robust, efficient, and powerful metaheuristic optimizer suitable for a wide range of challenging applications.

## 7. Conclusions

This paper proposes a novel hybrid metaheuristic algorithm, termed the Hybrid Artificial Lemming Algorithm (HALA), for tackling complex optimization problems. HALA employs a dual-subpopulation architecture that combines an enhanced version of the Artificial Lemming Algorithm with the SHADE framework. The enhanced ALA subpopulation drives effective global exploration through energy-driven random walks and hybrid perturbation strategies, while the SHADE subpopulation delivers high-precision local search via adaptive differential evolution. Bidirectional elite migration ensures dynamic information sharing and sustained population diversity, resulting in a well-balanced exploration–exploitation process.

Extensive experiments on the CEC2017 benchmark suite across multiple dimensions (10, 30, 50, and 100) and the CEC2022 suite demonstrate that HALA achieves competitive solution quality and convergence characteristics relative to numerous advanced competitors, often attaining favorable rankings in statistical tests, including ALA, LSHADE, LSHADE-SPACMA, AOOA, BAEO, BPBO, CCO, CEO, CQALA, DFL, DMOA, DHOA, FGO, KLA, PGA, SO, and SOO. Statistical tests further validate its significant advantages on most functions. HALA also proves highly effective on five real-world constrained engineering design problems—pressure vessel, rolling element bearing, tension/compression spring, cantilever beam, and gear train—consistently delivering optimal or near-optimal designs that outperform or match the compared algorithms.

While HALA exhibits strong overall performance, it shares common limitations of hybrid metaheuristics, such as dependence on empirical validation rather than theoretical convergence proofs. Certain highly deceptive or ultra-high-dimensional problems may still offer room for improvement.

Future research will investigate self-adaptive control of subpopulation ratios and migration rates, incorporation of additional operators, and extensions to multi-objective, large-scale, and discrete optimization. Applications in emerging areas such as autonomous system planning, and task scheduling in cloud computing are also promising directions. In summary, HALA represents a robust and versatile optimizer capable of addressing a wide spectrum of challenging practical problems. The source code of HALA will be publicly available upon publication.

## Figures and Tables

**Table 1:** Summary of representative related algorithms and their key characteristics. Abbreviations: CC = cooperative co-evolution; MP = multi-population; DE = differential evolution.

Category	Representative Algorithm	Key Mechanism	Limitation	Distinction from HALA
ALA variants	CQALA [17]	Chaotic initialization + quasi-oppositional learning	Single-population; exploitation weakness persists; added mechanisms increase complexity without dedicated local search engine	HALA integrates a full SHADE subpopulation specifically for exploitation rather than adding auxiliary operators to a single population
	EALA [18]	Adaptive *t*-distribution perturbation + hybrid mutation strategy	Single-population; late-stage stagnation in high-dimensional multimodal landscapes due to lack of external exploitation pressure	HALA’s dual-population architecture prevents stagnation by injecting SHADE-refined elites via migration
	Enhanced ALA [19]	Dynamic exploration–exploitation balance mechanisms	Lacks a dedicated DE-based adaptive local search component; relies on internal parameter switching	HALA employs SHADE with success-history adaptation as an independent exploitation engine
SHADE and adaptive DE variants	L-SHADE [21]	Linear population size reduction (LPR) + memory-based parameter adaptation	Aggressive diversity loss due to LPR; no complementary global exploration mechanism outside DE framework	HALA retains SHADE’s fixed population size to preserve diversity and couples it with enhanced ALA for exploration
	L-SHADE-SPACMA [22]	Semi-parameter adaptation hybrid with CMA-ES	High computational overhead; complex multi-component integration difficult to extend	HALA achieves hybridization through simpler, low-overhead elite migration rather than algorithmic fusion
	jSO [23]	Adaptive *p*-best archiving + weighted mutation for bound constraints	Pure DE framework; conservative global search behavior in highly multimodal landscapes	HALA compensates SHADE’s conservative search via the enhanced ALA subpopulation
Cooperative co-evolution and MP frameworks	dp-ACS [16]	Dual-population co-evolution with dynamic resource allocation	Generic framework; specific algorithm pairing (two swarm methods) lacks complementary algorithmic paradigms	HALA specifically pairs a swarm intelligence method (ALA) with an adaptive DE variant (SHADE), combining fundamentally different search paradigms
	DECC-DG [24]	Differential grouping for large-scale CC optimization	Designed primarily for separability detection in large-scale problems; not a general-purpose hybridizer	HALA does not assume problem separability; targets general global optimization
	EPSDE [25]	Ensemble of mutation strategies and parameters in a single population	No explicit multi-population or exploration-exploitation division; ensemble operates homogeneously	HALA uses two heterogeneous, interacting subpopulations with distinct roles rather than an internal ensemble
Hybrid DE and ensemble frameworks	CoDE [26]	Composite DE with three fixed mutation strategies and associated parameters	Fixed strategy pool without online adaptation; single-population homogeneous search	HALA’s two subpopulations are algorithmically distinct (ALA vs. SHADE), not merely different DE variants
	MPEDE [27]	Multi-population ensemble DE with adaptive population contribution	All subpopulations use DE variants; lacks fundamentally different exploration mechanisms (e.g., heavy-tailed swarm behavior)	HALA combines a heavy-tailed swarm optimizer (enhanced ALA) with DE, achieving cross-paradigm synergy
	SaDE [28]	Self-adaptive control parameter setting (mutation strategy and *F*, CR)	Relies solely on DE mutation composition; no external exploration source outside the DE paradigm	HALA’s enhanced ALA provides external heavy-tailed exploration independent of DE operators
High-performing CEC algorithms	MVMO [29]	Mean-variance mapping optimization with memory mechanism	Model-based approach sensitive to initial sampling; not a population-based hybrid framework	HALA is a population-based hybrid method with explicit exploration–exploitation partitioning
	BI-population CMA-ES [30]	CMA-ES with bi-population scheme and active covariance update	Strong exploitation but limited exploration in non-separable multimodal functions; high per-evaluation cost	HALA’s ALA subpopulation provides persistent heavy-tailed global exploration to compensate for DE/SHADE’s conservative search

**Table 2:** Computational complexity comparison.

Algorithm	Total Computational Complexity
ALA	$O(N+T_{\max}\cdot N\cdot \left(D+1\right))$
SHADE	$O(N+T_{\max}\cdot N\cdot \left(D+1\right))$
L-SHADE	$O(N+T_{\max}\cdot N\cdot \left(D+1\right))$
HALA (proposed)	$O(N+T_{\max}\cdot N\cdot \left(D+1\right))$

**Table 3:** Overview of IEEE CEC2017 benchmark functions.

Type	No.	Function Name	Dimensions	Search Range	$f_{\min}$
Unimodal Functions	F1	Shifted and Rotated Bent Cigar Function	10, 30, 50, 100	[−100, 100]^*D*^	100
	F2	Shifted and Rotated Sum of Different Power Function	10, 30, 50, 100	[−100, 100]^*D*^	200
	F3	Shifted and Rotated Zakharov Function	10, 30, 50, 100	[−100, 100]^*D*^	300
Multimodal Functions	F4	Shifted and Rotated Rosenbrock’s Function	10, 30, 50, 100	[−100, 100]^*D*^	400
	F5	Shifted and Rotated Rastrigin’s Function	10, 30, 50, 100	[−100, 100]^*D*^	500
	F6	Shifted and Rotated Expanded Schaffer’s F6 Function	10, 30, 50, 100	[−100, 100]^*D*^	600
	F7	Shifted and Rotated Lunacek Bi-Rastrigin Function	10, 30, 50, 100	[−100, 100]^*D*^	700
	F8	Shifted and Rotated Non-Continuous Rastrigin’s Function	10, 30, 50, 100	[−100, 100]^*D*^	800
	F9	Shifted and Rotated Levy Function	10, 30, 50, 100	[−100, 100]^*D*^	900
	F10	Shifted and Rotated Schwefel’s Function	10, 30, 50, 100	[−100, 100]^*D*^	1000
Hybrid Functions	F11	Hybrid Function 1 (N=3)	10, 30, 50, 100	[−100, 100]^*D*^	1100
	F12	Hybrid Function 2 (N=3)	10, 30, 50, 100	[−100, 100]^*D*^	1200
	F13	Hybrid Function 3 (N=3)	10, 30, 50, 100	[−100, 100]^*D*^	1300
	F14	Hybrid Function 4 (N=4)	10, 30, 50, 100	[−100, 100]^*D*^	1400
	F15	Hybrid Function 5 (N=4)	10, 30, 50, 100	[−100, 100]^*D*^	1500
	F16	Hybrid Function 6 (N=4)	10, 30, 50, 100	[−100, 100]^*D*^	1600
	F17	Hybrid Function 7 (N=5)	10, 30, 50, 100	[−100, 100]^*D*^	1700
	F18	Hybrid Function 8 (N=5)	10, 30, 50, 100	[−100, 100]^*D*^	1800
	F19	Hybrid Function 9 (N=5)	10, 30, 50, 100	[−100, 100]^*D*^	1900
	F20	Hybrid Function 10 (N=6)	10, 30, 50, 100	[−100, 100]^*D*^	2000
Composition Functions	F21	Composition Function 1 (N=3)	10, 30, 50, 100	[−100, 100]^*D*^	2100
	F22	Composition Function 2 (N=3)	10, 30, 50, 100	[−100, 100]^*D*^	2200
	F23	Composition Function 3 (N=4)	10, 30, 50, 100	[−100, 100]^*D*^	2300
	F24	Composition Function 4 (N=4)	10, 30, 50, 100	[−100, 100]^*D*^	2400
	F25	Composition Function 5 (N=5)	10, 30, 50, 100	[−100, 100]^*D*^	2500
	F26	Composition Function 6 (N=5)	10, 30, 50, 100	[−100, 100]^*D*^	2600
	F27	Composition Function 7 (N=6)	10, 30, 50, 100	[−100, 100]^*D*^	2700
	F28	Composition Function 8 (N=6)	10, 30, 50, 100	[−100, 100]^*D*^	2800
	F29	Composition Function 9 (N=3)	10, 30, 50, 100	[−100, 100]^*D*^	2900
	F30	Composition Function 10 (N=3)	10, 30, 50, 100	[−100, 100]^*D*^	3000

Note: Function F2 is excluded due to numerical instability in high dimensions.

**Table 4:** Overview of IEEE CEC2022 benchmark functions.

Type	No.	Function Name	Dimensions	Search Range	$f_{\min}$
Unimodal Function	F1	Shifted Zakharov Function	10	[−100, 100]^*D*^	300
Multimodal Functions	F2	Shifted and Rotated Rosenbrock’s Function	10	[−100, 100]^*D*^	400
	F3	Shifted and Rotated Rastrigin’s Function	10	[−100, 100]^*D*^	600
	F4	Shifted and Rotated Non-Continuous Rastrigin’s Function	10	[−100, 100]^*D*^	800
	F5	Shifted Levy Function	10	[−100, 100]^*D*^	900
Hybrid Functions	F6	Hybrid Function 1 (N=3)	10	[−100, 100]^*D*^	1800
	F7	Hybrid Function 2 (N=4)	10	[−100, 100]^*D*^	2000
	F8	Hybrid Function 3 (N=5)	10	[−100, 100]^*D*^	2200
Composition Functions	F9	Composition Function 1 (N=3)	10	[−100, 100]^*D*^	2300
	F10	Composition Function 2 (N=4)	10	[−100, 100]^*D*^	2400
	F11	Composition Function 3 (N=5)	10	[−100, 100]^*D*^	2600
	F12	Composition Function 4 (N=6)	10	[−100, 100]^*D*^	2700

**Table 5:** Key parameter settings for each algorithm (associated with original paper notations).

Algorithm	Year	Parameter	Value
LSHADE [21]	2014	$M_{CR}$	0.5 (initial)
		$M_{F}$	0.5 (initial)
		Memory size *H*	5–10
		Archive rate Arc_rate	1.4–2.6
		$p_{\mathrm{best}}$ rate	0.11
		Population size	Linear reduction to 4
LSHADE-SPACMA [22]	2017	*p*	0.11
		Memory size *H*	1.4
		Archive rate Arc_rate	5
		FCP	0.5
		*c*	0.8
ALA [14]	2025	Energy decay θ	$2\arctan(1-t/T_{\max})$
		Migration probability	0.3
		Spiral foraging probability	0.5
		Direction *F*	±1 (randomly selected)
		Evasion decay *G*	$2\cdot \mathrm{sign}\left(\mathrm{rand}-0.5\right)\cdot \left(1-t/T_{\max}\right)$
AOOA [37]	2025	Growth factor	1.8 (1−t)
		Dynamic adjustment factor *c*	$1-{\left(t/T\right)}^{3}$
		Levy flight parameter β	1.5
		Probability of encountering obstacles	0.5
		Gravitational acceleration *g*	9.8
CCO [40]	2025	*a*	1.34
		*b*	0.3
		C,T,w	[1, 0]
		die	0.02
CQALA [17]	2025	*a*	0.5
		β	1.5
BAEO [38]	2025	Weight of control force *a*	0.3
		Controls the weight of the direction *b*	0.5
		Around the number of solutions num	10
		Proportion of individuals producing aerodynamic effects	0.2
BPBO [39]	2025	Finding factor $P_{i}$	0.7
CEO [41]	2025	Crossover control $C_{r}$	random number in [0, 1]
DFL [42]	2025	Dynamic parameter ω	$e^{-{\left(\frac{4t}{T}\right)}^{2}}$
DHOA [44]	2025	$C_{1}$	1
		μ	25
		K	0.5
		$C_{3}$	3
DMOA [43]	2025	$T_{d}$	$\frac{9}{10}\times T_{\max}$
		$k_{q}$	$\mathrm{randi}\left(\left\lceil \frac{\mathrm{Dim}}{8\times q}\right\rceil ,\max\left\{2,\left\lceil \frac{\mathrm{Dim}}{3\times q}\right\rceil \right\}\right),$
			q=1,2,3,4,5
		$k_{r}$	$\mathrm{randi}\left(2,\max\left\{2,\left\lceil \frac{\mathrm{Dim}}{3}\right\rceil \right\}\right)$
		*u*	0.9
FGO [45]	2025	*M*	0.6
		$E_{p}$	0.7
		*R*	0.9
KLA [46]	2025	Non-parametric	-
PGA [47]	2025	$C_{1}$	0.4
		$C_{2}$	0.6
SO [48]	2025	Scaling factor *k*	1
SOO [49]	2025	Scaling factor *S*	$2-2t/T_{max}$

**Table 6:** Mean runtime over 30 runs on CEC2017 (D=30). Ratio relative to L-SHADE-SPACMA (fastest) shown in parentheses.

Rank	Algorithm	Time (s)
1	L-SHADE-SPACMA	127.26 (1.00×)
2	L-SHADE	128.12 (1.01×)
3	BIHO	146.56 (1.15×)
4	PGA	152.06 (1.19×)
5	AOOA	153.14 (1.20×)
6	SOO	154.84 (1.22×)
7	FGO	155.74 (1.22×)
8	DMOA	155.97 (1.23×)
9	CEO	158.27 (1.24×)
10	CCO	158.50 (1.25×)
11	BTO	169.45 (1.33×)
12	ALA	179.49 (1.41×)
13	DHOA	192.97 (1.52×)
14	BPBO	221.24 (1.74×)
15	DFL	221.69 (1.74×)
16	HALA (proposed)	321.71 (2.53×)
17	SO	344.30 (2.71×)
18	CQALA	493.17 (3.87×)

Note: Experiments conducted on MATLAB R2020a with an Intel Core i9-9900K CPU @ 3.60 GHz (Intel, Santa Clara, CA, USA) and 32 GB RAM.

**Table 7:** Performance ranking (by best value) of HALA and other optimizers for pressure vessel design.

Algorithms	Optimal Variables				Optimal Cost
	$T_{s}$	$T_{h}$	R	L	
DFL	0.778169	0.384649	40.319619	200.000000	5885.3327736
CCO	0.778169	0.384649	40.319619	200.000000	5885.3327847
BPBO	0.778169	0.384649	40.319619	200.000000	5885.3328927
HALA	0.778169	0.384649	40.319620	199.999989	5885.3337409
ALA	0.778180	0.384655	40.320185	199.992117	5885.3514676
FGO	0.778171	0.384652	40.319697	200.000000	5885.3737241
SOO	0.778177	0.384649	40.319619	200.000000	5885.3908886
CQALA	0.781526	0.386309	40.493586	197.592336	5891.0967719
AOOA	0.785444	0.388259	40.695412	194.834213	5898.0811960
CEO	0.810106	0.400436	41.974394	178.179878	5942.1951570
BIHO	0.782224	0.395686	40.320734	200.000000	5946.8460214
PGA	0.804403	0.396441	41.361663	186.154124	5973.2178584
LSHADE	0.821792	0.411196	42.579888	170.760718	5980.2244761
LSHADE-SPACMA	0.869167	0.431302	45.034482	143.360591	6065.8143868
DHOA	0.856403	0.436449	44.293588	151.333939	6091.4194772
SO	0.847954	0.489932	43.826083	156.423229	6272.5931074
BTO	0.954948	0.509816	47.988297	116.323671	6609.4745571
DMOA	0.930260	0.635065	47.318963	131.103198	7291.9168917

**Table 8:** Performance ranking (by best value) of HALA and other optimizers for rolling element bearing design.

Algorithm	Optimal Variables										Optimal Load- Carrying Capacity
	$D_{m}$	$D_{b}$	Z	$f_{i}$	$f_{o}$	$K_{D_{\min}}$	$K_{D_{\max}}$	ε	e	ζ	
HALA	125.719056	21.425590	11	0.515	0.515	0.400000	0.646653	0.300000	0.100000	0.612824	74,647.473709
ALA	125.719056	21.425590	11	0.515	0.515	0.500000	0.700000	0.300000	0.020000	0.600000	74,647.473709
BPBO	125.719056	21.425590	11	0.515	0.515	0.477238	0.698469	0.300000	0.020000	0.617474	74,647.473709
CCO	125.719056	21.425590	11	0.515	0.515	0.400425	0.700000	0.300000	0.020000	0.665444	74,647.473709
CEO	125.719056	21.425590	11	0.515	0.515	0.479549	0.680838	0.300000	0.100000	0.666685	74,647.473709
DFL	125.719056	21.425590	11	0.515	0.515	0.400364	0.612918	0.300000	0.020006	0.660013	74,647.473709
SOO	125.719056	21.425590	11	0.515	0.515	0.500000	0.693605	0.300000	0.020000	0.600278	74,647.473709
CQALA	125.719056	21.425590	11	0.515	0.515	0.408363	0.656560	0.300000	0.098968	0.643416	74,647.473709
FGO	125.719027	21.425580	11	0.515	0.515	0.500000	0.700000	0.300000	0.100000	0.600000	74,647.409063
LSHADE	125.718986	21.425575	11	0.515	0.515	0.470003	0.617113	0.300000	0.063333	0.623861	74,647.367557
BIHO	125.717400	21.425210	11	0.515	0.515	0.400000	0.700000	0.300000	0.100000	0.600000	74,645.079929
LSHADE- SPACMA	125.709794	21.423619	11	0.515	0.515	0.445453	0.627880	0.300274	0.057904	0.645524	74,635.069396
PGA	125.704367	21.421913	11	0.515	0.515	0.402671	0.673335	0.300000	0.067184	0.610117	74,624.281705
DHOA	125.710325	21.404291	11	0.515	0.515	0.426424	0.674268	0.300000	0.088325	0.616966	74,511.731344
AOOA	125.597251	21.399705	11	0.515	0.515	0.427506	0.642383	0.303493	0.090759	0.684965	74,484.613841
DMOA	125.569265	21.374784	11	0.515	0.515	0.461546	0.643156	0.303504	0.049204	0.641043	74,326.323172
BTO	125.483664	21.365232	11	0.515	0.515	0.434608	0.655701	0.306874	0.083273	0.686370	74,267.112384
SO	125.151683	21.281194	11	0.515	0.515	0.403709	0.614356	0.301299	0.052380	0.600000	73,739.010186

**Table 9:** Performance ranking (by best value) of HALA and other optimizers for tension/compression spring design.

Algorithm	Optimal Variables			Optimal Weight
	d	D	N	
HALA	0.051897	0.361749	11	0.012666021011
CQALA	0.051897	0.361749	11	0.012666021011
SOO	0.051897	0.361749	11	0.012666021011
CCO	0.051897	0.361749	11	0.012666021011
ALA	0.051897	0.361749	11	0.012666021147
AOOA	0.051897	0.361749	11	0.012666044284
LSHADE	0.051898	0.361764	11	0.012666895698
DFL	0.051899	0.361793	11	0.012668558828
CEO	0.051204	0.345163	12	0.012669560575
BPBO	0.051204	0.345163	12	0.012669560575
PGA	0.051207	0.345194	12	0.012672002580
LSHADE-SPACMA	0.051208	0.345223	12	0.012673727702
FGO	0.051914	0.361910	11	0.012679855597
DHOA	0.051934	0.362240	11	0.012701242811
BTO	0.051231	0.345767	12	0.012704859881
BIHO	0.054019	0.408030	9	0.013097299127
SO	0.057190	0.503951	6	0.013185941139
DMOA	0.050000	0.310563	15	0.013198908910

**Table 10:** Performance ranking (by best value) of HALA and other optimizers for cantilever beam design.

Algorithm	Optimal Variables					Optimal Weight
	$x_{1}$	$x_{2}$	$x_{3}$	$x_{4}$	$x_{5}$	
DFL	6.017244	5.308463	4.495958	3.500123	2.151875	1.3399565996
HALA	6.017833	5.311599	4.493109	3.499130	2.151996	1.3399568320
LSHADE	6.011371	5.306611	4.498195	3.505520	2.151989	1.3399579874
ALA	6.009449	5.305907	4.498970	3.502996	2.156381	1.3399589854
LSHADE-SPACMA	6.012420	5.314172	4.490105	3.509064	2.147976	1.3399610793
SOO	5.981849	5.322415	4.477215	3.528706	2.164613	1.3400273880
CCO	5.995566	5.310921	4.537781	3.477441	2.153252	1.3400375983
DHOA	6.006463	5.288121	4.526389	3.496235	2.158039	1.3400553985
CQALA	5.957324	5.363766	4.500843	3.541497	2.114787	1.3402407554
BTO	6.066591	5.257417	4.508512	3.558397	2.091009	1.3404721704
CEO	6.041247	5.216857	4.570704	3.453575	2.202472	1.3406549401
BPBO	5.928192	5.262153	4.438218	3.604986	2.260879	1.3412522763
AOOA	5.816943	5.299115	4.565581	3.654499	2.166332	1.3417541786
FGO	6.092750	5.169074	4.453270	3.490466	2.298418	1.3418481923
PGA	5.936602	5.333360	4.534137	3.657770	2.042436	1.3418686893
BIHO	5.644844	5.322328	4.731031	3.467785	2.465177	1.3497846859
DMOA	5.653130	5.293245	4.701902	3.660259	2.383707	1.3535959613
SO	5.001285	6.248595	4.946278	4.085011	2.153126	1.3999000308

**Table 11:** Performance ranking (by best value) of HALA and other optimizers for gear train design.

Algorithm	Optimal Variables				Optimal Cost
	$T_{a}$	$T_{b}$	$T_{d}$	$T_{f}$	
HALA	43	19	16	49	2.7008571489 $\times \;10^{-12}$
ALA	49	16	19	43	2.7008571489 $\times \;10^{-12}$
LSHADE	49	16	19	43	2.7008571489 $\times \;10^{-12}$
LSHADE-SPACMA	49	16	19	43	2.7008571489 $\times \;10^{-12}$
BIHO	49	19	16	43	2.7008571489 $\times \;10^{-12}$
BTO	43	16	19	49	2.7008571489 $\times \;10^{-12}$
BPBO	49	19	16	43	2.7008571489 $\times \;10^{-12}$
CCO	43	16	19	49	2.7008571489 $\times \;10^{-12}$
CQALA	49	19	16	43	2.7008571489 $\times \;10^{-12}$
DMOA	49	16	19	43	2.7008571489 $\times \;10^{-12}$
DHOA	43	19	16	49	2.7008571489 $\times \;10^{-12}$
FGO	49	16	19	43	2.7008571489 $\times \;10^{-12}$
PGA	49	16	19	43	2.7008571489 $\times \;10^{-12}$
AOOA	53	15	26	51	2.3078157333 $\times \;10^{-11}$
DFL	51	13	30	53	2.3078157333 $\times \;10^{-11}$
SO	51	15	26	53	2.3078157333 $\times \;10^{-11}$
SOO	34	20	13	53	2.3078157333 $\times \;10^{-11}$
CEO	56	13	23	37	6.6020898802 $\times \;10^{-10}$

## Data Availability

All data generated or analyzed during this study are included in this published article. The source code of HALA is released at https://github.com/SylvanHuang/HALA (accessed on 27 June 2026) upon publication.

## Appendix A. Numerical Optimization Experiment Results

**Table A1:** Comparison of HALA and other algorithms on the CEC2017 benchmark suite (Dim = 10) (excluding F2)—Part 1: HALA vs. ALA, LSHADE, LSHADE-SPACMA, AOOA, BAEO, BPBO, CCO, CEO.

Function	Metric	HALA	ALA	LSHADE	LSHADE- SPACMA	AOOA	BAEO	BPBO	CCO	CEO
F1	Mean	$3.03\times 10^{3}$	$9.49\times 10^{3}$	$6.93\times 10^{4}$	$7.59\times 10^{4}$	$5.48\times 10^{6}$	$1.29\times 10^{10}$	$8.87\times 10^{3}$	$8.58\times 10^{3}$	$5.20\times 10^{5}$
	Std	$4.62\times 10^{3}$	$9.12\times 10^{3}$	$1.67\times 10^{5}$	$1.61\times 10^{5}$	$5.15\times 10^{6}$	$2.92\times 10^{9}$	$2.17\times 10^{4}$	$8.27\times 10^{3}$	$8.41\times 10^{5}$
	*p*-value	–	0.0018	0.0011	0.0012	<$10^{-11}$	<$10^{-11}$	0.0316	0.0007	<$10^{-11}$
	Rank	2	6	7	8	13	17	5	4	10
F3	Mean	$3.02\times 10^{2}$	$3.00\times 10^{2}$	$4.28\times 10^{3}$	$5.66\times 10^{3}$	$9.71\times 10^{2}$	$3.26\times 10^{4}$	$3.54\times 10^{2}$	$3.05\times 10^{2}$	$3.02\times 10^{2}$
	Std	$4.77\times 10^{0}$	$3.96\times 10^{-1}$	$2.66\times 10^{3}$	$2.30\times 10^{3}$	$5.83\times 10^{2}$	$7.44\times 10^{3}$	$7.24\times 10^{1}$	$1.50\times 10^{1}$	$1.60\times 10^{0}$
	*p*-value	–	0.0021	<$10^{-11}$	<$10^{-11}$	<$10^{-11}$	<$10^{-11}$	<$10^{-11}$	0.0571	0.5999
	Rank	2	1	13	14	10	17	7	4	2
F4	Mean	$4.06\times 10^{2}$	$4.06\times 10^{2}$	$4.10\times 10^{2}$	$4.08\times 10^{2}$	$4.18\times 10^{2}$	$1.60\times 10^{3}$	$4.08\times 10^{2}$	$4.07\times 10^{2}$	$4.11\times 10^{2}$
	Std	$2.20\times 10^{0}$	$2.74\times 10^{0}$	$6.51\times 10^{0}$	$1.92\times 10^{0}$	$2.63\times 10^{1}$	$5.12\times 10^{2}$	$1.39\times 10^{0}$	$2.68\times 10^{0}$	$1.54\times 10^{1}$
	*p*-value	–	0.7813	<$10^{-11}$	0.0001	0.0001	<$10^{-11}$	<$10^{-11}$	0.0545	0.0082
	Rank	1	1	8	5	15	17	5	3	9
F5	Mean	$5.14\times 10^{2}$	$5.19\times 10^{2}$	$5.09\times 10^{2}$	$5.09\times 10^{2}$	$5.30\times 10^{2}$	$5.94\times 10^{2}$	$5.21\times 10^{2}$	$5.46\times 10^{2}$	$5.35\times 10^{2}$
	Std	$4.72\times 10^{0}$	$7.91\times 10^{0}$	$3.20\times 10^{0}$	$3.24\times 10^{0}$	$1.22\times 10^{1}$	$1.23\times 10^{1}$	$6.66\times 10^{0}$	$1.40\times 10^{1}$	$8.73\times 10^{0}$
	*p*-value	–	0.0024	0.0002	0.0006	<$10^{-11}$	<$10^{-11}$	0.0006	<$10^{-11}$	<$10^{-11}$
	Rank	4	6	1	1	10	18	8	15	12
F6	Mean	$6.00\times 10^{2}$	$6.00\times 10^{2}$	$6.01\times 10^{2}$	$6.00\times 10^{2}$	$6.20\times 10^{2}$	$6.69\times 10^{2}$	$6.05\times 10^{2}$	$6.25\times 10^{2}$	$6.32\times 10^{2}$
	Std	$7.09\times 10^{-1}$	$5.22\times 10^{-1}$	$6.56\times 10^{-1}$	$3.34\times 10^{-1}$	$8.08\times 10^{0}$	$8.94\times 10^{0}$	$2.55\times 10^{0}$	$1.17\times 10^{1}$	$5.61\times 10^{0}$
	*p*-value	–	0.2623	0.0230	0.7813	<$10^{-11}$	<$10^{-11}$	<$10^{-11}$	<$10^{-11}$	<$10^{-11}$
	Rank	1	1	4	1	13	18	6	14	16
F7	Mean	$7.21\times 10^{2}$	$7.27\times 10^{2}$	$7.17\times 10^{2}$	$7.16\times 10^{2}$	$7.65\times 10^{2}$	$1.19\times 10^{3}$	$7.28\times 10^{2}$	$8.06\times 10^{2}$	$8.12\times 10^{2}$
	Std	$6.17\times 10^{0}$	$8.39\times 10^{0}$	$2.86\times 10^{0}$	$2.98\times 10^{0}$	$2.44\times 10^{1}$	$1.22\times 10^{2}$	$1.06\times 10^{1}$	$5.01\times 10^{1}$	$3.23\times 10^{1}$
	*p*-value	–	0.0041	0.0117	0.0005	<$10^{-11}$	<$10^{-11}$	0.0018	<$10^{-11}$	<$10^{-11}$
	Rank	3	5	2	1	12	18	6	14	16
F8	Mean	$8.15\times 10^{2}$	$8.15\times 10^{2}$	$8.10\times 10^{2}$	$8.10\times 10^{2}$	$8.37\times 10^{2}$	$9.36\times 10^{2}$	$8.19\times 10^{2}$	$8.48\times 10^{2}$	$8.50\times 10^{2}$
	Std	$5.79\times 10^{0}$	$5.92\times 10^{0}$	$6.01\times 10^{0}$	$3.39\times 10^{0}$	$1.72\times 10^{1}$	$1.43\times 10^{1}$	$8.10\times 10^{0}$	$2.22\times 10^{1}$	$1.20\times 10^{1}$
	*p*-value	–	0.5857	0.0057	0.0030	<$10^{-11}$	<$10^{-11}$	0.0545	<$10^{-11}$	<$10^{-11}$
	Rank	3	3	1	1	10	18	5	14	15
F9	Mean	$9.12\times 10^{2}$	$9.04\times 10^{2}$	$9.05\times 10^{2}$	$9.06\times 10^{2}$	$1.20\times 10^{3}$	$2.42\times 10^{3}$	$9.26\times 10^{2}$	$1.53\times 10^{3}$	$2.02\times 10^{3}$
	Std	$1.53\times 10^{1}$	$1.24\times 10^{1}$	$1.12\times 10^{1}$	$1.31\times 10^{1}$	$3.12\times 10^{302}$	$3.84\times 10^{302}$	$2.61\times 10^{1}$	$3.22\times 10^{302}$	$5.74\times 10^{302}$
	*p*-value	–	0.0032	0.0017	0.0093	<$10^{-11}$	<$10^{-11}$	0.0053	<$10^{-11}$	<$10^{-11}$
	Rank	4	1	2	3	13	17	6	15	16
F10	Mean	$1.69\times 10^{3}$	$1.86\times 10^{3}$	$1.69\times 10^{3}$	$1.71\times 10^{3}$	$2.01\times 10^{3}$	$2.50\times 10^{3}$	$1.67\times 10^{3}$	$2.07\times 10^{3}$	$2.24\times 10^{3}$
	Std	$2.39\times 10^{2}$	$2.84\times 10^{2}$	$1.86\times 10^{2}$	$2.01\times 10^{2}$	$3.10\times 10^{2}$	$1.40\times 10^{2}$	$2.34\times 10^{2}$	$3.11\times 10^{2}$	$3.58\times 10^{302}$
	*p*-value	–	0.0093	0.7189	0.7971	0.0002	<$10^{-11}$	0.9426	0.0001	<$10^{-11}$
	Rank	3	7	3	5	10	17	2	11	13
F11	Mean	$1.12\times 10^{3}$	$1.12\times 10^{3}$	$1.12\times 10^{3}$	$1.12\times 10^{3}$	$1.17\times 10^{3}$	$5.32\times 10^{3}$	$1.12\times 10^{3}$	$1.21\times 10^{3}$	$1.17\times 10^{3}$
	Std	$1.42\times 10^{1}$	$6.98\times 10^{0}$	$1.28\times 10^{1}$	$1.77\times 10^{1}$	$3.47\times 10^{1}$	$2.73\times 10^{3}$	$7.23\times 10^{0}$	$7.21\times 10^{1}$	$3.02\times 10^{1}$
	*p*-value	–	0.0196	0.1064	0.5716	<$10^{-11}$	<$10^{-11}$	0.1020	<$10^{-11}$	<$10^{-11}$
	Rank	1	1	1	1	10	17	1	15	10
F12	Mean	$8.81\times 10^{4}$	$1.94\times 10^{4}$	$5.71\times 10^{5}$	$1.07\times 10^{6}$	$6.99\times 10^{6}$	$2.40\times 10^{9}$	$2.17\times 10^{4}$	$4.41\times 10^{6}$	$5.16\times 10^{6}$
	Std	$3.35\times 10^{5}$	$1.26\times 10^{4}$	$8.34\times 10^{5}$	$2.50\times 10^{6}$	$7.50\times 10^{6}$	$1.23\times 10^{9}$	$3.08\times 10^{4}$	$4.00\times 10^{6}$	$2.99\times 10^{6}$
	*p*-value	–	0.7189	0.0002	<$10^{-11}$	<$10^{-11}$	<$10^{-11}$	0.3286	<$10^{-11}$	<$10^{-11}$
	Rank	3	1	4	5	14	17	2	10	12
F13	Mean	$1.43\times 10^{3}$	$1.86\times 10^{3}$	$3.92\times 10^{3}$	$3.40\times 10^{3}$	$9.30\times 10^{3}$	$2.21\times 10^{8}$	$2.16\times 10^{3}$	$1.30\times 10^{4}$	$1.30\times 10^{4}$
	Std	$1.21\times 10^{2}$	$2.49\times 10^{3}$	$3.04\times 10^{3}$	$2.75\times 10^{3}$	$8.76\times 10^{3}$	$1.43\times 10^{8}$	$5.58\times 10^{2}$	$1.13\times 10^{4}$	$9.22\times 10^{3}$
	*p*-value	–	0.1470	<$10^{-11}$	<$10^{-11}$	<$10^{-11}$	<$10^{-11}$	<$10^{-11}$	<$10^{-11}$	<$10^{-11}$
	Rank	1	2	5	4	7	17	3	11	11
F14	Mean	$1.43\times 10^{3}$	$1.43\times 10^{3}$	$1.46\times 10^{3}$	$1.52\times 10^{3}$	$1.69\times 10^{3}$	$1.91\times 10^{7}$	$1.48\times 10^{3}$	$2.18\times 10^{3}$	$1.58\times 10^{3}$
	Std	$1.32\times 10^{1}$	$4.37\times 10^{0}$	$1.86\times 10^{2}$	$4.76\times 10^{2}$	$3.40\times 10^{2}$	$6.22\times 10^{7}$	$3.44\times 10^{1}$	$1.18\times 10^{3}$	$7.98\times 10^{1}$
	*p*-value	–	0.8451	0.8936	0.4528	<$10^{-11}$	<$10^{-11}$	<$10^{-11}$	<$10^{-11}$	<$10^{-11}$
	Rank	1	1	3	5	9	18	4	14	6
F15	Mean	$1.52\times 10^{3}$	$1.52\times 10^{3}$	$1.57\times 10^{3}$	$1.54\times 10^{3}$	$4.83\times 10^{3}$	$9.03\times 10^{6}$	$1.68\times 10^{3}$	$6.77\times 10^{3}$	$5.64\times 10^{3}$
	Std	$1.06\times 10^{1}$	$1.57\times 10^{1}$	$1.06\times 10^{2}$	$7.60\times 10^{1}$	$3.64\times 10^{3}$	$2.09\times 10^{7}$	$9.07\times 10^{1}$	$5.40\times 10^{3}$	$1.71\times 10^{3}$
	*p*-value	–	0.4779	0.0008	0.0897	<$10^{-11}$	<$10^{-11}$	<$10^{-11}$	<$10^{-11}$	<$10^{-11}$
	Rank	1	1	4	3	8	17	5	14	12
F16	Mean	$1.64\times 10^{3}$	$1.63\times 10^{3}$	$1.66\times 10^{3}$	$1.65\times 10^{3}$	$1.79\times 10^{3}$	$2.49\times 10^{3}$	$1.65\times 10^{3}$	$1.83\times 10^{3}$	$2.03\times 10^{3}$
	Std	$5.51\times 10^{1}$	$4.60\times 10^{1}$	$6.57\times 10^{1}$	$5.75\times 10^{1}$	$1.16\times 10^{2}$	$1.49\times 10^{2}$	$5.47\times 10^{1}$	$1.43\times 10^{2}$	$1.73\times 10^{2}$
	*p*-value	–	0.9099	0.2452	0.7971	<$10^{-11}$	<$10^{-11}$	0.2289	<$10^{-11}$	<$10^{-11}$
	Rank	2	1	5	3	10	18	3	12	16
F17	Mean	$1.73\times 10^{3}$	$1.74\times 10^{3}$	$1.73\times 10^{3}$	$1.73\times 10^{3}$	$1.80\times 10^{3}$	$2.20\times 10^{3}$	$1.75\times 10^{3}$	$1.81\times 10^{3}$	$1.96\times 10^{3}$
	Std	$1.55\times 10^{1}$	$1.20\times 10^{1}$	$1.02\times 10^{1}$	$9.91\times 10^{0}$	$5.58\times 10^{1}$	$1.30\times 10^{2}$	$1.88\times 10^{1}$	$6.01\times 10^{1}$	$1.11\times 10^{2}$
	*p*-value	–	0.0026	0.2802	0.8774	<$10^{-11}$	<$10^{-11}$	0.0001	<$10^{-11}$	<$10^{-11}$
	Rank	1	4	1	1	10	18	5	12	17
F18	Mean	$1.86\times 10^{3}$	$1.87\times 10^{3}$	$4.72\times 10^{3}$	$3.77\times 10^{3}$	$2.23\times 10^{4}$	$7.97\times 10^{8}$	$3.09\times 10^{3}$	$1.75\times 10^{4}$	$1.42\times 10^{4}$
	Std	$3.61\times 10^{1}$	$3.19\times 10^{1}$	$3.00\times 10^{3}$	$2.84\times 10^{3}$	$1.54\times 10^{4}$	$7.92\times 10^{8}$	$1.52\times 10^{3}$	$1.13\times 10^{4}$	$1.24\times 10^{4}$
	*p*-value	–	0.1204	<$10^{-11}$	<$10^{-11}$	<$10^{-11}$	<$10^{-11}$	<$10^{-11}$	<$10^{-11}$	<$10^{-11}$
	Rank	1	2	5	4	13	17	3	11	9
F19	Mean	$1.91\times 10^{3}$	$1.91\times 10^{3}$	$1.94\times 10^{3}$	$1.95\times 10^{3}$	$4.83\times 10^{3}$	$9.48\times 10^{7}$	$1.96\times 10^{3}$	$8.23\times 10^{3}$	$6.13\times 10^{3}$
	Std	$6.30\times 10^{0}$	$2.66\times 10^{0}$	$1.63\times 10^{2}$	$1.44\times 10^{2}$	$3.75\times 10^{3}$	$1.86\times 10^{8}$	$3.65\times 10^{1}$	$8.38\times 10^{3}$	$7.79\times 10^{3}$
	*p*-value	–	0.6583	0.4779	0.7343	<$10^{-11}$	<$10^{-11}$	<$10^{-11}$	<$10^{-11}$	<$10^{-11}$
	Rank	1	1	3	4	9	17	5	15	12
F20	Mean	$2.02\times 10^{3}$	$2.05\times 10^{3}$	$2.01\times 10^{3}$	$2.02\times 10^{3}$	$2.11\times 10^{3}$	$2.32\times 10^{3}$	$2.06\times 10^{3}$	$2.11\times 10^{3}$	$2.30\times 10^{3}$
	Std	$2.28\times 10^{1}$	$4.00\times 10^{1}$	$1.21\times 10^{1}$	$2.37\times 10^{1}$	$5.49\times 10^{1}$	$6.78\times 10^{1}$	$2.34\times 10^{1}$	$5.99\times 10^{1}$	$1.07\times 10^{2}$
	*p*-value	–	<$10^{-11}$	0.5170	0.6143	<$10^{-11}$	<$10^{-11}$	<$10^{-11}$	<$10^{-11}$	<$10^{-11}$
	Rank	2	5	1	2	10	18	6	10	17
F21	Mean	$2.25\times 10^{3}$	$2.25\times 10^{3}$	$2.28\times 10^{3}$	$2.28\times 10^{3}$	$2.23\times 10^{3}$	$2.42\times 10^{3}$	$2.20\times 10^{3}$	$2.26\times 10^{3}$	$2.40\times 10^{3}$
	Std	$6.41\times 10^{1}$	$5.74\times 10^{1}$	$4.54\times 10^{1}$	$4.06\times 10^{1}$	$4.46\times 10^{1}$	$3.60\times 10^{1}$	$1.75\times 10^{1}$	$6.67\times 10^{1}$	$4.20\times 10^{1}$
	*p*-value	–	0.3493	0.1204	0.0256	0.6435	<$10^{-11}$	0.0053	0.1414	<$10^{-11}$
	Rank	6	6	9	9	3	18	1	8	17
F22	Mean	$2.30\times 10^{3}$	$2.30\times 10^{3}$	$2.30\times 10^{3}$	$2.30\times 10^{3}$	$2.31\times 10^{3}$	$3.88\times 10^{3}$	$2.27\times 10^{3}$	$2.32\times 10^{3}$	$2.31\times 10^{3}$
	Std	$1.69\times 10^{0}$	$1.29\times 10^{1}$	$2.33\times 10^{0}$	$1.02\times 10^{1}$	$1.60\times 10^{1}$	$2.41\times 10^{2}$	$3.25\times 10^{1}$	$2.61\times 10^{1}$	$7.11\times 10^{0}$
	*p*-value	–	0.0011	0.0627	0.3086	0.0004	<$10^{-11}$	0.0003	0.0005	0.0001
	Rank	2	2	2	2	7	17	1	10	7
F23	Mean	$2.62\times 10^{3}$	$2.62\times 10^{3}$	$2.61\times 10^{3}$	$2.61\times 10^{3}$	$2.64\times 10^{3}$	$2.85\times 10^{3}$	$2.62\times 10^{3}$	$2.64\times 10^{3}$	$2.70\times 10^{3}$
	Std	$8.40\times 10^{0}$	$1.13\times 10^{1}$	$3.56\times 10^{0}$	$5.23\times 10^{0}$	$1.46\times 10^{1}$	$6.27\times 10^{1}$	$7.82\times 10^{0}$	$1.59\times 10^{1}$	$5.34\times 10^{1}$
	*p*-value	–	0.0140	0.0218	0.0786	<$10^{-11}$	<$10^{-11}$	0.0333	<$10^{-11}$	<$10^{-11}$
	Rank	3	3	1	1	11	18	3	11	16
F24	Mean	$2.71\times 10^{3}$	$2.74\times 10^{3}$	$2.70\times 10^{3}$	$2.71\times 10^{3}$	$2.74\times 10^{3}$	$3.02\times 10^{3}$	$2.55\times 10^{3}$	$2.74\times 10^{3}$	$2.85\times 10^{3}$
	Std	$8.41\times 10^{1}$	$4.67\times 10^{1}$	$7.76\times 10^{1}$	$6.50\times 10^{1}$	$8.92\times 10^{1}$	$8.94\times 10^{1}$	$9.31\times 10^{1}$	$1.05\times 10^{2}$	$1.10\times 10^{2}$
	*p*-value	–	0.0002	0.1915	0.0937	0.0060	<$10^{-11}$	0.0001	0.0656	0.0002
	Rank	5	9	4	5	9	18	1	9	16
F25	Mean	$2.92\times 10^{3}$	$2.93\times 10^{3}$	$2.93\times 10^{3}$	$2.93\times 10^{3}$	$2.95\times 10^{3}$	$4.59\times 10^{3}$	$2.91\times 10^{3}$	$2.93\times 10^{3}$	$2.93\times 10^{3}$
	Std	$2.29\times 10^{1}$	$2.37\times 10^{1}$	$1.84\times 10^{1}$	$1.92\times 10^{1}$	$2.39\times 10^{1}$	$6.96\times 10^{2}$	$2.09\times 10^{1}$	$6.87\times 10^{1}$	$2.30\times 10^{1}$
	*p*-value	–	0.6583	0.1529	0.0897	0.0001	<$10^{-11}$	0.3933	0.0978	0.0937
	Rank	2	3	3	3	13	18	1	3	3
F26	Mean	$2.96\times 10^{3}$	$3.10\times 10^{3}$	$2.96\times 10^{3}$	$2.99\times 10^{3}$	$3.02\times 10^{3}$	$4.62\times 10^{3}$	$2.84\times 10^{3}$	$3.12\times 10^{3}$	$3.60\times 10^{3}$
	Std	$1.67\times 10^{2}$	$4.04\times 10^{2}$	$4.85\times 10^{1}$	$6.45\times 10^{1}$	$8.37\times 10^{1}$	$3.46\times 10^{2}$	$1.37\times 10^{2}$	$1.81\times 10^{2}$	$6.94\times 10^{2}$
	*p*-value	–	0.3933	0.1020	0.0026	0.0012	<$10^{-11}$	0.0243	0.0006	0.0005
	Rank	4	10	4	7	8	17	1	11	16
F27	Mean	$3.09\times 10^{3}$	$3.09\times 10^{3}$	$3.10\times 10^{3}$	$3.10\times 10^{3}$	$3.10\times 10^{3}$	$3.44\times 10^{3}$	$3.10\times 10^{3}$	$3.10\times 10^{3}$	$3.16\times 10^{3}$
	Std	$2.92\times 10^{0}$	$2.48\times 10^{0}$	$3.73\times 10^{0}$	$2.83\times 10^{0}$	$1.19\times 10^{1}$	$9.48\times 10^{1}$	$3.18\times 10^{0}$	$1.39\times 10^{1}$	$4.67\times 10^{1}$
	*p*-value	–	0.0978	0.0057	0.0034	<$10^{-11}$	<$10^{-11}$	0.0087	<$10^{-11}$	<$10^{-11}$
	Rank	1	1	3	3	3	18	3	3	15
F28	Mean	$3.30\times 10^{3}$	$3.30\times 10^{3}$	$3.30\times 10^{3}$	$3.29\times 10^{3}$	$3.31\times 10^{3}$	$4.18\times 10^{3}$	$3.13\times 10^{3}$	$3.32\times 10^{3}$	$3.39\times 10^{3}$
	Std	$1.60\times 10^{2}$	$1.84\times 10^{2}$	$1.21\times 10^{2}$	$1.18\times 10^{2}$	$9.72\times 10^{1}$	$1.83\times 10^{2}$	$8.54\times 10^{1}$	$1.43\times 10^{2}$	$5.74\times 10^{1}$
	*p*-value	–	0.9250	0.3709	0.8451	0.9099	<$10^{-11}$	0.0001	0.4822	0.0044
	Rank	7	7	7	6	10	18	1	11	14
F29	Mean	$3.18\times 10^{3}$	$3.19\times 10^{3}$	$3.19\times 10^{3}$	$3.19\times 10^{3}$	$3.26\times 10^{3}$	$3.72\times 10^{3}$	$3.19\times 10^{3}$	$3.27\times 10^{3}$	$3.38\times 10^{3}$
	Std	$2.93\times 10^{1}$	$5.07\times 10^{1}$	$2.19\times 10^{1}$	$2.49\times 10^{1}$	$6.75\times 10^{1}$	$1.60\times 10^{2}$	$2.42\times 10^{1}$	$6.86\times 10^{1}$	$1.15\times 10^{2}$
	*p*-value	–	0.6583	0.0270	0.0656	<$10^{-11}$	<$10^{-11}$	0.0519	<$10^{-11}$	<$10^{-11}$
	Rank	1	2	2	2	9	18	2	10	16
F30	Mean	$2.73\times 10^{5}$	$4.77\times 10^{5}$	$5.00\times 10^{5}$	$2.46\times 10^{5}$	$1.19\times 10^{6}$	$1.16\times 10^{8}$	$1.02\times 10^{4}$	$8.06\times 10^{5}$	$2.30\times 10^{5}$
	Std	$5.13\times 10^{5}$	$5.90\times 10^{5}$	$8.82\times 10^{5}$	$4.54\times 10^{5}$	$1.11\times 10^{6}$	$7.52\times 10^{7}$	$8.62\times 10^{3}$	$1.03\times 10^{6}$	$5.09\times 10^{5}$
	*p*-value	–	0.1064	0.0073	0.1109	0.0010	<$10^{-11}$	0.1714	0.0030	0.1414
	Rank	6	9	10	4	15	18	1	13	3
+/≈/−		–	21/5/3	15/6/8	17/7/5	29/0/0	29/0/0	22/2/5	29/0/0	28/0/1
Mean rank		2.55	3.52	4.07	3.90	10.14	17.55	3.52	10.59	12.21
Final ranking		1	2	5	4	11	18	3	12	15

**Table A2:** Comparison of HALA and other algorithms on the CEC2017 benchmark suite (Dim = 10) (excluding F2)—Part 2: HALA vs. CQALA, DFL, DMOA, DHOA, FGO, KLA, PGA, SO, SOO (continued from Table A1).

Function	Metric	CQALA	DFL	DMOA	DHOA	FGO	KLA	PGA	SO	SOO
F1	Mean	$2.48\times 10^{3}$	$1.65\times 10^{6}$	$8.04\times 10^{7}$	$1.56\times 10^{6}$	$1.31\times 10^{8}$	$1.40\times 10^{10}$	$3.51\times 10^{5}$	$4.77\times 10^{8}$	$7.54\times 10^{3}$
	Std	$2.89\times 10^{3}$	$5.86\times 10^{6}$	$2.01\times 10^{7}$	$1.76\times 10^{6}$	$1.02\times 10^{8}$	$4.90\times 10^{9}$	$2.79\times 10^{5}$	$2.90\times 10^{8}$	$1.01\times 10^{4}$
	*p*-value	0.8612	0.0001	<$10^{-11}$	<$10^{-11}$	<$10^{-11}$	<$10^{-11}$	<$10^{-11}$	<$10^{-11}$	0.0036
	Rank	1	12	14	11	15	18	9	16	3
F3	Mean	$4.68\times 10^{2}$	$3.09\times 10^{2}$	$2.70\times 10^{3}$	$5.54\times 10^{2}$	$1.44\times 10^{3}$	$6.52\times 10^{4}$	$3.09\times 10^{2}$	$1.31\times 10^{4}$	$5.76\times 10^{3}$
	Std	$8.88\times 10^{2}$	$1.11\times 10^{1}$	$1.01\times 10^{3}$	$3.39\times 10^{2}$	$2.88\times 10^{2}$	$2.61\times 10^{4}$	$1.72\times 10^{1}$	$4.94\times 10^{3}$	$5.78\times 10^{3}$
	*p*-value	0.9263	0.0157	<$10^{-11}$	<$10^{-11}$	<$10^{-11}$	<$10^{-11}$	0.0077	<$10^{-11}$	<$10^{-11}$
	Rank	8	5	12	9	11	18	5	16	15
F4	Mean	$4.07\times 10^{2}$	$4.13\times 10^{2}$	$4.11\times 10^{2}$	$4.11\times 10^{2}$	$4.15\times 10^{2}$	$1.90\times 10^{3}$	$4.17\times 10^{2}$	$4.99\times 10^{2}$	$4.09\times 10^{2}$
	Std	$2.86\times 10^{0}$	$1.94\times 10^{1}$	$1.23\times 10^{0}$	$1.24\times 10^{1}$	$7.80\times 10^{0}$	$6.17\times 10^{2}$	$2.33\times 10^{1}$	$5.16\times 10^{1}$	$1.23\times 10^{1}$
	*p*-value	0.0978	0.0015	<$10^{-11}$	0.0011	<$10^{-11}$	<$10^{-11}$	0.0006	<$10^{-11}$	0.0243
	Rank	3	12	9	9	13	18	14	16	7
F5	Mean	$5.17\times 10^{2}$	$5.37\times 10^{2}$	$5.33\times 10^{2}$	$5.09\times 10^{2}$	$5.43\times 10^{2}$	$5.52\times 10^{2}$	$5.22\times 10^{2}$	$5.69\times 10^{2}$	$5.20\times 10^{2}$
	Std	$5.19\times 10^{0}$	$1.37\times 10^{1}$	$4.43\times 10^{0}$	$3.13\times 10^{0}$	$7.67\times 10^{0}$	$9.04\times 10^{0}$	$7.60\times 10^{0}$	$1.17\times 10^{1}$	$9.80\times 10^{0}$
	*p*-value	0.0627	<$10^{-11}$	<$10^{-11}$	0.0005	<$10^{-11}$	<$10^{-11}$	0.0002	<$10^{-11}$	0.0050
	Rank	5	13	11	1	14	16	9	17	7
F6	Mean	$6.14\times 10^{2}$	$6.08\times 10^{2}$	$6.08\times 10^{2}$	$6.03\times 10^{2}$	$6.10\times 10^{2}$	$6.39\times 10^{2}$	$6.14\times 10^{2}$	$6.30\times 10^{2}$	$6.11\times 10^{2}$
	Std	$6.46\times 10^{0}$	$1.02\times 10^{1}$	$9.78\times 10^{-1}$	$1.71\times 10^{0}$	$4.37\times 10^{0}$	$1.45\times 10^{1}$	$7.48\times 10^{0}$	$8.81\times 10^{0}$	$5.34\times 10^{0}$
	*p*-value	<$10^{-11}$	<$10^{-11}$	<$10^{-11}$	<$10^{-11}$	<$10^{-11}$	<$10^{-11}$	<$10^{-11}$	<$10^{-11}$	<$10^{-11}$
	Rank	11	7	7	5	9	17	11	15	10
F7	Mean	$7.59\times 10^{2}$	$7.50\times 10^{2}$	$7.58\times 10^{2}$	$7.26\times 10^{2}$	$7.77\times 10^{2}$	$8.74\times 10^{2}$	$7.52\times 10^{2}$	$8.11\times 10^{2}$	$7.37\times 10^{2}$
	Std	$2.07\times 10^{1}$	$2.10\times 10^{1}$	$5.86\times 10^{0}$	$4.99\times 10^{0}$	$1.42\times 10^{1}$	$4.73\times 10^{1}$	$1.33\times 10^{1}$	$2.62\times 10^{1}$	$9.71\times 10^{0}$
	*p*-value	<$10^{-11}$	<$10^{-11}$	<$10^{-11}$	0.0004	<$10^{-11}$	<$10^{-11}$	<$10^{-11}$	<$10^{-11}$	<$10^{-11}$
	Rank	11	8	10	4	13	17	9	15	7
F8	Mean	$8.35\times 10^{2}$	$8.32\times 10^{2}$	$8.39\times 10^{2}$	$8.19\times 10^{2}$	$8.43\times 10^{2}$	$8.72\times 10^{2}$	$8.38\times 10^{2}$	$8.72\times 10^{2}$	$8.21\times 10^{2}$
	Std	$8.39\times 10^{0}$	$1.17\times 10^{1}$	$4.79\times 10^{0}$	$5.15\times 10^{0}$	$1.11\times 10^{1}$	$1.53\times 10^{1}$	$1.19\times 10^{1}$	$9.57\times 10^{0}$	$9.73\times 10^{0}$
	*p*-value	<$10^{-11}$	<$10^{-11}$	<$10^{-11}$	0.0117	<$10^{-11}$	<$10^{-11}$	<$10^{-11}$	<$10^{-11}$	0.0196
	Rank	9	8	12	5	13	16	11	16	7
F9	Mean	$1.17\times 10^{3}$	$1.04\times 10^{3}$	$9.28\times 10^{2}$	$9.17\times 10^{2}$	$9.87\times 10^{2}$	$3.12\times 10^{3}$	$9.67\times 10^{2}$	$1.42\times 10^{3}$	$9.97\times 10^{2}$
	Std	$1.55\times 10^{2}$	$1.88\times 10^{2}$	$8.86\times 10^{0}$	$1.14\times 10^{1}$	$4.13\times 10^{1}$	$1.20\times 10^{3}$	$6.97\times 10^{1}$	$2.57\times 10^{2}$	$1.00\times 10^{2}$
	*p*-value	<$10^{-11}$	0.0016	0.0003	0.0316	<$10^{-11}$	<$10^{-11}$	<$10^{-11}$	<$10^{-11}$	<$10^{-11}$
	Rank	12	11	7	5	9	18	8	14	10
F10	Mean	$1.93\times 10^{3}$	$2.00\times 10^{3}$	$2.37\times 10^{3}$	$1.64\times 10^{3}$	$2.41\times 10^{3}$	$3.14\times 10^{3}$	$1.73\times 10^{3}$	$2.46\times 10^{3}$	$2.13\times 10^{3}$
	Std	$2.87\times 10^{2}$	$3.27\times 10^{2}$	$1.59\times 10^{2}$	$2.91\times 10^{2}$	$3.13\times 10^{2}$	$3.29\times 10^{2}$	$2.35\times 10^{2}$	$2.62\times 10^{2}$	$3.10\times 10^{2}$
	*p*-value	0.0036	0.0018	<$10^{-11}$	0.3820	<$10^{-11}$	<$10^{-11}$	0.6583	<$10^{-11}$	<$10^{-11}$
	Rank	8	9	14	1	15	18	6	16	12
F11	Mean	$1.16\times 10^{3}$	$1.17\times 10^{3}$	$1.15\times 10^{3}$	$1.13\times 10^{3}$	$1.17\times 10^{3}$	$7.37\times 10^{3}$	$1.16\times 10^{3}$	$1.24\times 10^{3}$	$1.17\times 10^{3}$
	Std	$2.72\times 10^{1}$	$3.93\times 10^{1}$	$8.83\times 10^{0}$	$1.45\times 10^{1}$	$2.63\times 10^{1}$	$4.38\times 10^{3}$	$3.47\times 10^{1}$	$5.67\times 10^{1}$	$3.62\times 10^{1}$
	*p*-value	0.0001	<$10^{-11}$	<$10^{-11}$	0.0243	<$10^{-11}$	<$10^{-11}$	<$10^{-11}$	<$10^{-11}$	<$10^{-11}$
	Rank	8	10	7	6	10	18	8	16	10
F12	Mean	$2.47\times 10^{6}$	$2.25\times 10^{6}$	$4.97\times 10^{6}$	$2.97\times 10^{6}$	$1.52\times 10^{7}$	$2.64\times 10^{9}$	$6.16\times 10^{6}$	$2.51\times 10^{7}$	$1.88\times 10^{6}$
	Std	$2.00\times 10^{6}$	$3.97\times 10^{6}$	$2.33\times 10^{6}$	$2.07\times 10^{6}$	$1.49\times 10^{7}$	$1.80\times 10^{9}$	$7.54\times 10^{6}$	$1.52\times 10^{7}$	$3.03\times 10^{6}$
	*p*-value	<$10^{-11}$	0.0002	<$10^{-11}$	<$10^{-11}$	<$10^{-11}$	<$10^{-11}$	<$10^{-11}$	<$10^{-11}$	0.0016
	Rank	8	7	11	9	15	18	13	16	6
F13	Mean	$1.30\times 10^{4}$	$1.17\times 10^{4}$	$3.17\times 10^{4}$	$1.13\times 10^{4}$	$3.55\times 10^{4}$	$2.74\times 10^{8}$	$9.18\times 10^{3}$	$4.52\times 10^{5}$	$9.54\times 10^{3}$
	Std	$8.60\times 10^{3}$	$1.09\times 10^{4}$	$2.62\times 10^{4}$	$4.40\times 10^{3}$	$3.53\times 10^{4}$	$2.82\times 10^{8}$	$6.27\times 10^{3}$	$4.68\times 10^{5}$	$9.96\times 10^{3}$
	*p*-value	<$10^{-11}$	<$10^{-11}$	<$10^{-11}$	<$10^{-11}$	<$10^{-11}$	<$10^{-11}$	<$10^{-11}$	<$10^{-11}$	<$10^{-11}$
	Rank	11	10	14	9	15	18	6	16	8
F14	Mean	$1.65\times 10^{3}$	$2.55\times 10^{3}$	$1.80\times 10^{3}$	$1.88\times 10^{3}$	$1.75\times 10^{3}$	$2.71\times 10^{6}$	$2.15\times 10^{3}$	$4.94\times 10^{3}$	$1.64\times 10^{3}$
	Std	$2.61\times 10^{2}$	$4.98\times 10^{3}$	$3.56\times 10^{2}$	$6.76\times 10^{2}$	$3.29\times 10^{2}$	$7.17\times 10^{6}$	$1.27\times 10^{3}$	$6.23\times 10^{3}$	$3.76\times 10^{2}$
	*p*-value	<$10^{-11}$	<$10^{-11}$	<$10^{-11}$	<$10^{-11}$	<$10^{-11}$	<$10^{-11}$	<$10^{-11}$	<$10^{-11}$	<$10^{-11}$
	Rank	8	15	11	12	10	17	13	16	7
F15	Mean	$5.00\times 10^{3}$	$7.66\times 10^{3}$	$6.45\times 10^{3}$	$2.99\times 10^{3}$	$5.19\times 10^{3}$	$2.55\times 10^{7}$	$5.37\times 10^{3}$	$2.16\times 10^{4}$	$2.76\times 10^{3}$
	Std	$1.69\times 10^{3}$	$1.36\times 10^{4}$	$2.48\times 10^{3}$	$1.13\times 10^{3}$	$4.06\times 10^{3}$	$1.13\times 10^{8}$	$3.27\times 10^{3}$	$1.88\times 10^{4}$	$1.58\times 10^{3}$
	*p*-value	<$10^{-11}$	<$10^{-11}$	<$10^{-11}$	<$10^{-11}$	<$10^{-11}$	<$10^{-11}$	<$10^{-11}$	<$10^{-11}$	<$10^{-11}$
	Rank	9	15	13	7	10	18	11	16	6
F16	Mean	$1.93\times 10^{3}$	$1.84\times 10^{3}$	$1.73\times 10^{3}$	$1.68\times 10^{3}$	$1.77\times 10^{3}$	$2.20\times 10^{3}$	$1.79\times 10^{3}$	$1.86\times 10^{3}$	$1.77\times 10^{3}$
	Std	$1.99\times 10^{2}$	$1.72\times 10^{2}$	$7.47\times 10^{1}$	$6.32\times 10^{1}$	$1.22\times 10^{2}$	$1.86\times 10^{2}$	$1.17\times 10^{2}$	$1.13\times 10^{2}$	$1.15\times 10^{2}$
	*p*-value	<$10^{-11}$	<$10^{-11}$	<$10^{-11}$	0.0077	<$10^{-11}$	<$10^{-11}$	<$10^{-11}$	<$10^{-11}$	0.0001
	Rank	15	13	7	6	8	17	10	14	8
F17	Mean	$1.81\times 10^{3}$	$1.80\times 10^{3}$	$1.79\times 10^{3}$	$1.75\times 10^{3}$	$1.81\times 10^{3}$	$1.87\times 10^{3}$	$1.78\times 10^{3}$	$1.83\times 10^{3}$	$1.77\times 10^{3}$
	Std	$7.76\times 10^{1}$	$5.68\times 10^{1}$	$1.69\times 10^{1}$	$1.76\times 10^{1}$	$4.75\times 10^{1}$	$8.61\times 10^{1}$	$3.71\times 10^{1}$	$4.31\times 10^{1}$	$3.79\times 10^{1}$
	*p*-value	<$10^{-11}$	<$10^{-11}$	<$10^{-11}$	<$10^{-11}$	<$10^{-11}$	<$10^{-11}$	<$10^{-11}$	<$10^{-11}$	<$10^{-11}$
	Rank	12	10	9	5	12	16	8	15	7
F18	Mean	$5.40\times 10^{3}$	$1.95\times 10^{4}$	$6.07\times 10^{4}$	$5.83\times 10^{3}$	$6.56\times 10^{4}$	$1.07\times 10^{9}$	$1.59\times 10^{4}$	$6.11\times 10^{5}$	$1.11\times 10^{4}$
	Std	$3.14\times 10^{3}$	$1.63\times 10^{4}$	$8.06\times 10^{4}$	$3.68\times 10^{3}$	$7.86\times 10^{4}$	$1.01\times 10^{9}$	$1.21\times 10^{4}$	$1.39\times 10^{6}$	$9.29\times 10^{3}$
	*p*-value	<$10^{-11}$	<$10^{-11}$	<$10^{-11}$	<$10^{-11}$	<$10^{-11}$	<$10^{-11}$	<$10^{-11}$	<$10^{-11}$	<$10^{-11}$
	Rank	6	12	14	7	15	18	10	16	8
F19	Mean	$4.96\times 10^{3}$	$6.72\times 10^{3}$	$4.10\times 10^{3}$	$2.81\times 10^{3}$	$7.32\times 10^{3}$	$1.59\times 10^{8}$	$6.10\times 10^{3}$	$2.99\times 10^{4}$	$3.05\times 10^{3}$
	Std	$4.23\times 10^{3}$	$8.33\times 10^{3}$	$1.51\times 10^{3}$	$1.25\times 10^{3}$	$7.07\times 10^{3}$	$2.72\times 10^{8}$	$6.51\times 10^{3}$	$1.97\times 10^{4}$	$5.65\times 10^{3}$
	*p*-value	<$10^{-11}$	<$10^{-11}$	<$10^{-11}$	<$10^{-11}$	<$10^{-11}$	<$10^{-11}$	<$10^{-11}$	<$10^{-11}$	<$10^{-11}$
	Rank	10	13	8	6	14	18	11	16	7
F20	Mean	$2.16\times 10^{3}$	$2.10\times 10^{3}$	$2.09\times 10^{3}$	$2.04\times 10^{3}$	$2.11\times 10^{3}$	$2.28\times 10^{3}$	$2.09\times 10^{3}$	$2.21\times 10^{3}$	$2.15\times 10^{3}$
	Std	$7.14\times 10^{1}$	$6.45\times 10^{1}$	$1.60\times 10^{1}$	$1.35\times 10^{1}$	$4.53\times 10^{1}$	$1.18\times 10^{2}$	$5.33\times 10^{1}$	$7.00\times 10^{1}$	$7.39\times 10^{1}$
	*p*-value	<$10^{-11}$	<$10^{-11}$	<$10^{-11}$	<$10^{-11}$	<$10^{-11}$	<$10^{-11}$	<$10^{-11}$	<$10^{-11}$	<$10^{-11}$
	Rank	14	9	7	4	10	16	7	15	13
F21	Mean	$2.34\times 10^{3}$	$2.33\times 10^{3}$	$2.22\times 10^{3}$	$2.23\times 10^{3}$	$2.24\times 10^{3}$	$2.38\times 10^{3}$	$2.32\times 10^{3}$	$2.31\times 10^{3}$	$2.32\times 10^{3}$
	Std	$1.84\times 10^{1}$	$2.99\times 10^{1}$	$2.94\times 10^{1}$	$4.41\times 10^{1}$	$5.86\times 10^{1}$	$2.93\times 10^{1}$	$3.98\times 10^{1}$	$6.61\times 10^{1}$	$3.24\times 10^{1}$
	*p*-value	<$10^{-11}$	<$10^{-11}$	0.2623	0.1714	0.8774	<$10^{-11}$	0.0001	0.0077	0.0001
	Rank	15	14	2	3	5	16	12	11	12
F22	Mean	$2.31\times 10^{3}$	$2.40\times 10^{3}$	$2.32\times 10^{3}$	$2.30\times 10^{3}$	$2.33\times 10^{3}$	$4.07\times 10^{3}$	$2.34\times 10^{3}$	$2.38\times 10^{3}$	$2.35\times 10^{3}$
	Std	$2.11\times 10^{1}$	$3.46\times 10^{2}$	$8.51\times 10^{0}$	$1.94\times 10^{1}$	$1.68\times 10^{1}$	$4.60\times 10^{2}$	$1.82\times 10^{2}$	$3.42\times 10^{1}$	$2.37\times 10^{2}$
	*p*-value	0.0020	0.0057	<$10^{-11}$	0.0009	<$10^{-11}$	<$10^{-11}$	0.0024	<$10^{-11}$	0.0002
	Rank	7	16	10	2	12	18	13	15	14
F23	Mean	$2.63\times 10^{3}$	$2.64\times 10^{3}$	$2.63\times 10^{3}$	$2.62\times 10^{3}$	$2.64\times 10^{3}$	$2.72\times 10^{3}$	$2.63\times 10^{3}$	$2.65\times 10^{3}$	$2.63\times 10^{3}$
	Std	$1.51\times 10^{1}$	$1.46\times 10^{1}$	$4.88\times 10^{1}$	$6.09\times 10^{0}$	$8.93\times 10^{0}$	$5.05\times 10^{1}$	$1.09\times 10^{1}$	$8.25\times 10^{0}$	$1.31\times 10^{1}$
	*p*-value	0.0001	<$10^{-11}$	<$10^{-11}$	0.3709	<$10^{-11}$	<$10^{-11}$	<$10^{-11}$	<$10^{-11}$	0.0004
	Rank	7	11	7	3	11	17	7	15	7
F24	Mean	$2.75\times 10^{3}$	$2.77\times 10^{3}$	$2.63\times 10^{3}$	$2.57\times 10^{3}$	$2.73\times 10^{3}$	$2.86\times 10^{3}$	$2.73\times 10^{3}$	$2.79\times 10^{3}$	$2.75\times 10^{3}$
	Std	$6.95\times 10^{1}$	$1.60\times 10^{1}$	$8.16\times 10^{1}$	$9.37\times 10^{1}$	$8.44\times 10^{1}$	$5.56\times 10^{1}$	$6.37\times 10^{1}$	$1.07\times 10^{1}$	$4.95\times 10^{1}$
	*p*-value	0.0003	<$10^{-11}$	0.0041	0.0003	0.0270	<$10^{-11}$	0.0002	<$10^{-11}$	0.0001
	Rank	12	14	3	2	7	17	7	15	12
F25	Mean	$2.93\times 10^{3}$	$2.96\times 10^{3}$	$2.94\times 10^{3}$	$2.94\times 10^{3}$	$2.95\times 10^{3}$	$4.54\times 10^{3}$	$2.93\times 10^{3}$	$2.99\times 10^{3}$	$2.94\times 10^{3}$
	Std	$2.56\times 10^{1}$	$3.90\times 10^{1}$	$1.46\times 10^{1}$	$1.36\times 10^{1}$	$1.87\times 10^{1}$	$6.92\times 10^{2}$	$2.19\times 10^{1}$	$2.01\times 10^{1}$	$2.46\times 10^{1}$
	*p*-value	0.1779	0.0003	0.0407	0.0006	0.0005	<$10^{-11}$	0.0407	<$10^{-11}$	0.0148
	Rank	3	15	10	10	13	17	3	16	10
F26	Mean	$3.22\times 10^{3}$	$3.44\times 10^{3}$	$2.93\times 10^{3}$	$2.89\times 10^{3}$	$2.98\times 10^{3}$	$4.62\times 10^{3}$	$3.07\times 10^{3}$	$3.19\times 10^{3}$	$3.23\times 10^{3}$
	Std	$6.02\times 10^{2}$	$5.55\times 10^{2}$	$4.13\times 10^{1}$	$9.34\times 10^{1}$	$3.99\times 10^{1}$	$2.91\times 10^{302}$	$3.43\times 10^{302}$	$3.29\times 10^{302}$	$4.05\times 10^{302}$
	*p*-value	0.3086	<$10^{-11}$	0.9263	0.2210	0.0039	<$10^{-11}$	0.0897	<$10^{-11}$	0.0014
	Rank	13	15	3	2	6	17	9	12	14
F27	Mean	$3.11\times 10^{3}$	$3.12\times 10^{3}$	$3.11\times 10^{3}$	$3.10\times 10^{3}$	$3.12\times 10^{3}$	$3.39\times 10^{3}$	$3.11\times 10^{3}$	$3.13\times 10^{3}$	$3.18\times 10^{3}$
	Std	$1.84\times 10^{1}$	$5.86\times 10^{0}$	$3.97\times 10^{0}$	$3.11\times 10^{0}$	$1.08\times 10^{1}$	$8.74\times 10^{1}$	$4.02\times 10^{0}$	$1.27\times 10^{1}$	$4.29\times 10^{1}$
	*p*-value	0.0082	<$10^{-11}$	0.0044	0.0028	<$10^{-11}$	<$10^{-11}$	0.0068	<$10^{-11}$	<$10^{-11}$
	Rank	9	12	9	3	12	17	9	14	16
F28	Mean	$3.42\times 10^{3}$	$3.41\times 10^{3}$	$3.26\times 10^{3}$	$3.26\times 10^{3}$	$3.27\times 10^{3}$	$4.15\times 10^{3}$	$3.15\times 10^{3}$	$3.33\times 10^{3}$	$3.36\times 10^{3}$
	Std	$1.33\times 10^{1}$	$1.68\times 10^{2}$	$1.19\times 10^{2}$	$1.17\times 10^{2}$	$9.86\times 10^{1}$	$1.79\times 10^{2}$	$8.72\times 10^{1}$	$1.41\times 10^{2}$	$5.89\times 10^{1}$
	*p*-value	0.0015	0.0036	0.4779	0.2369	0.3389	<$10^{-11}$	0.1714	0.0111	0.1709
	Rank	16	15	3	3	5	17	2	12	13
F29	Mean	$3.32\times 10^{3}$	$3.28\times 10^{3}$	$3.24\times 10^{3}$	$3.20\times 10^{3}$	$3.27\times 10^{3}$	$3.70\times 10^{3}$	$3.19\times 10^{3}$	$3.28\times 10^{3}$	$3.28\times 10^{3}$
	Std	$8.42\times 10^{1}$	$4.98\times 10^{1}$	$2.21\times 10^{1}$	$2.51\times 10^{1}$	$6.81\times 10^{1}$	$1.58\times 10^{2}$	$2.45\times 10^{1}$	$6.92\times 10^{1}$	$7.89\times 10^{1}$
	*p*-value	0.0015	0.0020	0.0285	0.0316	<$10^{-11}$	<$10^{-11}$	0.0536	<$10^{-11}$	<$10^{-11}$
	Rank	15	12	8	7	10	17	2	12	12
F30	Mean	$2.46\times 10^{5}$	$7.20\times 10^{5}$	$3.68\times 10^{5}$	$1.69\times 10^{5}$	$6.52\times 10^{5}$	$6.98\times 10^{7}$	$4.76\times 10^{5}$	$1.34\times 10^{6}$	$8.14\times 10^{5}$
	Std	$4.89\times 10^{5}$	$1.02\times 10^{6}$	$2.16\times 10^{5}$	$2.11\times 10^{5}$	$6.43\times 10^{5}$	$8.68\times 10^{7}$	$8.78\times 10^{3}$	$8.00\times 10^{5}$	$9.44\times 10^{5}$
	*p*-value	0.1986	0.0020	0.2134	0.3185	0.0175	<$10^{-11}$	0.0428	<$10^{-11}$	0.0047
	Rank	4	12	7	2	11	17	8	16	14
+/≈/−		28/0/1	27/0/2	29/0/0	27/1/1	29/0/0	29/0/0	28/0/1	29/0/0	27/0/2
Mean rank		9.31	11.55	8.93	5.45	11.14	17.24	8.66	15.00	9.72
Final ranking		9	14	8	6	13	17	7	16	10

**Table A3:** Comparison of HALA and other algorithms on the CEC2017 benchmark suite (Dim = 30) (excluding F2)—Part 1: HALA vs. ALA, LSHADE, LSHADE-SPACMA, AOOA, BAEO, BPBO, CCO, CEO.

Function	Metric	HALA	ALA	LSHADE	LSHADE- SPACMA	AOOA	BAEO	BPBO	CCO	CEO
F1	Mean	$5.50\times 10^{6}$	$2.67\times 10^{6}$	$3.68\times 10^{9}$	$4.48\times 10^{9}$	$1.73\times 10^{9}$	$1.28\times 10^{11}$	$3.50\times 10^{8}$	$2.94\times 10^{7}$	$3.49\times 10^{7}$
	Std	$4.78\times 10^{6}$	$2.20\times 10^{6}$	$1.85\times 10^{9}$	$2.46\times 10^{9}$	$5.68\times 10^{8}$	$1.84\times 10^{10}$	$1.46\times 10^{8}$	$9.10\times 10^{7}$	$2.46\times 10^{7}$
	*p*-value	–	0.0157	<$10^{-11}$	<$10^{-11}$	<$10^{-11}$	<$10^{-11}$	<$10^{-11}$	0.0125	<$10^{-11}$
	Rank	4	3	10	11	9	18	8	6	7
F3	Mean	$5.12\times 10^{4}$	$3.98\times 10^{4}$	$1.09\times 10^{5}$	$1.12\times 10^{5}$	$8.96\times 10^{4}$	$1.27\times 10^{8}$	$4.22\times 10^{4}$	$8.70\times 10^{4}$	$4.12\times 10^{4}$
	Std	$1.65\times 10^{4}$	$1.02\times 10^{4}$	$3.73\times 10^{4}$	$2.55\times 10^{4}$	$2.39\times 10^{4}$	$6.44\times 10^{8}$	$1.26\times 10^{4}$	$2.83\times 10^{4}$	$1.30\times 10^{4}$
	*p*-value	–	0.0041	<$10^{-11}$	<$10^{-11}$	<$10^{-11}$	<$10^{-11}$	0.0545	<$10^{-11}$	0.0285
	Rank	5	2	11	12	9	18	4	8	3
F4	Mean	$5.34\times 10^{2}$	$5.21\times 10^{2}$	$8.08\times 10^{2}$	$8.97\times 10^{2}$	$7.41\times 10^{2}$	$3.82\times 10^{4}$	$5.62\times 10^{2}$	$6.10\times 10^{2}$	$5.30\times 10^{2}$
	Std	$3.83\times 10^{1}$	$4.45\times 10^{1}$	$1.79\times 10^{2}$	$1.95\times 10^{2}$	$1.12\times 10^{2}$	$9.34\times 10^{3}$	$3.77\times 10^{1}$	$1.21\times 10^{2}$	$4.94\times 10^{1}$
	*p*-value	–	0.544	<$10^{-11}$	<$10^{-11}$	<$10^{-11}$	<$10^{-11}$	0.0028	0.002	0.0387
	Rank	4	2	13	15	11	18	6	9	3
F5	Mean	$5.99\times 10^{2}$	$6.13\times 10^{2}$	$6.24\times 10^{2}$	$6.29\times 10^{2}$	$7.69\times 10^{2}$	$1.11\times 10^{3}$	$6.22\times 10^{2}$	$7.95\times 10^{2}$	$7.17\times 10^{2}$
	Std	$2.51\times 10^{1}$	$3.43\times 10^{1}$	$1.89\times 10^{1}$	$2.12\times 10^{1}$	$5.26\times 10^{1}$	$3.54\times 10^{1}$	$3.08\times 10^{1}$	$6.18\times 10^{1}$	$2.74\times 10^{1}$
	*p*-value	–	<$10^{-11}$	<$10^{-11}$	<$10^{-11}$	<$10^{-11}$	<$10^{-11}$	0.0018	<$10^{-11}$	0.2536
	Rank	1	3	6	7	13	18	5	15	11
F6	Mean	$6.16\times 10^{2}$	$6.14\times 10^{2}$	$6.11\times 10^{2}$	$6.12\times 10^{2}$	$6.60\times 10^{2}$	$7.19\times 10^{2}$	$6.32\times 10^{2}$	$6.63\times 10^{2}$	$6.69\times 10^{2}$
	Std	$8.77\times 10^{0}$	$5.73\times 10^{0}$	$7.10\times 10^{0}$	$5.20\times 10^{0}$	$1.18\times 10^{1}$	$9.65\times 10^{0}$	$9.75\times 10^{0}$	$1.16\times 10^{1}$	$5.69\times 10^{0}$
	*p*-value	–	<$10^{-11}$	<$10^{-11}$	<$10^{-11}$	<$10^{-11}$	<$10^{-11}$	<$10^{-11}$	<$10^{-11}$	0.3493
	Rank	5	4	1	2	12	18	9	13	15
F7	Mean	$9.51\times 10^{2}$	$8.96\times 10^{2}$	$9.05\times 10^{2}$	$9.09\times 10^{2}$	$1.33\times 10^{3}$	$3.48\times 10^{3}$	$9.16\times 10^{2}$	$1.65\times 10^{3}$	$1.40\times 10^{3}$
	Std	$5.35\times 10^{1}$	$4.70\times 10^{1}$	$3.51\times 10^{1}$	$2.93\times 10^{1}$	$2.08\times 10^{2}$	$2.82\times 10^{2}$	$4.08\times 10^{1}$	$2.37\times 10^{2}$	$1.29\times 10^{2}$
	*p*-value	–	<$10^{-11}$	<$10^{-11}$	<$10^{-11}$	<$10^{-11}$	<$10^{-11}$	0.0125	<$10^{-11}$	0.0005
	Rank	5	2	3	4	10	18	6	16	12
F8	Mean	$9.02\times 10^{2}$	$9.08\times 10^{2}$	$9.26\times 10^{2}$	$9.30\times 10^{2}$	$1.07\times 10^{3}$	$1.44\times 10^{3}$	$9.31\times 10^{2}$	$1.10\times 10^{3}$	$1.10\times 10^{3}$
	Std	$2.90\times 10^{1}$	$3.80\times 10^{1}$	$2.49\times 10^{1}$	$2.18\times 10^{1}$	$4.37\times 10^{1}$	$4.84\times 10^{1}$	$2.81\times 10^{1}$	$6.03\times 10^{1}$	$4.28\times 10^{1}$
	*p*-value	–	<$10^{-11}$	<$10^{-11}$	<$10^{-11}$	<$10^{-11}$	<$10^{-11}$	0.0008	<$10^{-11}$	0.517
	Rank	2	3	5	6	10	18	7	12	12
F9	Mean	$3.08\times 10^{3}$	$2.26\times 10^{3}$	$3.06\times 10^{3}$	$3.28\times 10^{3}$	$1.13\times 10^{4}$	$1.44\times 10^{4}$	$3.46\times 10^{3}$	$1.04\times 10^{4}$	$9.05\times 10^{3}$
	Std	$9.14\times 10^{2}$	$8.26\times 10^{2}$	$1.54\times 10^{3}$	$1.33\times 10^{3}$	$3.54\times 10^{3}$	$1.23\times 10^{3}$	$1.12\times 10^{3}$	$2.49\times 10^{3}$	$1.24\times 10^{3}$
	*p*-value	–	<$10^{-11}$	<$10^{-11}$	<$10^{-11}$	<$10^{-11}$	<$10^{-11}$	0.1156	<$10^{-11}$	0.0012
	Rank	4	2	3	5	14	16	6	13	11
F10	Mean	$4.89\times 10^{3}$	$6.38\times 10^{3}$	$5.82\times 10^{3}$	$6.08\times 10^{3}$	$6.69\times 10^{3}$	$8.37\times 10^{3}$	$6.28\times 10^{3}$	$5.27\times 10^{3}$	$6.58\times 10^{3}$
	Std	$6.40\times 10^{2}$	$8.44\times 10^{2}$	$7.27\times 10^{2}$	$7.58\times 10^{2}$	$9.71\times 10^{2}$	$2.22\times 10^{2}$	$8.00\times 10^{2}$	$8.55\times 10^{2}$	$9.31\times 10^{2}$
	*p*-value	–	<$10^{-11}$	<$10^{-11}$	<$10^{-11}$	<$10^{-11}$	<$10^{-11}$	<$10^{-11}$	0.1156	<$10^{-11}$
	Rank	1	9	4	6	11	17	8	3	10
F11	Mean	$1.32\times 10^{3}$	$1.27\times 10^{3}$	$2.48\times 10^{3}$	$3.01\times 10^{3}$	$1.99\times 10^{3}$	$1.03\times 10^{5}$	$1.46\times 10^{3}$	$1.49\times 10^{3}$	$1.37\times 10^{3}$
	Std	$7.89\times 10^{1}$	$5.73\times 10^{1}$	$7.44\times 10^{2}$	$1.23\times 10^{3}$	$4.16\times 10^{2}$	$1.36\times 10^{5}$	$1.08\times 10^{2}$	$1.65\times 10^{2}$	$1.12\times 10^{2}$
	*p*-value	–	0.1359	<$10^{-11}$	<$10^{-11}$	<$10^{-11}$	<$10^{-11}$	0.0001	<$10^{-11}$	0.006
	Rank	3	2	10	13	9	18	5	6	4
F12	Mean	$3.29\times 10^{6}$	$6.62\times 10^{6}$	$1.34\times 10^{8}$	$1.88\times 10^{8}$	$3.18\times 10^{8}$	$3.09\times 10^{10}$	$7.26\times 10^{6}$	$1.98\times 10^{7}$	$1.35\times 10^{7}$
	Std	$3.24\times 10^{6}$	$5.89\times 10^{6}$	$1.14\times 10^{8}$	$1.83\times 10^{8}$	$2.58\times 10^{8}$	$5.96\times 10^{9}$	$6.85\times 10^{6}$	$2.24\times 10^{7}$	$9.26\times 10^{6}$
	*p*-value	–	<$10^{-11}$	<$10^{-11}$	<$10^{-11}$	<$10^{-11}$	<$10^{-11}$	0.0117	<$10^{-11}$	0.0039
	Rank	1	2	10	12	14	18	3	7	6
F13	Mean	$3.10\times 10^{4}$	$3.97\times 10^{4}$	$8.33\times 10^{4}$	$6.04\times 10^{6}$	$1.06\times 10^{7}$	$3.20\times 10^{10}$	$5.98\times 10^{4}$	$1.13\times 10^{5}$	$1.15\times 10^{6}$
	Std	$3.91\times 10^{4}$	$3.04\times 10^{4}$	$6.25\times 10^{4}$	$2.80\times 10^{7}$	$9.01\times 10^{6}$	$1.09\times 10^{10}$	$2.94\times 10^{4}$	$1.03\times 10^{5}$	$1.39\times 10^{6}$
	*p*-value	–	<$10^{-11}$	<$10^{-11}$	<$10^{-11}$	<$10^{-11}$	<$10^{-11}$	0.0016	0.0002	0.0859
	Rank	1	2	3	9	11	18	4	5	8
F14	Mean	$2.36\times 10^{3}$	$1.85\times 10^{3}$	$8.67\times 10^{4}$	$1.61\times 10^{5}$	$5.49\times 10^{5}$	$7.78\times 10^{7}$	$6.80\times 10^{3}$	$1.77\times 10^{5}$	$6.54\times 10^{4}$
	Std	$1.80\times 10^{3}$	$3.19\times 10^{2}$	$1.05\times 10^{5}$	$2.29\times 10^{5}$	$5.61\times 10^{5}$	$5.90\times 10^{7}$	$7.96\times 10^{3}$	$2.43\times 10^{5}$	$5.07\times 10^{4}$
	*p*-value	–	<$10^{-11}$	<$10^{-11}$	<$10^{-11}$	<$10^{-11}$	<$10^{-11}$	0.0004	<$10^{-11}$	0.829
	Rank	3	2	8	10	13	18	4	11	7
F15	Mean	$1.38\times 10^{4}$	$2.25\times 10^{4}$	$2.36\times 10^{4}$	$2.13\times 10^{4}$	$8.99\times 10^{4}$	$9.04\times 10^{9}$	$3.85\times 10^{4}$	$7.47\times 10^{4}$	$5.22\times 10^{4}$
	Std	$1.07\times 10^{4}$	$1.44\times 10^{4}$	$1.02\times 10^{4}$	$9.63\times 10^{3}$	$7.78\times 10^{4}$	$3.69\times 10^{9}$	$1.92\times 10^{4}$	$6.70\times 10^{4}$	$8.33\times 10^{4}$
	*p*-value	–	0.0017	0.0002	0.0018	<$10^{-11}$	<$10^{-11}$	<$10^{-11}$	<$10^{-11}$	0.0041
	Rank	1	3	4	2	10	18	5	9	7
F16	Mean	$2.52\times 10^{3}$	$2.78\times 10^{3}$	$2.72\times 10^{3}$	$2.58\times 10^{3}$	$3.54\times 10^{3}$	$9.51\times 10^{3}$	$2.85\times 10^{3}$	$3.26\times 10^{3}$	$3.39\times 10^{3}$
	Std	$2.39\times 10^{2}$	$3.05\times 10^{2}$	$2.57\times 10^{2}$	$2.10\times 10^{2}$	$4.10\times 10^{2}$	$3.07\times 10^{3}$	$3.79\times 10^{2}$	$4.35\times 10^{2}$	$4.76\times 10^{2}$
	*p*-value	–	<$10^{-11}$	<$10^{-11}$	0.36	<$10^{-11}$	<$10^{-11}$	0.0064	<$10^{-11}$	0.0007
	Rank	1	5	4	3	12	18	6	9	11
F17	Mean	$2.11\times 10^{3}$	$2.22\times 10^{3}$	$2.00\times 10^{3}$	$2.01\times 10^{3}$	$2.63\times 10^{3}$	$5.22\times 10^{4}$	$2.24\times 10^{3}$	$2.77\times 10^{3}$	$2.70\times 10^{3}$
	Std	$1.47\times 10^{2}$	$2.13\times 10^{2}$	$1.22\times 10^{2}$	$1.45\times 10^{2}$	$3.23\times 10^{2}$	$5.51\times 10^{4}$	$1.61\times 10^{2}$	$3.00\times 10^{2}$	$2.89\times 10^{2}$
	*p*-value	–	<$10^{-11}$	0.0104	0.0472	<$10^{-11}$	<$10^{-11}$	0.0166	<$10^{-11}$	0.0285
	Rank	4	6	1	2	10	18	7	13	12
F18	Mean	$7.75\times 10^{4}$	$9.54\times 10^{4}$	$4.44\times 10^{5}$	$4.04\times 10^{5}$	$4.47\times 10^{6}$	$1.26\times 10^{9}$	$1.21\times 10^{5}$	$1.23\times 10^{6}$	$1.02\times 10^{6}$
	Std	$4.56\times 10^{4}$	$5.85\times 10^{4}$	$6.17\times 10^{5}$	$3.78\times 10^{5}$	$5.54\times 10^{6}$	$7.46\times 10^{8}$	$7.74\times 10^{4}$	$2.17\times 10^{6}$	$1.65\times 10^{6}$
	*p*-value	–	<$10^{-11}$	<$10^{-11}$	<$10^{-11}$	<$10^{-11}$	<$10^{-11}$	0.0166	<$10^{-11}$	0.3389
	Rank	1	2	5	4	12	18	3	10	9
F19	Mean	$1.14\times 10^{4}$	$2.29\times 10^{4}$	$2.06\times 10^{5}$	$1.58\times 10^{5}$	$6.05\times 10^{6}$	$8.79\times 10^{9}$	$2.13\times 10^{4}$	$1.70\times 10^{5}$	$2.78\times 10^{5}$
	Std	$7.53\times 10^{3}$	$1.77\times 10^{4}$	$4.67\times 10^{5}$	$2.97\times 10^{5}$	$1.18\times 10^{7}$	$3.95\times 10^{9}$	$2.25\times 10^{4}$	$3.05\times 10^{5}$	$4.87\times 10^{5}$
	*p*-value	–	<$10^{-11}$	<$10^{-11}$	<$10^{-11}$	<$10^{-11}$	<$10^{-11}$	0.0786	<$10^{-11}$	0.0034
	Rank	1	3	8	7	12	18	2	6	9
F20	Mean	$2.42\times 10^{3}$	$2.56\times 10^{3}$	$2.44\times 10^{3}$	$2.40\times 10^{3}$	$2.67\times 10^{3}$	$3.24\times 10^{3}$	$2.47\times 10^{3}$	$2.84\times 10^{3}$	$3.07\times 10^{3}$
	Std	$1.56\times 10^{2}$	$1.72\times 10^{2}$	$1.23\times 10^{2}$	$1.35\times 10^{2}$	$1.74\times 10^{2}$	$1.21\times 10^{2}$	$1.53\times 10^{2}$	$2.05\times 10^{2}$	$2.40\times 10^{2}$
	*p*-value	–	<$10^{-11}$	<$10^{-11}$	0.9099	<$10^{-11}$	<$10^{-11}$	0.1589	<$10^{-11}$	0.0098
	Rank	3	7	4	2	9	18	5	12	16
F21	Mean	$2.40\times 10^{3}$	$2.41\times 10^{3}$	$2.42\times 10^{3}$	$2.43\times 10^{3}$	$2.57\times 10^{3}$	$2.93\times 10^{3}$	$2.42\times 10^{3}$	$2.59\times 10^{3}$	$2.59\times 10^{3}$
	Std	$2.89\times 10^{1}$	$3.18\times 10^{1}$	$2.07\times 10^{1}$	$2.37\times 10^{1}$	$5.54\times 10^{1}$	$5.14\times 10^{1}$	$2.87\times 10^{1}$	$5.99\times 10^{1}$	$5.23\times 10^{1}$
	*p*-value	–	<$10^{-11}$	<$10^{-11}$	<$10^{-11}$	<$10^{-11}$	<$10^{-11}$	0.0243	<$10^{-11}$	0.829
	Rank	1	3	4	5	11	18	4	13	13
F22	Mean	$3.62\times 10^{3}$	$6.83\times 10^{3}$	$3.41\times 10^{3}$	$2.87\times 10^{3}$	$5.34\times 10^{3}$	$1.02\times 10^{4}$	$2.51\times 10^{3}$	$6.36\times 10^{3}$	$6.42\times 10^{3}$
	Std	$1.92\times 10^{3}$	$1.95\times 10^{3}$	$1.64\times 10^{3}$	$4.47\times 10^{2}$	$2.61\times 10^{3}$	$3.03\times 10^{2}$	$7.32\times 10^{1}$	$2.09\times 10^{3}$	$2.60\times 10^{3}$
	*p*-value	–	0.0003	0.6435	0.6435	0.0044	<$10^{-11}$	0.6435	0.0001	<$10^{-11}$
	Rank	5	11	4	3	8	18	2	9	10
F23	Mean	$2.77\times 10^{3}$	$2.77\times 10^{3}$	$2.81\times 10^{3}$	$2.80\times 10^{3}$	$3.03\times 10^{3}$	$3.97\times 10^{3}$	$2.78\times 10^{3}$	$3.07\times 10^{3}$	$3.33\times 10^{3}$
	Std	$2.97\times 10^{1}$	$2.96\times 10^{1}$	$2.80\times 10^{1}$	$3.22\times 10^{1}$	$1.02\times 10^{2}$	$1.65\times 10^{2}$	$3.12\times 10^{1}$	$1.19\times 10^{2}$	$1.99\times 10^{2}$
	*p*-value	–	<$10^{-11}$	<$10^{-11}$	0.0006	<$10^{-11}$	<$10^{-11}$	0.0822	<$10^{-11}$	0.4779
	Rank	2	2	7	6	11	18	4	12	16
F24	Mean	$2.92\times 10^{3}$	$2.96\times 10^{3}$	$2.97\times 10^{3}$	$2.97\times 10^{3}$	$3.14\times 10^{3}$	$4.45\times 10^{3}$	$2.95\times 10^{3}$	$3.19\times 10^{3}$	$3.49\times 10^{3}$
	Std	$2.81\times 10^{1}$	$3.87\times 10^{1}$	$3.52\times 10^{1}$	$2.48\times 10^{1}$	$7.67\times 10^{1}$	$2.92\times 10^{2}$	$2.57\times 10^{1}$	$1.05\times 10^{2}$	$1.71\times 10^{2}$
	*p*-value	–	<$10^{-11}$	<$10^{-11}$	<$10^{-11}$	<$10^{-11}$	<$10^{-11}$	0.0004	<$10^{-11}$	0.0001
	Rank	1	5	6	6	11	18	4	12	16
F25	Mean	$2.93\times 10^{3}$	$2.91\times 10^{3}$	$3.12\times 10^{3}$	$3.14\times 10^{3}$	$3.16\times 10^{3}$	$1.99\times 10^{4}$	$2.96\times 10^{3}$	$2.97\times 10^{3}$	$2.91\times 10^{3}$
	Std	$2.33\times 10^{1}$	$2.17\times 10^{1}$	$7.48\times 10^{1}$	$6.84\times 10^{1}$	$1.36\times 10^{2}$	$4.73\times 10^{3}$	$2.25\times 10^{1}$	$4.29\times 10^{1}$	$1.75\times 10^{1}$
	*p*-value	–	0.0002	<$10^{-11}$	<$10^{-11}$	<$10^{-11}$	<$10^{-11}$	0.0002	0.006	0.0036
	Rank	4	2	12	13	14	18	7	8	2
F26	Mean	$4.85\times 10^{3}$	$4.91\times 10^{3}$	$5.50\times 10^{3}$	$5.47\times 10^{3}$	$7.21\times 10^{3}$	$1.75\times 10^{4}$	$4.18\times 10^{3}$	$8.05\times 10^{3}$	$8.43\times 10^{3}$
	Std	$1.02\times 10^{3}$	$5.31\times 10^{2}$	$5.75\times 10^{2}$	$6.64\times 10^{2}$	$1.64\times 10^{3}$	$2.05\times 10^{3}$	$5.92\times 10^{2}$	$1.47\times 10^{3}$	$1.01\times 10^{3}$
	*p*-value	–	<$10^{-11}$	<$10^{-11}$	0.035	<$10^{-11}$	<$10^{-11}$	0.0064	<$10^{-11}$	0.6143
	Rank	4	5	9	8	12	18	2	14	15
F27	Mean	$3.25\times 10^{3}$	$3.23\times 10^{3}$	$3.29\times 10^{3}$	$3.30\times 10^{3}$	$3.39\times 10^{3}$	$5.84\times 10^{3}$	$3.25\times 10^{3}$	$3.38\times 10^{3}$	$3.44\times 10^{3}$
	Std	$2.25\times 10^{1}$	$2.08\times 10^{1}$	$3.01\times 10^{1}$	$2.87\times 10^{1}$	$9.67\times 10^{1}$	$5.58\times 10^{2}$	$1.47\times 10^{1}$	$7.82\times 10^{1}$	$1.49\times 10^{2}$
	*p*-value	–	<$10^{-11}$	<$10^{-11}$	<$10^{-11}$	<$10^{-11}$	<$10^{-11}$	0.7971	<$10^{-11}$	0.0256
	Rank	3	2	5	6	10	18	3	9	13
F28	Mean	$3.30\times 10^{3}$	$3.80\times 10^{3}$	$3.70\times 10^{3}$	$3.81\times 10^{3}$	$3.54\times 10^{3}$	$1.48\times 10^{4}$	$3.37\times 10^{3}$	$3.60\times 10^{3}$	$3.28\times 10^{3}$
	Std	$3.17\times 10^{1}$	$1.14\times 10^{3}$	$2.33\times 10^{2}$	$2.79\times 10^{2}$	$1.26\times 10^{2}$	$2.60\times 10^{3}$	$4.21\times 10^{1}$	$5.41\times 10^{2}$	$2.67\times 10^{1}$
	*p*-value	–	0.005	<$10^{-11}$	<$10^{-11}$	<$10^{-11}$	<$10^{-11}$	<$10^{-11}$	<$10^{-11}$	0.0656
	Rank	3	12	10	13	8	18	5	9	2
F29	Mean	$3.95\times 10^{3}$	$4.01\times 10^{3}$	$4.17\times 10^{3}$	$4.18\times 10^{3}$	$4.92\times 10^{3}$	$6.64\times 10^{4}$	$4.27\times 10^{3}$	$4.81\times 10^{3}$	$4.69\times 10^{3}$
	Std	$2.05\times 10^{2}$	$2.26\times 10^{2}$	$2.62\times 10^{2}$	$2.27\times 10^{2}$	$4.14\times 10^{2}$	$1.25\times 10^{5}$	$2.41\times 10^{2}$	$4.85\times 10^{2}$	$3.64\times 10^{2}$
	*p*-value	–	<$10^{-11}$	<$10^{-11}$	<$10^{-11}$	<$10^{-11}$	<$10^{-11}$	0.0001	<$10^{-11}$	0.1846
	Rank	1	2	4	5	12	18	6	11	10
F30	Mean	$5.60\times 10^{4}$	$5.69\times 10^{4}$	$4.24\times 10^{6}$	$3.96\times 10^{6}$	$1.66\times 10^{7}$	$5.55\times 10^{9}$	$5.13\times 10^{5}$	$1.08\times 10^{6}$	$1.40\times 10^{6}$
	Std	$7.62\times 10^{4}$	$5.67\times 10^{4}$	$3.50\times 10^{6}$	$4.07\times 10^{6}$	$2.20\times 10^{7}$	$1.89\times 10^{9}$	$4.23\times 10^{5}$	$1.30\times 10^{6}$	$1.46\times 10^{6}$
	*p*-value	–	<$10^{-11}$	<$10^{-11}$	<$10^{-11}$	<$10^{-11}$	<$10^{-11}$	<$10^{-11}$	<$10^{-11}$	0.7655
	Rank	1	2	9	8	14	18	4	5	7
+/≈/−		–	9/12/8	23/3/3	22/5/2	29/0/0	29/0/0	20/7/2	28/1/0	24/2/3
Mean rank		2.38	3.79	6.10	6.31	11.62	17.83	4.83	9.62	8.93
Final ranking		1	2	6	7	14	18	4	12	10

**Table A4:** Comparison of HALA and other algorithms on the CEC2017 benchmark suite (Dim = 30) (excluding F2)—Part 2: HALA vs. CQALA, DFL, DMOA, DHOA, FGO, KLA, PGA, SO, SOO (continued from Table A3).

Function	Metric	CQALA	DFL	DMOA	DHOA	FGO	KLA	PGA	SO	SOO
F1	Mean	$1.66\times 10^{5}$	$1.22\times 10^{8}$	$2.14\times 10^{9}$	$5.90\times 10^{8}$	$6.40\times 10^{9}$	$1.27\times 10^{11}$	$1.71\times 10^{7}$	$2.41\times 10^{10}$	$1.65\times 10^{9}$
	Std	$3.29\times 10^{5}$	$4.02\times 10^{7}$	$2.58\times 10^{8}$	$3.84\times 10^{8}$	$2.17\times 10^{9}$	$1.78\times 10^{10}$	$3.75\times 10^{6}$	$6.48\times 10^{9}$	$1.19\times 10^{9}$
	*p*-value	<$10^{-11}$	<$10^{-11}$	<$10^{-11}$	<$10^{-11}$	<$10^{-11}$	<$10^{-11}$	<$10^{-11}$	<$10^{-11}$	<$10^{-11}$
	Rank	2	5	12	13	14	17	1	15	16
F3	Mean	$7.18\times 10^{4}$	$1.76\times 10^{5}$	$1.12\times 10^{5}$	$7.94\times 10^{4}$	$1.04\times 10^{5}$	$2.38\times 10^{10}$	$1.10\times 10^{5}$	$2.86\times 10^{5}$	$2.09\times 10^{5}$
	Std	$1.48\times 10^{4}$	$4.97\times 10^{4}$	$1.94\times 10^{4}$	$1.33\times 10^{4}$	$2.14\times 10^{4}$	$5.57\times 10^{10}$	$2.79\times 10^{4}$	$5.42\times 10^{4}$	$6.99\times 10^{4}$
	*p*-value	<$10^{-11}$	<$10^{-11}$	<$10^{-11}$	<$10^{-11}$	<$10^{-11}$	<$10^{-11}$	<$10^{-11}$	<$10^{-11}$	<$10^{-11}$
	Rank	7	14	13	10	12	17	6	15	16
F4	Mean	$5.46\times 10^{2}$	$5.81\times 10^{2}$	$6.34\times 10^{2}$	$6.10\times 10^{2}$	$9.39\times 10^{2}$	$3.44\times 10^{4}$	$5.62\times 10^{2}$	$3.11\times 10^{3}$	$6.63\times 10^{2}$
	Std	$4.73\times 10^{1}$	$6.43\times 10^{1}$	$3.75\times 10^{1}$	$2.89\times 10^{1}$	$1.93\times 10^{2}$	$9.19\times 10^{3}$	$4.06\times 10^{1}$	$9.17\times 10^{2}$	$7.56\times 10^{1}$
	*p*-value	0.3286	0.0008	<$10^{-11}$	<$10^{-11}$	<$10^{-11}$	<$10^{-11}$	0.0098	<$10^{-11}$	<$10^{-11}$
	Rank	1	5	10	8	16	17	7	14	12
F5	Mean	$6.59\times 10^{2}$	$7.39\times 10^{2}$	$7.34\times 10^{2}$	$6.21\times 10^{2}$	$7.97\times 10^{2}$	$8.73\times 10^{2}$	$7.10\times 10^{2}$	$9.15\times 10^{2}$	$6.44\times 10^{2}$
	Std	$2.65\times 10^{1}$	$6.16\times 10^{1}$	$1.10\times 10^{1}$	$2.22\times 10^{1}$	$3.21\times 10^{1}$	$3.31\times 10^{1}$	$4.38\times 10^{1}$	$4.52\times 10^{1}$	$2.78\times 10^{1}$
	*p*-value	<$10^{-11}$	<$10^{-11}$	<$10^{-11}$	0.0028	<$10^{-11}$	<$10^{-11}$	<$10^{-11}$	<$10^{-11}$	<$10^{-11}$
	Rank	10	14	12	4	16	17	9	18	8
F6	Mean	$6.46\times 10^{2}$	$6.53\times 10^{2}$	$6.20\times 10^{2}$	$6.25\times 10^{2}$	$6.45\times 10^{2}$	$6.78\times 10^{2}$	$6.55\times 10^{2}$	$6.80\times 10^{2}$	$6.42\times 10^{2}$
	Std	$6.20\times 10^{0}$	$1.69\times 10^{1}$	$1.85\times 10^{0}$	$6.16\times 10^{0}$	$1.15\times 10^{1}$	$6.86\times 10^{0}$	$1.09\times 10^{1}$	$9.17\times 10^{0}$	$8.69\times 10^{0}$
	*p*-value	<$10^{-11}$	<$10^{-11}$	0.035	0.0002	<$10^{-11}$	<$10^{-11}$	<$10^{-11}$	<$10^{-11}$	<$10^{-11}$
	Rank	11	14	6	7	10	17	15	16	3
F7	Mean	$1.17\times 10^{3}$	$1.24\times 10^{3}$	$1.03\times 10^{3}$	$1.01\times 10^{3}$	$1.17\times 10^{3}$	$2.14\times 10^{3}$	$1.14\times 10^{3}$	$1.84\times 10^{3}$	$1.10\times 10^{3}$
	Std	$8.99\times 10^{1}$	$1.29\times 10^{2}$	$1.18\times 10^{1}$	$4.44\times 10^{1}$	$8.54\times 10^{1}$	$4.52\times 10^{2}$	$9.38\times 10^{1}$	$1.74\times 10^{2}$	$6.58\times 10^{1}$
	*p*-value	<$10^{-11}$	<$10^{-11}$	<$10^{-11}$	0.0006	<$10^{-11}$	<$10^{-11}$	<$10^{-11}$	<$10^{-11}$	<$10^{-11}$
	Rank	11	13	7	4	11	17	9	15	8
F8	Mean	$1.00\times 10^{3}$	$1.03\times 10^{3}$	$1.03\times 10^{3}$	$9.39\times 10^{2}$	$1.09\times 10^{3}$	$1.22\times 10^{3}$	$9.98\times 10^{2}$	$1.17\times 10^{3}$	$9.49\times 10^{2}$
	Std	$3.77\times 10^{1}$	$5.92\times 10^{1}$	$1.44\times 10^{1}$	$2.43\times 10^{1}$	$3.54\times 10^{1}$	$4.79\times 10^{1}$	$4.58\times 10^{1}$	$3.77\times 10^{1}$	$3.33\times 10^{1}$
	*p*-value	<$10^{-11}$	<$10^{-11}$	<$10^{-11}$	0.0003	<$10^{-11}$	<$10^{-11}$	<$10^{-11}$	<$10^{-11}$	<$10^{-11}$
	Rank	8	10	10	1	14	17	4	15	9
F9	Mean	$5.05\times 10^{3}$	$1.18\times 10^{4}$	$1.75\times 10^{3}$	$2.60\times 10^{3}$	$8.92\times 10^{3}$	$3.52\times 10^{4}$	$9.39\times 10^{3}$	$1.79\times 10^{4}$	$5.38\times 10^{3}$
	Std	$4.95\times 10^{2}$	$4.96\times 10^{3}$	$2.31\times 10^{2}$	$6.84\times 10^{2}$	$4.56\times 10^{3}$	$1.04\times 10^{4}$	$3.10\times 10^{3}$	$4.66\times 10^{3}$	$1.65\times 10^{3}$
	*p*-value	<$10^{-11}$	<$10^{-11}$	0.8612	0.0449	<$10^{-11}$	<$10^{-11}$	<$10^{-11}$	<$10^{-11}$	<$10^{-11}$
	Rank	7	15	1	3	10	18	12	17	8
F10	Mean	$5.01\times 10^{3}$	$7.08\times 10^{3}$	$8.55\times 10^{3}$	$5.59\times 10^{3}$	$8.69\times 10^{3}$	$1.00\times 10^{4}$	$5.51\times 10^{3}$	$8.53\times 10^{3}$	$6.24\times 10^{3}$
	Std	$1.08\times 10^{3}$	$6.61\times 10^{62}$	$2.33\times 10^{2}$	$7.71\times 10^{2}$	$6.92\times 10^{62}$	$4.80\times 10^{2}$	$5.17\times 10^{2}$	$4.62\times 10^{2}$	$5.91\times 10^{62}$
	*p*-value	0.9099	<$10^{-11}$	<$10^{-11}$	0.0024	<$10^{-11}$	<$10^{-11}$	0.0024	<$10^{-11}$	<$10^{-11}$
	Rank	2	12	15	5	16	18	4	14	7
F11	Mean	$1.29\times 10^{3}$	$1.53\times 10^{3}$	$2.17\times 10^{3}$	$1.39\times 10^{3}$	$2.26\times 10^{3}$	$2.56\times 10^{5}$	$1.49\times 10^{3}$	$1.29\times 10^{4}$	$3.08\times 10^{3}$
	Std	$6.52\times 10^{1}$	$1.52\times 10^{2}$	$2.22\times 10^{2}$	$6.75\times 10^{1}$	$4.30\times 10^{2}$	$7.11\times 10^{5}$	$1.11\times 10^{2}$	$4.06\times 10^{3}$	$2.00\times 10^{3}$
	*p*-value	0.1204	<$10^{-11}$	<$10^{-11}$	0.0016	<$10^{-11}$	<$10^{-11}$	<$10^{-11}$	<$10^{-11}$	<$10^{-11}$
	Rank	1	7	11	5	10	17	6	16	15
F12	Mean	$4.86\times 10^{6}$	$1.44\times 10^{8}$	$3.46\times 10^{8}$	$2.05\times 10^{8}$	$7.02\times 10^{8}$	$3.18\times 10^{10}$	$1.10\times 10^{8}$	$1.73\times 10^{9}$	$2.48\times 10^{7}$
	Std	$5.84\times 10^{6}$	$1.30\times 10^{8}$	$7.23\times 10^{7}$	$1.27\times 10^{9}$	$6.12\times 10^{9}$	$4.57\times 10^{10}$	$6.91\times 10^{8}$	$1.77\times 10^{10}$	$4.75\times 10^{9}$
	*p*-value	0.544	<$10^{-11}$	<$10^{-11}$	<$10^{-11}$	<$10^{-11}$	<$10^{-11}$	<$10^{-11}$	<$10^{-11}$	0.0001
	Rank	4	11	15	13	16	17	9	18	5
F13	Mean	$1.93\times 10^{4}$	$9.68\times 10^{5}$	$1.17\times 10^{8}$	$5.05\times 10^{5}$	$2.27\times 10^{8}$	$3.27\times 10^{10}$	$2.82\times 10^{6}$	$1.56\times 10^{10}$	$8.23\times 10^{8}$
	Std	$5.72\times 10^{4}$	$1.93\times 10^{6}$	$1.78\times 10^{8}$	$8.35\times 10^{5}$	$1.46\times 10^{9}$	$1.46\times 10^{10}$	$4.94\times 10^{5}$	$3.67\times 10^{9}$	$6.03\times 10^{8}$
	*p*-value	0.0117	<$10^{-11}$	<$10^{-11}$	<$10^{-11}$	<$10^{-11}$	<$10^{-11}$	<$10^{-11}$	<$10^{-11}$	0.0008
	Rank	2	7	13	6	12	17	10	16	15
F14	Mean	$2.63\times 10^{5}$	$5.14\times 10^{4}$	$3.29\times 10^{5}$	$1.57\times 10^{5}$	$3.37\times 10^{5}$	$7.77\times 10^{7}$	$2.35\times 10^{5}$	$1.38\times 10^{6}$	$5.02\times 10^{5}$
	Std	$1.77\times 10^{5}$	$5.04\times 10^{4}$	$2.03\times 10^{5}$	$1.18\times 10^{5}$	$2.95\times 10^{5}$	$6.27\times 10^{8}$	$2.15\times 10^{5}$	$1.35\times 10^{6}$	$8.40\times 10^{5}$
	*p*-value	<$10^{-11}$	<$10^{-11}$	<$10^{-11}$	<$10^{-11}$	<$10^{-11}$	<$10^{-11}$	<$10^{-11}$	<$10^{-11}$	<$10^{-11}$
	Rank	12	1	14	9	15	17	6	16	13
F15	Mean	$3.25\times 10^{3}$	$8.46\times 10^{4}$	$1.58\times 10^{7}$	$4.31\times 10^{5}$	$2.64\times 10^{7}$	$7.14\times 10^{9}$	$7.51\times 10^{4}$	$1.28\times 10^{8}$	$1.62\times 10^{4}$
	Std	$1.55\times 10^{3}$	$6.03\times 10^{4}$	$6.78\times 10^{6}$	$6.04\times 10^{5}$	$3.17\times 10^{7}$	$1.07\times 10^{10}$	$3.38\times 10^{4}$	$9.09\times 10^{7}$	$1.41\times 10^{4}$
	*p*-value	<$10^{-11}$	<$10^{-11}$	<$10^{-11}$	<$10^{-11}$	<$10^{-11}$	<$10^{-11}$	<$10^{-11}$	<$10^{-11}$	0.7655
	Rank	3	8	14	11	15	17	6	16	4
F16	Mean	$3.06\times 10^{3}$	$3.12\times 10^{3}$	$3.52\times 10^{3}$	$2.68\times 10^{3}$	$3.81\times 10^{3}$	$7.67\times 10^{3}$	$3.16\times 10^{3}$	$4.29\times 10^{3}$	$3.08\times 10^{3}$
	Std	$3.26\times 10^{2}$	$4.20\times 10^{2}$	$2.31\times 10^{2}$	$2.24\times 10^{2}$	$3.27\times 10^{302}$	$2.58\times 10^{3}$	$3.36\times 10^{302}$	$3.12\times 10^{302}$	$4.14\times 10^{302}$
	*p*-value	<$10^{-11}$	<$10^{-11}$	<$10^{-11}$	0.0157	<$10^{-11}$	<$10^{-11}$	<$10^{-11}$	<$10^{-11}$	<$10^{-11}$
	Rank	7	8	13	2	14	17	10	16	15
F17	Mean	$2.50\times 10^{3}$	$2.53\times 10^{3}$	$2.30\times 10^{3}$	$2.02\times 10^{3}$	$2.65\times 10^{3}$	$8.55\times 10^{4}$	$2.38\times 10^{3}$	$3.24\times 10^{3}$	$2.49\times 10^{3}$
	Std	$2.65\times 10^{2}$	$2.84\times 10^{2}$	$1.19\times 10^{2}$	$1.54\times 10^{2}$	$2.52\times 10^{2}$	$1.43\times 10^{5}$	$2.55\times 10^{2}$	$2.53\times 10^{2}$	$3.02\times 10^{2}$
	*p*-value	<$10^{-11}$	<$10^{-11}$	<$10^{-11}$	0.03	<$10^{-11}$	<$10^{-11}$	0.0002	<$10^{-11}$	<$10^{-11}$
	Rank	8	9	5	3	11	17	5	15	7
F18	Mean	$2.87\times 10^{5}$	$1.18\times 10^{6}$	$2.49\times 10^{6}$	$5.79\times 10^{5}$	$5.89\times 10^{6}$	$8.29\times 10^{8}$	$2.93\times 10^{6}$	$1.74\times 10^{7}$	$1.75\times 10^{6}$
	Std	$3.94\times 10^{5}$	$1.22\times 10^{6}$	$1.37\times 10^{6}$	$4.72\times 10^{5}$	$3.99\times 10^{6}$	$5.41\times 10^{8}$	$2.62\times 10^{6}$	$5.39\times 10^{7}$	$2.41\times 10^{6}$
	*p*-value	0.0005	<$10^{-11}$	<$10^{-11}$	<$10^{-11}$	<$10^{-11}$	<$10^{-11}$	<$10^{-11}$	<$10^{-11}$	<$10^{-11}$
	Rank	4	7	11	6	13	17	12	16	8
F19	Mean	$6.17\times 10^{3}$	$2.70\times 10^{5}$	$2.65\times 10^{7}$	$5.89\times 10^{7}$	$4.93\times 10^{7}$	$5.77\times 10^{10}$	$6.19\times 10^{6}$	$4.80\times 10^{9}$	$1.27\times 10^{8}$
	Std	$4.74\times 10^{3}$	$4.46\times 10^{5}$	$9.75\times 10^{7}$	$3.73\times 10^{7}$	$6.63\times 10^{8}$	$1.03\times 10^{10}$	$4.98\times 10^{6}$	$1.71\times 10^{9}$	$1.92\times 10^{8}$
	*p*-value	0.005	<$10^{-11}$	<$10^{-11}$	<$10^{-11}$	<$10^{-11}$	<$10^{-11}$	<$10^{-11}$	<$10^{-11}$	0.5038
	Rank	3	10	13	14	15	17	11	16	12
F20	Mean	$2.62\times 10^{3}$	$2.80\times 10^{3}$	$2.74\times 10^{3}$	$2.43\times 10^{3}$	$2.84\times 10^{3}$	$3.43\times 10^{3}$	$2.55\times 10^{3}$	$3.06\times 10^{3}$	$2.76\times 10^{3}$
	Std	$2.49\times 10^{2}$	$2.42\times 10^{2}$	$1.26\times 10^{2}$	$1.16\times 10^{2}$	$2.03\times 10^{2}$	$2.38\times 10^{2}$	$1.48\times 10^{2}$	$1.76\times 10^{2}$	$2.60\times 10^{2}$
	*p*-value	0.0018	<$10^{-11}$	<$10^{-11}$	0.9099	<$10^{-11}$	<$10^{-11}$	0.0002	<$10^{-11}$	<$10^{-11}$
	Rank	8	11	10	1	13	17	6	15	14
F21	Mean	$2.48\times 10^{3}$	$2.54\times 10^{3}$	$2.52\times 10^{3}$	$2.42\times 10^{3}$	$2.57\times 10^{3}$	$2.76\times 10^{3}$	$2.50\times 10^{3}$	$2.66\times 10^{3}$	$2.45\times 10^{3}$
	Std	$4.25\times 10^{1}$	$4.58\times 10^{1}$	$1.27\times 10^{1}$	$1.97\times 10^{1}$	$3.01\times 10^{1}$	$6.69\times 10^{1}$	$4.61\times 10^{1}$	$3.08\times 10^{1}$	$4.51\times 10^{1}$
	*p*-value	<$10^{-11}$	<$10^{-11}$	<$10^{-11}$	0.03	<$10^{-11}$	<$10^{-11}$	<$10^{-11}$	<$10^{-11}$	0.0004
	Rank	7	10	9	2	12	17	8	15	6
F22	Mean	$3.42\times 10^{3}$	$8.09\times 10^{3}$	$2.64\times 10^{3}$	$2.44\times 10^{3}$	$4.15\times 10^{3}$	$1.14\times 10^{4}$	$6.27\times 10^{3}$	$9.88\times 10^{3}$	$6.18\times 10^{3}$
	Std	$1.96\times 10^{3}$	$1.61\times 10^{3}$	$3.01\times 10^{1}$	$6.12\times 10^{1}$	$2.40\times 10^{3}$	$6.95\times 10^{2}$	$1.93\times 10^{3}$	$9.60\times 10^{2}$	$1.92\times 10^{3}$
	*p*-value	0.7343	<$10^{-11}$	0.6435	0.6435	0.221	<$10^{-11}$	<$10^{-11}$	<$10^{-11}$	<$10^{-11}$
	Rank	6	13	7	1	7	17	12	15	11
F23	Mean	$2.92\times 10^{3}$	$2.96\times 10^{3}$	$2.88\times 10^{3}$	$2.79\times 10^{3}$	$2.94\times 10^{3}$	$3.59\times 10^{3}$	$2.92\times 10^{3}$	$3.03\times 10^{3}$	$2.87\times 10^{3}$
	Std	$9.06\times 10^{1}$	$9.29\times 10^{1}$	$1.62\times 10^{1}$	$2.11\times 10^{1}$	$5.32\times 10^{1}$	$1.88\times 10^{2}$	$6.63\times 10^{1}$	$4.46\times 10^{1}$	$4.85\times 10^{1}$
	*p*-value	<$10^{-11}$	<$10^{-11}$	<$10^{-11}$	0.0024	<$10^{-11}$	<$10^{-11}$	<$10^{-11}$	<$10^{-11}$	<$10^{-11}$
	Rank	9	10	5	3	8	17	9	13	14
F24	Mean	$3.11\times 10^{3}$	$3.09\times 10^{3}$	$3.03\times 10^{3}$	$2.94\times 10^{3}$	$3.08\times 10^{3}$	$3.81\times 10^{3}$	$3.03\times 10^{3}$	$3.14\times 10^{3}$	$3.02\times 10^{3}$
	Std	$6.07\times 10^{1}$	$6.61\times 10^{1}$	$1.39\times 10^{1}$	$2.09\times 10^{1}$	$2.55\times 10^{1}$	$2.51\times 10^{2}$	$6.79\times 10^{1}$	$3.69\times 10^{1}$	$6.49\times 10^{1}$
	*p*-value	<$10^{-11}$	<$10^{-11}$	<$10^{-11}$	0.0003	<$10^{-11}$	<$10^{-11}$	<$10^{-11}$	<$10^{-11}$	<$10^{-11}$
	Rank	13	10	8	3	9	17	8	14	15
F25	Mean	$2.92\times 10^{3}$	$3.01\times 10^{3}$	$3.05\times 10^{3}$	$3.04\times 10^{3}$	$3.28\times 10^{3}$	$1.93\times 10^{4}$	$2.97\times 10^{3}$	$5.93\times 10^{3}$	$3.05\times 10^{3}$
	Std	$2.18\times 10^{1}$	$6.69\times 10^{1}$	$2.24\times 10^{1}$	$3.97\times 10^{1}$	$1.60\times 10^{2}$	$4.58\times 10^{3}$	$4.77\times 10^{1}$	$1.35\times 10^{3}$	$5.33\times 10^{1}$
	*p*-value	0.0117	<$10^{-11}$	<$10^{-11}$	<$10^{-11}$	<$10^{-11}$	<$10^{-11}$	0.0013	<$10^{-11}$	<$10^{-11}$
	Rank	3	9	11	10	15	17	6	16	11
F26	Mean	$7.03\times 10^{3}$	$7.17\times 10^{3}$	$4.74\times 10^{3}$	$5.46\times 10^{3}$	$6.49\times 10^{3}$	$1.53\times 10^{4}$	$6.76\times 10^{3}$	$7.52\times 10^{3}$	$5.88\times 10^{3}$
	Std	$1.06\times 10^{3}$	$1.25\times 10^{3}$	$1.21\times 10^{3}$	$1.48\times 10^{3}$	$5.10\times 10^{2}$	$2.01\times 10^{3}$	$9.43\times 10^{2}$	$4.77\times 10^{2}$	$7.34\times 10^{2}$
	*p*-value	<$10^{-11}$	<$10^{-11}$	0.5038	0.1156	<$10^{-11}$	<$10^{-11}$	<$10^{-11}$	<$10^{-11}$	0.0001
	Rank	11	13	3	6	10	17	9	14	7
F27	Mean	$3.30\times 10^{3}$	$3.31\times 10^{3}$	$3.28\times 10^{3}$	$3.27\times 10^{3}$	$3.28\times 10^{3}$	$4.90\times 10^{3}$	$3.42\times 10^{3}$	$3.41\times 10^{3}$	$3.33\times 10^{3}$
	Std	$5.24\times 10^{1}$	$4.87\times 10^{1}$	$9.61\times 10^{0}$	$2.11\times 10^{1}$	$4.88\times 10^{1}$	$6.23\times 10^{602}$	$9.75\times 10^{1}$	$5.26\times 10^{1}$	$5.40\times 10^{1}$
	*p*-value	<$10^{-11}$	<$10^{-11}$	<$10^{-11}$	0.0001	0.0008	<$10^{-11}$	<$10^{-11}$	<$10^{-11}$	<$10^{-11}$
	Rank	7	8	4	1	4	17	12	11	10
F28	Mean	$3.31\times 10^{3}$	$3.41\times 10^{3}$	$3.41\times 10^{3}$	$3.44\times 10^{3}$	$3.58\times 10^{3}$	$1.39\times 10^{4}$	$3.33\times 10^{3}$	$5.01\times 10^{3}$	$3.51\times 10^{3}$
	Std	$3.71\times 10^{1}$	$7.56\times 10^{1}$	$2.33\times 10^{1}$	$4.66\times 10^{1}$	$1.37\times 10^{2}$	$7.83\times 10^{3}$	$4.68\times 10^{1}$	$8.36\times 10^{802}$	$1.19\times 10^{2}$
	*p*-value	0.2452	<$10^{-11}$	<$10^{-11}$	<$10^{-11}$	<$10^{-11}$	<$10^{-11}$	0.0087	<$10^{-11}$	<$10^{-11}$
	Rank	1	6	7	8	10	17	4	15	11
F29	Mean	$4.28\times 10^{3}$	$4.38\times 10^{3}$	$4.48\times 10^{3}$	$4.04\times 10^{3}$	$4.71\times 10^{3}$	$5.92\times 10^{4}$	$4.74\times 10^{3}$	$5.48\times 10^{3}$	$4.50\times 10^{3}$
	Std	$2.88\times 10^{302}$	$3.63\times 10^{302}$	$1.67\times 10^{2}$	$2.02\times 10^{2}$	$3.61\times 10^{302}$	$9.13\times 10^{4}$	$3.84\times 10^{302}$	$5.50\times 10^{302}$	$3.73\times 10^{302}$
	*p*-value	0.0002	<$10^{-11}$	<$10^{-11}$	0.1714	<$10^{-11}$	<$10^{-11}$	<$10^{-11}$	<$10^{-11}$	<$10^{-11}$
	Rank	3	7	8	3	13	17	14	16	9
F30	Mean	$2.54\times 10^{4}$	$8.02\times 10^{6}$	$2.66\times 10^{7}$	$1.36\times 10^{7}$	$3.41\times 10^{7}$	$5.88\times 10^{9}$	$1.17\times 10^{7}$	$1.68\times 10^{8}$	$2.62\times 10^{5}$
	Std	$1.95\times 10^{4}$	$9.48\times 10^{6}$	$7.76\times 10^{6}$	$8.74\times 10^{6}$	$2.91\times 10^{7}$	$1.03\times 10^{10}$	$9.29\times 10^{6}$	$2.05\times 10^{9}$	$7.67\times 10^{8}$
	*p*-value	0.0014	<$10^{-11}$	<$10^{-11}$	<$10^{-11}$	<$10^{-11}$	<$10^{-11}$	<$10^{-11}$	<$10^{-11}$	0.0003
	Rank	3	10	15	13	16	17	12	18	6
+/≈/−		17/6/6	29/0/0	26/2/1	23/4/2	28/1/0	29/0/0	29/0/0	29/0/0	27/2/0
Mean rank		5.34	9.34	9.00	5.21	12.69	17.21	7.34	14.83	9.66
Final ranking		5	11	9	3	15	17	8	16	13

**Table A5:** Comparison of HALA and other algorithms on the CEC2017 benchmark suite (Dim = 50) (excluding F2)—Part 1: HALA vs. ALA, LSHADE, LSHADE-SPACMA, AOOA, BAEO, BPBO, CCO, CEO.

Function	Metric	HALA	ALA	LSHADE	LSHADE- SPACMA	AOOA	BAEO	BPBO	CCO	CEO
F1	Mean	$5.50\times 10^{6}$	$2.67\times 10^{6}$	$3.68\times 10^{9}$	$4.48\times 10^{9}$	$1.73\times 10^{9}$	$1.28\times 10^{11}$	$3.50\times 10^{8}$	$2.94\times 10^{7}$	$3.49\times 10^{7}$
	Std	$4.78\times 10^{6}$	$2.20\times 10^{6}$	$1.85\times 10^{9}$	$2.46\times 10^{9}$	$5.68\times 10^{8}$	$1.84\times 10^{10}$	$1.46\times 10^{8}$	$9.10\times 10^{7}$	$2.46\times 10^{7}$
	*p*-value	–	0.0157	<$10^{-11}$	<$10^{-11}$	<$10^{-11}$	<$10^{-11}$	<$10^{-11}$	0.0125	<$10^{-11}$
	Rank	4	3	11	12	9	18	8	6	7
F3	Mean	$5.12\times 10^{4}$	$4.12\times 10^{4}$	$1.09\times 10^{5}$	$1.12\times 10^{5}$	$8.96\times 10^{4}$	$1.27\times 10^{8}$	$4.22\times 10^{4}$	$8.70\times 10^{4}$	$3.98\times 10^{4}$
	Std	$1.65\times 10^{4}$	$1.30\times 10^{4}$	$3.73\times 10^{4}$	$2.55\times 10^{4}$	$2.39\times 10^{4}$	$6.44\times 10^{8}$	$1.26\times 10^{4}$	$2.83\times 10^{4}$	$1.02\times 10^{4}$
	*p*-value	–	0.0285	<$10^{-11}$	<$10^{-11}$	<$10^{-11}$	<$10^{-11}$	0.0545	<$10^{-11}$	0.0041
	Rank	6	3	12	13	10	18	5	9	2
F4	Mean	$5.34\times 10^{2}$	$5.21\times 10^{2}$	$8.08\times 10^{2}$	$8.97\times 10^{2}$	$7.41\times 10^{2}$	$3.82\times 10^{4}$	$5.62\times 10^{2}$	$6.10\times 10^{2}$	$5.30\times 10^{2}$
	Std	$3.83\times 10^{1}$	$4.45\times 10^{1}$	$1.79\times 10^{2}$	$1.95\times 10^{2}$	$1.12\times 10^{2}$	$9.34\times 10^{3}$	$3.77\times 10^{1}$	$1.21\times 10^{2}$	$4.94\times 10^{1}$
	*p*-value	–	0.0387	<$10^{-11}$	<$10^{-11}$	<$10^{-11}$	<$10^{-11}$	0.0028	0.002	0.544
	Rank	4	2	13	15	11	18	7	9	3
F5	Mean	$5.99\times 10^{2}$	$6.13\times 10^{2}$	$6.24\times 10^{2}$	$6.29\times 10^{2}$	$7.69\times 10^{2}$	$1.11\times 10^{3}$	$6.22\times 10^{2}$	$7.95\times 10^{2}$	$7.17\times 10^{2}$
	Std	$2.51\times 10^{1}$	$3.43\times 10^{1}$	$1.89\times 10^{1}$	$2.12\times 10^{1}$	$5.26\times 10^{1}$	$3.54\times 10^{1}$	$3.08\times 10^{1}$	$6.18\times 10^{1}$	$2.74\times 10^{1}$
	*p*-value	–	0.2536	0.0005	<$10^{-11}$	<$10^{-11}$	<$10^{-11}$	0.0018	<$10^{-11}$	<$10^{-11}$
	Rank	1	3	6	7	13	18	5	15	11
F6	Mean	$6.16\times 10^{2}$	$6.14\times 10^{2}$	$6.11\times 10^{2}$	$6.12\times 10^{2}$	$6.60\times 10^{2}$	$7.19\times 10^{2}$	$6.32\times 10^{2}$	$6.63\times 10^{2}$	$6.69\times 10^{2}$
	Std	$8.77\times 10^{0}$	$5.73\times 10^{0}$	$7.10\times 10^{0}$	$5.20\times 10^{0}$	$1.18\times 10^{1}$	$9.65\times 10^{0}$	$9.75\times 10^{0}$	$1.16\times 10^{1}$	$5.69\times 10^{0}$
	*p*-value	–	0.3493	0.0196	0.0571	<$10^{-11}$	<$10^{-11}$	<$10^{-11}$	<$10^{-11}$	<$10^{-11}$
	Rank	5	4	1	2	12	18	9	13	15
F7	Mean	$9.51\times 10^{2}$	$8.96\times 10^{2}$	$9.05\times 10^{2}$	$9.09\times 10^{2}$	$1.33\times 10^{3}$	$3.48\times 10^{3}$	$9.16\times 10^{2}$	$1.65\times 10^{3}$	$1.40\times 10^{3}$
	Std	$5.35\times 10^{1}$	$4.70\times 10^{1}$	$3.51\times 10^{1}$	$2.93\times 10^{1}$	$2.08\times 10^{2}$	$2.82\times 10^{2}$	$4.08\times 10^{1}$	$2.37\times 10^{2}$	$1.29\times 10^{2}$
	*p*-value	–	0.0005	0.0013	0.0014	<$10^{-11}$	<$10^{-11}$	0.0125	<$10^{-11}$	<$10^{-11}$
	Rank	5	2	3	4	10	18	6	16	12
F8	Mean	$9.02\times 10^{2}$	$9.08\times 10^{2}$	$9.26\times 10^{2}$	$9.30\times 10^{2}$	$1.07\times 10^{3}$	$1.44\times 10^{3}$	$9.31\times 10^{2}$	$1.10\times 10^{3}$	$1.10\times 10^{3}$
	Std	$2.90\times 10^{1}$	$3.80\times 10^{1}$	$2.49\times 10^{1}$	$2.18\times 10^{1}$	$4.37\times 10^{1}$	$4.84\times 10^{1}$	$2.81\times 10^{1}$	$6.03\times 10^{1}$	$4.28\times 10^{1}$
	*p*-value	–	0.517	0.0021	0.0015	<$10^{-11}$	<$10^{-11}$	0.0008	<$10^{-11}$	<$10^{-11}$
	Rank	2	3	5	6	10	18	7	12	12
F9	Mean	$3.08\times 10^{3}$	$2.26\times 10^{3}$	$3.06\times 10^{3}$	$3.28\times 10^{3}$	$1.13\times 10^{4}$	$1.44\times 10^{4}$	$3.46\times 10^{3}$	$1.04\times 10^{4}$	$9.05\times 10^{3}$
	Std	$9.14\times 10^{2}$	$8.26\times 10^{2}$	$1.54\times 10^{3}$	$1.33\times 10^{3}$	$3.54\times 10^{3}$	$1.23\times 10^{3}$	$1.12\times 10^{3}$	$2.49\times 10^{3}$	$1.24\times 10^{3}$
	*p*-value	–	0.0012	0.8612	0.6435	<$10^{-11}$	<$10^{-11}$	0.1156	<$10^{-11}$	<$10^{-11}$
	Rank	4	2	3	5	14	16	6	13	11
F10	Mean	$4.89\times 10^{3}$	$6.38\times 10^{3}$	$5.82\times 10^{3}$	$6.08\times 10^{3}$	$6.69\times 10^{3}$	$8.37\times 10^{3}$	$6.28\times 10^{3}$	$5.27\times 10^{3}$	$6.58\times 10^{3}$
	Std	$6.40\times 10^{2}$	$8.44\times 10^{2}$	$7.27\times 10^{2}$	$7.58\times 10^{2}$	$9.71\times 10^{2}$	$2.22\times 10^{2}$	$8.00\times 10^{2}$	$8.55\times 10^{2}$	$9.31\times 10^{2}$
	*p*-value	–	<$10^{-11}$	<$10^{-11}$	<$10^{-11}$	<$10^{-11}$	<$10^{-11}$	<$10^{-11}$	0.1156	<$10^{-11}$
	Rank	1	9	4	6	11	17	8	3	10
F11	Mean	$1.32\times 10^{3}$	$1.27\times 10^{3}$	$2.48\times 10^{3}$	$3.01\times 10^{3}$	$1.99\times 10^{3}$	$1.03\times 10^{5}$	$1.46\times 10^{3}$	$1.49\times 10^{3}$	$1.37\times 10^{3}$
	Std	$7.89\times 10^{1}$	$5.73\times 10^{1}$	$7.44\times 10^{2}$	$1.23\times 10^{3}$	$4.16\times 10^{2}$	$1.36\times 10^{5}$	$1.08\times 10^{2}$	$1.65\times 10^{2}$	$1.12\times 10^{2}$
	*p*-value	–	0.006	<$10^{-11}$	<$10^{-11}$	<$10^{-11}$	<$10^{-11}$	0.0001	<$10^{-11}$	0.1359
	Rank	3	2	10	13	9	18	5	6	4
F12	Mean	$3.29\times 10^{6}$	$6.62\times 10^{6}$	$1.34\times 10^{8}$	$1.88\times 10^{8}$	$3.18\times 10^{8}$	$3.09\times 10^{10}$	$7.26\times 10^{6}$	$1.98\times 10^{7}$	$1.35\times 10^{7}$
	Std	$3.24\times 10^{6}$	$5.89\times 10^{6}$	$1.14\times 10^{8}$	$1.83\times 10^{8}$	$2.58\times 10^{8}$	$5.96\times 10^{9}$	$6.85\times 10^{6}$	$2.24\times 10^{7}$	$9.26\times 10^{6}$
	*p*-value	–	0.0039	<$10^{-11}$	<$10^{-11}$	<$10^{-11}$	<$10^{-11}$	0.0117	<$10^{-11}$	<$10^{-11}$
	Rank	1	2	10	12	14	18	3	7	6
F13	Mean	$3.10\times 10^{4}$	$3.97\times 10^{4}$	$8.33\times 10^{4}$	$6.04\times 10^{6}$	$1.06\times 10^{7}$	$3.20\times 10^{10}$	$5.98\times 10^{4}$	$1.13\times 10^{5}$	$1.15\times 10^{6}$
	Std	$3.91\times 10^{4}$	$3.04\times 10^{4}$	$6.25\times 10^{4}$	$2.80\times 10^{7}$	$9.01\times 10^{6}$	$1.09\times 10^{10}$	$2.94\times 10^{4}$	$1.03\times 10^{5}$	$1.39\times 10^{6}$
	*p*-value	–	0.0859	0.0001	<$10^{-11}$	<$10^{-11}$	<$10^{-11}$	0.0016	0.0002	<$10^{-11}$
	Rank	1	2	3	9	11	18	4	5	8
F14	Mean	$2.36\times 10^{3}$	$1.85\times 10^{3}$	$8.67\times 10^{4}$	$1.61\times 10^{5}$	$5.49\times 10^{5}$	$7.78\times 10^{7}$	$6.80\times 10^{3}$	$1.77\times 10^{5}$	$6.54\times 10^{4}$
	Std	$1.80\times 10^{3}$	$3.19\times 10^{2}$	$1.05\times 10^{5}$	$2.29\times 10^{5}$	$5.61\times 10^{5}$	$5.90\times 10^{7}$	$7.96\times 10^{3}$	$2.43\times 10^{5}$	$5.07\times 10^{4}$
	*p*-value	–	0.829	<$10^{-11}$	<$10^{-11}$	<$10^{-11}$	<$10^{-11}$	0.0004	<$10^{-11}$	<$10^{-11}$
	Rank	3	2	8	10	13	18	4	11	7
F15	Mean	$1.38\times 10^{4}$	$2.25\times 10^{4}$	$2.36\times 10^{4}$	$2.13\times 10^{4}$	$8.99\times 10^{4}$	$9.04\times 10^{9}$	$3.85\times 10^{4}$	$7.47\times 10^{4}$	$5.22\times 10^{4}$
	Std	$1.07\times 10^{4}$	$1.44\times 10^{4}$	$1.02\times 10^{4}$	$9.63\times 10^{3}$	$7.78\times 10^{4}$	$3.69\times 10^{9}$	$1.92\times 10^{4}$	$6.70\times 10^{4}$	$8.33\times 10^{4}$
	*p*-value	–	0.0041	0.0002	0.0018	<$10^{-11}$	<$10^{-11}$	<$10^{-11}$	<$10^{-11}$	0.0017
	Rank	1	3	4	2	10	18	5	9	7
F16	Mean	$2.52\times 10^{3}$	$2.78\times 10^{3}$	$2.72\times 10^{3}$	$2.58\times 10^{3}$	$3.54\times 10^{3}$	$9.51\times 10^{3}$	$2.85\times 10^{3}$	$3.26\times 10^{3}$	$3.39\times 10^{3}$
	Std	$2.39\times 10^{2}$	$3.05\times 10^{2}$	$2.57\times 10^{2}$	$2.10\times 10^{2}$	$4.10\times 10^{2}$	$3.07\times 10^{3}$	$3.79\times 10^{2}$	$4.35\times 10^{2}$	$4.76\times 10^{2}$
	*p*-value	–	0.0007	0.0148	0.36	<$10^{-11}$	<$10^{-11}$	0.0064	<$10^{-11}$	<$10^{-11}$
	Rank	1	5	4	3	12	18	6	9	11
F17	Mean	$2.11\times 10^{3}$	$2.22\times 10^{3}$	$2.00\times 10^{3}$	$2.01\times 10^{3}$	$2.63\times 10^{3}$	$5.22\times 10^{4}$	$2.24\times 10^{3}$	$2.77\times 10^{3}$	$2.70\times 10^{3}$
	Std	$1.47\times 10^{2}$	$2.13\times 10^{2}$	$1.22\times 10^{2}$	$1.45\times 10^{2}$	$3.23\times 10^{2}$	$5.51\times 10^{4}$	$1.61\times 10^{2}$	$3.00\times 10^{2}$	$2.89\times 10^{2}$
	*p*-value	–	0.0285	0.0472	0.0104	<$10^{-11}$	<$10^{-11}$	0.0166	<$10^{-11}$	<$10^{-11}$
	Rank	4	6	1	2	10	18	7	13	12
F18	Mean	$7.75\times 10^{4}$	$9.54\times 10^{4}$	$4.44\times 10^{5}$	$4.04\times 10^{5}$	$4.47\times 10^{6}$	$1.26\times 10^{9}$	$1.21\times 10^{5}$	$1.23\times 10^{6}$	$1.02\times 10^{6}$
	Std	$4.56\times 10^{4}$	$5.85\times 10^{4}$	$6.17\times 10^{5}$	$3.78\times 10^{5}$	$5.54\times 10^{6}$	$7.46\times 10^{8}$	$7.74\times 10^{4}$	$2.17\times 10^{6}$	$1.65\times 10^{6}$
	*p*-value	–	0.3389	0.0001	<$10^{-11}$	<$10^{-11}$	<$10^{-11}$	0.0166	<$10^{-11}$	<$10^{-11}$
	Rank	1	2	5	4	12	18	3	10	9
F19	Mean	$1.14\times 10^{4}$	$2.29\times 10^{4}$	$2.06\times 10^{5}$	$1.58\times 10^{5}$	$6.05\times 10^{6}$	$8.79\times 10^{9}$	$2.13\times 10^{4}$	$1.70\times 10^{5}$	$2.78\times 10^{5}$
	Std	$7.53\times 10^{3}$	$1.77\times 10^{4}$	$4.67\times 10^{5}$	$2.97\times 10^{5}$	$1.18\times 10^{7}$	$3.95\times 10^{9}$	$2.25\times 10^{4}$	$3.05\times 10^{5}$	$4.87\times 10^{5}$
	*p*-value	–	0.0034	<$10^{-11}$	<$10^{-11}$	<$10^{-11}$	<$10^{-11}$	0.0786	<$10^{-11}$	<$10^{-11}$
	Rank	1	3	8	7	12	18	2	6	9
F20	Mean	$2.42\times 10^{3}$	$2.56\times 10^{3}$	$2.44\times 10^{3}$	$2.40\times 10^{3}$	$2.67\times 10^{3}$	$3.24\times 10^{3}$	$2.47\times 10^{3}$	$2.84\times 10^{3}$	$3.07\times 10^{3}$
	Std	$1.56\times 10^{2}$	$1.72\times 10^{2}$	$1.23\times 10^{2}$	$1.35\times 10^{2}$	$1.74\times 10^{2}$	$1.21\times 10^{2}$	$1.53\times 10^{2}$	$2.05\times 10^{2}$	$2.40\times 10^{2}$
	*p*-value	–	0.0098	0.4779	0.9099	<$10^{-11}$	<$10^{-11}$	0.1589	<$10^{-11}$	<$10^{-11}$
	Rank	3	7	4	2	9	18	5	12	16
F21	Mean	$2.40\times 10^{3}$	$2.41\times 10^{3}$	$2.42\times 10^{3}$	$2.43\times 10^{3}$	$2.57\times 10^{3}$	$2.93\times 10^{3}$	$2.42\times 10^{3}$	$2.59\times 10^{3}$	$2.59\times 10^{3}$
	Std	$2.89\times 10^{1}$	$3.18\times 10^{1}$	$2.07\times 10^{1}$	$2.37\times 10^{1}$	$5.54\times 10^{1}$	$5.14\times 10^{1}$	$2.87\times 10^{1}$	$5.99\times 10^{1}$	$5.23\times 10^{1}$
	*p*-value	–	0.829	0.0125	0.0015	<$10^{-11}$	<$10^{-11}$	0.0243	<$10^{-11}$	<$10^{-11}$
	Rank	1	3	4	5	11	18	4	13	13
F22	Mean	$3.62\times 10^{3}$	$6.83\times 10^{3}$	$3.41\times 10^{3}$	$2.87\times 10^{3}$	$5.34\times 10^{3}$	$1.02\times 10^{4}$	$2.51\times 10^{3}$	$6.36\times 10^{3}$	$6.42\times 10^{3}$
	Std	$1.92\times 10^{3}$	$1.95\times 10^{3}$	$1.64\times 10^{3}$	$4.47\times 10^{2}$	$2.61\times 10^{3}$	$3.03\times 10^{2}$	$7.32\times 10^{1}$	$2.09\times 10^{3}$	$2.60\times 10^{3}$
	*p*-value	–	<$10^{-11}$	0.8612	0.6435	0.0044	<$10^{-11}$	0.6435	0.0001	0.0003
	Rank	5	11	4	3	8	18	2	9	10
F23	Mean	$2.77\times 10^{3}$	$2.77\times 10^{3}$	$2.81\times 10^{3}$	$2.80\times 10^{3}$	$3.03\times 10^{3}$	$3.97\times 10^{3}$	$2.78\times 10^{3}$	$3.07\times 10^{3}$	$3.33\times 10^{3}$
	Std	$2.97\times 10^{1}$	$2.96\times 10^{1}$	$2.80\times 10^{1}$	$3.22\times 10^{1}$	$1.02\times 10^{2}$	$1.65\times 10^{2}$	$3.12\times 10^{1}$	$1.19\times 10^{2}$	$1.99\times 10^{2}$
	*p*-value	–	0.4779	<$10^{-11}$	0.0006	<$10^{-11}$	<$10^{-11}$	0.0822	<$10^{-11}$	<$10^{-11}$
	Rank	2	2	7	6	11	18	4	12	16
F24	Mean	$2.92\times 10^{3}$	$2.96\times 10^{3}$	$2.97\times 10^{3}$	$2.97\times 10^{3}$	$3.14\times 10^{3}$	$4.45\times 10^{3}$	$2.95\times 10^{3}$	$3.19\times 10^{3}$	$3.49\times 10^{3}$
	Std	$2.81\times 10^{1}$	$3.87\times 10^{1}$	$3.52\times 10^{1}$	$2.48\times 10^{1}$	$7.67\times 10^{1}$	$2.92\times 10^{302}$	$2.57\times 10^{1}$	$1.05\times 10^{2}$	$1.71\times 10^{2}$
	*p*-value	–	0.0001	<$10^{-11}$	<$10^{-11}$	<$10^{-11}$	<$10^{-11}$	0.0004	<$10^{-11}$	<$10^{-11}$
	Rank	1	5	6	6	11	18	4	12	16
F25	Mean	$2.93\times 10^{3}$	$2.91\times 10^{3}$	$3.12\times 10^{3}$	$3.14\times 10^{3}$	$3.16\times 10^{3}$	$1.99\times 10^{4}$	$2.96\times 10^{3}$	$2.97\times 10^{3}$	$2.91\times 10^{3}$
	Std	$2.33\times 10^{1}$	$2.17\times 10^{1}$	$7.48\times 10^{1}$	$6.84\times 10^{1}$	$1.36\times 10^{2}$	$4.73\times 10^{3}$	$2.25\times 10^{1}$	$4.29\times 10^{1}$	$1.75\times 10^{1}$
	*p*-value	–	0.0036	<$10^{-11}$	<$10^{-11}$	<$10^{-11}$	<$10^{-11}$	0.0002	0.006	0.0002
	Rank	4	2	12	13	14	18	7	8	2
F26	Mean	$4.85\times 10^{3}$	$4.91\times 10^{3}$	$5.50\times 10^{3}$	$5.47\times 10^{3}$	$7.21\times 10^{3}$	$1.75\times 10^{4}$	$4.18\times 10^{3}$	$8.05\times 10^{3}$	$8.43\times 10^{3}$
	Std	$1.02\times 10^{3}$	$5.31\times 10^{2}$	$5.75\times 10^{2}$	$6.64\times 10^{2}$	$1.64\times 10^{3}$	$2.05\times 10^{3}$	$5.92\times 10^{2}$	$1.47\times 10^{3}$	$1.01\times 10^{3}$
	*p*-value	–	0.6143	0.0077	0.035	<$10^{-11}$	<$10^{-11}$	0.0064	<$10^{-11}$	<$10^{-11}$
	Rank	4	5	9	8	12	18	2	14	15
F27	Mean	$3.25\times 10^{3}$	$3.23\times 10^{3}$	$3.29\times 10^{3}$	$3.30\times 10^{3}$	$3.39\times 10^{3}$	$5.84\times 10^{3}$	$3.25\times 10^{3}$	$3.38\times 10^{3}$	$3.44\times 10^{3}$
	Std	$2.25\times 10^{1}$	$2.08\times 10^{1}$	$3.01\times 10^{1}$	$2.87\times 10^{1}$	$9.67\times 10^{1}$	$5.58\times 10^{2}$	$1.47\times 10^{1}$	$7.82\times 10^{1}$	$1.49\times 10^{2}$
	*p*-value	–	0.0256	<$10^{-11}$	<$10^{-11}$	<$10^{-11}$	<$10^{-11}$	0.7971	<$10^{-11}$	<$10^{-11}$
	Rank	3	2	5	6	10	18	3	9	13
F28	Mean	$3.30\times 10^{3}$	$3.80\times 10^{3}$	$3.70\times 10^{3}$	$3.81\times 10^{3}$	$3.54\times 10^{3}$	$1.48\times 10^{4}$	$3.37\times 10^{3}$	$3.60\times 10^{3}$	$3.28\times 10^{3}$
	Std	$3.17\times 10^{1}$	$1.14\times 10^{3}$	$2.33\times 10^{2}$	$2.79\times 10^{302}$	$1.26\times 10^{2}$	$2.60\times 10^{3}$	$4.21\times 10^{1}$	$5.41\times 10^{302}$	$2.67\times 10^{1}$
	*p*-value	–	0.0656	<$10^{-11}$	<$10^{-11}$	<$10^{-11}$	<$10^{-11}$	<$10^{-11}$	<$10^{-11}$	0.005
	Rank	3	12	10	13	8	18	5	9	2
F29	Mean	$3.95\times 10^{3}$	$4.01\times 10^{3}$	$4.17\times 10^{3}$	$4.18\times 10^{3}$	$4.92\times 10^{3}$	$6.64\times 10^{4}$	$4.27\times 10^{3}$	$4.81\times 10^{3}$	$4.69\times 10^{3}$
	Std	$2.05\times 10^{2}$	$2.26\times 10^{2}$	$2.62\times 10^{2}$	$2.27\times 10^{2}$	$4.14\times 10^{302}$	$1.25\times 10^{5}$	$2.41\times 10^{2}$	$4.85\times 10^{302}$	$3.64\times 10^{302}$
	*p*-value	–	0.1846	0.0012	0.0002	<$10^{-11}$	<$10^{-11}$	0.0001	<$10^{-11}$	<$10^{-11}$
	Rank	1	2	4	5	12	18	6	11	10
F30	Mean	$5.60\times 10^{4}$	$5.69\times 10^{4}$	$4.24\times 10^{6}$	$3.96\times 10^{6}$	$1.66\times 10^{7}$	$5.55\times 10^{9}$	$5.13\times 10^{5}$	$1.08\times 10^{6}$	$1.40\times 10^{6}$
	Std	$7.62\times 10^{4}$	$5.67\times 10^{4}$	$3.50\times 10^{6}$	$4.07\times 10^{6}$	$2.20\times 10^{7}$	$1.89\times 10^{9}$	$4.23\times 10^{5}$	$1.30\times 10^{6}$	$1.46\times 10^{6}$
	*p*-value	–	0.7655	<$10^{-11}$	<$10^{-11}$	<$10^{-11}$	<$10^{-11}$	<$10^{-11}$	<$10^{-11}$	<$10^{-11}$
	Rank	1	2	9	8	14	18	4	5	7
+/≈/−		–	9/12/8	23/3/3	22/5/2	29/0/0	29/0/0	20/7/2	28/1/0	24/2/3
Mean rank		2.34	3.76	6.07	6.28	11.59	17.83	4.79	9.59	8.90
Final ranking		1	2	6	7	14	18	4	12	10

**Table A6:** Comparison of HALA and other algorithms on the CEC2017 benchmark suite (Dim = 50) (excluding F2)—Part 2: HALA vs. CQALA, DFL, DMOA, DHOA, FGO, KLA, PGA, SO, SOO (continued from Table A5).

Function	Metric	CQALA	DFL	DMOA	DHOA	FGO	KLA	PGA	SO	SOO
F1	Mean	$1.66\times 10^{5}$	$1.22\times 10^{8}$	$2.14\times 10^{9}$	$5.90\times 10^{8}$	$6.40\times 10^{9}$	$1.27\times 10^{11}$	$1.71\times 10^{7}$	$2.41\times 10^{10}$	$1.65\times 10^{9}$
	Std	$3.29\times 10^{305}$	$4.02\times 10^{7}$	$2.58\times 10^{8}$	$3.84\times 10^{8}$	$2.17\times 10^{9}$	$1.78\times 10^{10}$	$3.75\times 10^{6}$	$6.48\times 10^{9}$	$1.19\times 10^{9}$
	*p*-value	<$10^{-11}$	<$10^{-11}$	<$10^{-11}$	<$10^{-11}$	<$10^{-11}$	<$10^{-11}$	<$10^{-11}$	<$10^{-11}$	<$10^{-11}$
	Rank	2	5	13	10	14	17	1	15	16
F3	Mean	$7.18\times 10^{4}$	$1.76\times 10^{5}$	$1.12\times 10^{5}$	$7.94\times 10^{4}$	$1.04\times 10^{5}$	$2.38\times 10^{10}$	$1.10\times 10^{5}$	$2.86\times 10^{5}$	$2.09\times 10^{5}$
	Std	$1.48\times 10^{4}$	$4.97\times 10^{4}$	$1.94\times 10^{4}$	$1.33\times 10^{4}$	$2.14\times 10^{4}$	$5.57\times 10^{10}$	$2.79\times 10^{4}$	$5.42\times 10^{4}$	$6.99\times 10^{4}$
	*p*-value	<$10^{-11}$	<$10^{-11}$	<$10^{-11}$	<$10^{-11}$	<$10^{-11}$	<$10^{-11}$	<$10^{-11}$	<$10^{-11}$	<$10^{-11}$
	Rank	8	15	14	11	13	17	7	16	4
F4	Mean	$5.46\times 10^{2}$	$5.81\times 10^{2}$	$6.34\times 10^{2}$	$6.10\times 10^{2}$	$9.39\times 10^{2}$	$3.44\times 10^{4}$	$5.62\times 10^{2}$	$3.11\times 10^{3}$	$6.63\times 10^{2}$
	Std	$4.73\times 10^{1}$	$6.43\times 10^{1}$	$3.75\times 10^{1}$	$2.89\times 10^{1}$	$1.93\times 10^{2}$	$9.19\times 10^{3}$	$4.06\times 10^{1}$	$9.17\times 10^{902}$	$7.56\times 10^{1}$
	*p*-value	0.3286	0.0008	<$10^{-11}$	<$10^{-11}$	<$10^{-11}$	<$10^{-11}$	0.0098	<$10^{-11}$	<$10^{-11}$
	Rank	1	5	10	8	16	17	6	14	12
F5	Mean	$6.59\times 10^{2}$	$7.39\times 10^{2}$	$7.34\times 10^{2}$	$6.21\times 10^{2}$	$7.97\times 10^{2}$	$8.73\times 10^{2}$	$7.10\times 10^{2}$	$9.15\times 10^{2}$	$6.44\times 10^{2}$
	Std	$2.65\times 10^{1}$	$6.16\times 10^{1}$	$1.10\times 10^{1}$	$2.22\times 10^{1}$	$3.21\times 10^{1}$	$3.31\times 10^{1}$	$4.38\times 10^{1}$	$4.52\times 10^{1}$	$2.78\times 10^{1}$
	*p*-value	<$10^{-11}$	<$10^{-11}$	<$10^{-11}$	0.0028	<$10^{-11}$	<$10^{-11}$	<$10^{-11}$	<$10^{-11}$	<$10^{-11}$
	Rank	10	14	12	4	16	17	9	18	8
F6	Mean	$6.46\times 10^{2}$	$6.53\times 10^{2}$	$6.20\times 10^{2}$	$6.25\times 10^{2}$	$6.45\times 10^{2}$	$6.78\times 10^{2}$	$6.55\times 10^{2}$	$6.80\times 10^{2}$	$6.42\times 10^{2}$
	Std	$6.20\times 10^{0}$	$1.69\times 10^{1}$	$1.85\times 10^{0}$	$6.16\times 10^{0}$	$1.15\times 10^{1}$	$6.86\times 10^{0}$	$1.09\times 10^{1}$	$9.17\times 10^{0}$	$8.69\times 10^{0}$
	*p*-value	<$10^{-11}$	<$10^{-11}$	0.035	0.0002	<$10^{-11}$	<$10^{-11}$	<$10^{-11}$	<$10^{-11}$	<$10^{-11}$
	Rank	11	14	6	7	10	17	15	16	3
F7	Mean	$1.17\times 10^{3}$	$1.24\times 10^{3}$	$1.03\times 10^{3}$	$1.01\times 10^{3}$	$1.17\times 10^{3}$	$2.14\times 10^{3}$	$1.14\times 10^{3}$	$1.84\times 10^{3}$	$1.10\times 10^{3}$
	Std	$8.99\times 10^{1}$	$1.29\times 10^{2}$	$1.18\times 10^{1}$	$4.44\times 10^{1}$	$8.54\times 10^{1}$	$4.52\times 10^{402}$	$9.38\times 10^{1}$	$1.74\times 10^{2}$	$6.58\times 10^{1}$
	*p*-value	<$10^{-11}$	<$10^{-11}$	<$10^{-11}$	0.0006	<$10^{-11}$	<$10^{-11}$	<$10^{-11}$	<$10^{-11}$	<$10^{-11}$
	Rank	11	13	7	4	11	17	9	15	8
F8	Mean	$1.00\times 10^{3}$	$1.03\times 10^{3}$	$1.03\times 10^{3}$	$9.39\times 10^{2}$	$1.09\times 10^{3}$	$1.22\times 10^{3}$	$9.98\times 10^{2}$	$1.17\times 10^{3}$	$9.49\times 10^{2}$
	Std	$3.77\times 10^{1}$	$5.92\times 10^{1}$	$1.44\times 10^{1}$	$2.43\times 10^{1}$	$3.54\times 10^{1}$	$4.79\times 10^{1}$	$4.58\times 10^{41}$	$3.77\times 10^{41}$	$3.33\times 10^{41}$
	*p*-value	<$10^{-11}$	<$10^{-11}$	<$10^{-11}$	0.0003	<$10^{-11}$	<$10^{-11}$	<$10^{-11}$	<$10^{-11}$	<$10^{-11}$
	Rank	8	10	10	1	14	17	4	15	9
F9	Mean	$5.05\times 10^{3}$	$1.18\times 10^{4}$	$1.75\times 10^{3}$	$2.60\times 10^{3}$	$8.92\times 10^{3}$	$3.52\times 10^{4}$	$9.39\times 10^{3}$	$1.79\times 10^{4}$	$5.38\times 10^{3}$
	Std	$4.95\times 10^{2}$	$4.96\times 10^{3}$	$2.31\times 10^{2}$	$6.84\times 10^{602}$	$4.56\times 10^{3}$	$1.04\times 10^{4}$	$3.10\times 10^{3}$	$4.66\times 10^{3}$	$1.65\times 10^{3}$
	*p*-value	<$10^{-11}$	<$10^{-11}$	0.8612	0.0449	<$10^{-11}$	<$10^{-11}$	<$10^{-11}$	<$10^{-11}$	<$10^{-11}$
	Rank	7	15	1	3	10	18	12	17	8
F10	Mean	$5.01\times 10^{3}$	$7.08\times 10^{3}$	$8.55\times 10^{3}$	$5.59\times 10^{3}$	$8.69\times 10^{3}$	$1.00\times 10^{4}$	$5.51\times 10^{3}$	$8.53\times 10^{3}$	$6.24\times 10^{3}$
	Std	$1.08\times 10^{3}$	$6.61\times 10^{602}$	$2.33\times 10^{2}$	$7.71\times 10^{602}$	$6.92\times 10^{602}$	$4.80\times 10^{402}$	$5.17\times 10^{502}$	$4.62\times 10^{402}$	$5.91\times 10^{502}$
	*p*-value	0.9099	<$10^{-11}$	<$10^{-11}$	0.0024	<$10^{-11}$	<$10^{-11}$	0.0024	<$10^{-11}$	<$10^{-11}$
	Rank	2	12	15	5	16	18	4	14	7
F11	Mean	$1.29\times 10^{3}$	$1.53\times 10^{3}$	$2.17\times 10^{3}$	$1.39\times 10^{3}$	$2.26\times 10^{3}$	$2.56\times 10^{5}$	$1.49\times 10^{3}$	$1.29\times 10^{4}$	$3.08\times 10^{3}$
	Std	$6.52\times 10^{1}$	$1.52\times 10^{2}$	$2.22\times 10^{202}$	$6.75\times 10^{1}$	$4.30\times 10^{402}$	$7.11\times 10^{5}$	$1.11\times 10^{2}$	$4.06\times 10^{3}$	$2.00\times 10^{3}$
	*p*-value	0.1204	<$10^{-11}$	<$10^{-11}$	0.0016	<$10^{-11}$	<$10^{-11}$	<$10^{-11}$	<$10^{-11}$	<$10^{-11}$
	Rank	1	7	11	5	10	17	6	16	15
F12	Mean	$4.86\times 10^{6}$	$1.44\times 10^{8}$	$3.46\times 10^{8}$	$2.05\times 10^{8}$	$7.02\times 10^{8}$	$3.18\times 10^{10}$	$1.10\times 10^{8}$	$1.73\times 10^{9}$	$2.48\times 10^{7}$
	Std	$5.84\times 10^{6}$	$1.30\times 10^{8}$	$7.23\times 10^{7}$	$8.82\times 10^{7}$	$4.52\times 10^{8}$	$9.46\times 10^{9}$	$5.47\times 10^{7}$	$6.89\times 10^{8}$	$4.05\times 10^{7}$
	*p*-value	0.544	<$10^{-11}$	<$10^{-11}$	<$10^{-11}$	<$10^{-11}$	<$10^{-11}$	<$10^{-11}$	<$10^{-11}$	0.0001
	Rank	4	11	15	13	16	17	9	18	5
F13	Mean	$1.93\times 10^{4}$	$9.68\times 10^{5}$	$1.17\times 10^{8}$	$5.05\times 10^{5}$	$2.27\times 10^{8}$	$3.27\times 10^{10}$	$2.82\times 10^{5}$	$1.39\times 10^{9}$	$3.90\times 10^{5}$
	Std	$5.72\times 10^{4}$	$1.93\times 10^{6}$	$3.45\times 10^{7}$	$8.35\times 10^{5}$	$1.81\times 10^{8}$	$9.10\times 10^{9}$	$1.15\times 10^{5}$	$8.41\times 10^{8}$	$1.00\times 10^{6}$
	*p*-value	0.0117	<$10^{-11}$	<$10^{-11}$	<$10^{-11}$	<$10^{-11}$	<$10^{-11}$	<$10^{-11}$	<$10^{-11}$	0.0008
	Rank	2	7	13	6	12	17	10	16	15
F14	Mean	$2.63\times 10^{5}$	$5.14\times 10^{4}$	$3.29\times 10^{305}$	$1.57\times 10^{5}$	$3.37\times 10^{305}$	$7.77\times 10^{7}$	$2.35\times 10^{205}$	$1.38\times 10^{6}$	$5.02\times 10^{5}$
	Std	$1.77\times 10^{5}$	$5.04\times 10^{4}$	$2.03\times 10^{205}$	$1.18\times 10^{105}$	$2.95\times 10^{205}$	$1.33\times 10^{8}$	$2.15\times 10^{205}$	$1.35\times 10^{6}$	$8.40\times 10^{5}$
	*p*-value	<$10^{-11}$	<$10^{-11}$	<$10^{-11}$	<$10^{-11}$	<$10^{-11}$	<$10^{-11}$	<$10^{-11}$	<$10^{-11}$	<$10^{-11}$
	Rank	12	1	14	9	15	17	6	16	13
F15	Mean	$3.25\times 10^{3}$	$8.46\times 10^{4}$	$1.58\times 10^{7}$	$4.31\times 10^{5}$	$2.64\times 10^{7}$	$7.14\times 10^{9}$	$7.51\times 10^{4}$	$1.28\times 10^{8}$	$1.62\times 10^{4}$
	Std	$1.55\times 10^{3}$	$6.03\times 10^{4}$	$6.78\times 10^{6}$	$6.04\times 10^{5}$	$3.17\times 10^{7}$	$3.79\times 10^{9}$	$3.38\times 10^{4}$	$9.09\times 10^{7}$	$1.41\times 10^{4}$
	*p*-value	<$10^{-11}$	<$10^{-11}$	<$10^{-11}$	<$10^{-11}$	<$10^{-11}$	<$10^{-11}$	<$10^{-11}$	<$10^{-11}$	0.7655
	Rank	3	8	14	11	15	17	6	16	4
F16	Mean	$3.06\times 10^{3}$	$3.12\times 10^{3}$	$3.52\times 10^{3}$	$2.68\times 10^{3}$	$3.81\times 10^{3}$	$7.67\times 10^{3}$	$3.16\times 10^{3}$	$4.29\times 10^{3}$	$3.08\times 10^{3}$
	Std	$3.26\times 10^{302}$	$4.20\times 10^{302}$	$2.31\times 10^{202}$	$2.24\times 10^{202}$	$3.27\times 10^{302}$	$2.58\times 10^{3}$	$3.36\times 10^{302}$	$3.12\times 10^{302}$	$4.14\times 10^{302}$
	*p*-value	<$10^{-11}$	<$10^{-11}$	<$10^{-11}$	0.0157	<$10^{-11}$	<$10^{-11}$	<$10^{-11}$	<$10^{-11}$	<$10^{-11}$
	Rank	7	8	13	2	14	17	10	16	15
F17	Mean	$2.50\times 10^{3}$	$2.53\times 10^{3}$	$2.30\times 10^{3}$	$2.02\times 10^{3}$	$2.65\times 10^{3}$	$8.55\times 10^{4}$	$2.38\times 10^{3}$	$3.24\times 10^{3}$	$2.49\times 10^{3}$
	Std	$2.65\times 10^{202}$	$2.84\times 10^{202}$	$1.19\times 10^{102}$	$1.54\times 10^{102}$	$2.52\times 10^{202}$	$1.43\times 10^{5}$	$2.55\times 10^{202}$	$2.53\times 10^{202}$	$3.02\times 10^{202}$
	*p*-value	<$10^{-11}$	<$10^{-11}$	<$10^{-11}$	0.03	<$10^{-11}$	<$10^{-11}$	0.0002	<$10^{-11}$	<$10^{-11}$
	Rank	8	9	5	3	11	17	5	15	7
F18	Mean	$2.87\times 10^{5}$	$1.18\times 10^{6}$	$2.49\times 10^{6}$	$5.79\times 10^{5}$	$5.89\times 10^{6}$	$8.29\times 10^{8}$	$2.93\times 10^{6}$	$1.74\times 10^{7}$	$1.75\times 10^{6}$
	Std	$3.94\times 10^{305}$	$1.22\times 10^{6}$	$1.37\times 10^{6}$	$4.72\times 10^{405}$	$3.99\times 10^{6}$	$5.41\times 10^{8}$	$2.62\times 10^{6}$	$1.08\times 10^{7}$	$2.41\times 10^{6}$
	*p*-value	0.0005	<$10^{-11}$	<$10^{-11}$	<$10^{-11}$	<$10^{-11}$	<$10^{-11}$	<$10^{-11}$	<$10^{-11}$	<$10^{-11}$
	Rank	4	7	11	6	13	17	12	16	8
F19	Mean	$6.17\times 10^{3}$	$2.70\times 10^{5}$	$2.65\times 10^{7}$	$1.19\times 10^{6}$	$4.93\times 10^{7}$	$1.14\times 10^{10}$	$6.19\times 10^{6}$	$1.68\times 10^{8}$	$2.08\times 10^{4}$
	Std	$4.74\times 10^{3}$	$4.46\times 10^{405}$	$1.15\times 10^{7}$	$1.28\times 10^{6}$	$4.64\times 10^{7}$	$4.75\times 10^{9}$	$4.98\times 10^{6}$	$1.00\times 10^{8}$	$3.09\times 10^{4}$
	*p*-value	0.005	<$10^{-11}$	<$10^{-11}$	<$10^{-11}$	<$10^{-11}$	<$10^{-11}$	<$10^{-11}$	<$10^{-11}$	0.5038
	Rank	3	10	13	14	15	17	11	16	12
F20	Mean	$2.62\times 10^{3}$	$2.80\times 10^{3}$	$2.74\times 10^{3}$	$2.43\times 10^{3}$	$2.84\times 10^{3}$	$3.43\times 10^{3}$	$2.55\times 10^{3}$	$3.06\times 10^{3}$	$2.76\times 10^{3}$
	Std	$2.49\times 10^{202}$	$2.42\times 10^{202}$	$1.26\times 10^{102}$	$1.16\times 10^{102}$	$2.03\times 10^{202}$	$2.38\times 10^{202}$	$1.48\times 10^{102}$	$1.76\times 10^{102}$	$2.60\times 10^{202}$
	*p*-value	0.0018	<$10^{-11}$	<$10^{-11}$	0.9099	<$10^{-11}$	<$10^{-11}$	0.0002	<$10^{-11}$	<$10^{-11}$
	Rank	8	11	10	1	13	17	6	15	14
F21	Mean	$2.48\times 10^{3}$	$2.54\times 10^{3}$	$2.52\times 10^{3}$	$2.42\times 10^{3}$	$2.57\times 10^{3}$	$2.76\times 10^{3}$	$2.50\times 10^{3}$	$2.66\times 10^{3}$	$2.45\times 10^{3}$
	Std	$4.25\times 10^{41}$	$4.58\times 10^{41}$	$1.27\times 10^{1}$	$1.97\times 10^{1}$	$3.01\times 10^{1}$	$6.69\times 10^{61}$	$4.61\times 10^{41}$	$3.08\times 10^{31}$	$4.51\times 10^{41}$
	*p*-value	<$10^{-11}$	<$10^{-11}$	<$10^{-11}$	0.03	<$10^{-11}$	<$10^{-11}$	<$10^{-11}$	<$10^{-11}$	0.0004
	Rank	7	10	9	2	12	17	8	15	6
F22	Mean	$3.42\times 10^{3}$	$8.09\times 10^{3}$	$2.64\times 10^{3}$	$2.44\times 10^{3}$	$4.15\times 10^{3}$	$1.14\times 10^{4}$	$6.27\times 10^{3}$	$9.88\times 10^{3}$	$6.18\times 10^{3}$
	Std	$1.96\times 10^{3}$	$1.61\times 10^{3}$	$3.01\times 10^{1}$	$6.12\times 10^{1}$	$2.40\times 10^{3}$	$6.95\times 10^{602}$	$1.93\times 10^{3}$	$9.60\times 10^{902}$	$1.92\times 10^{3}$
	*p*-value	0.7343	<$10^{-11}$	0.6435	0.6435	0.221	<$10^{-11}$	<$10^{-11}$	<$10^{-11}$	<$10^{-11}$
	Rank	6	13	7	1	7	17	12	15	11
F23	Mean	$2.92\times 10^{3}$	$2.96\times 10^{3}$	$2.88\times 10^{3}$	$2.79\times 10^{3}$	$2.94\times 10^{3}$	$3.59\times 10^{3}$	$2.92\times 10^{3}$	$3.03\times 10^{3}$	$2.87\times 10^{3}$
	Std	$9.06\times 10^{1}$	$9.29\times 10^{91}$	$1.62\times 10^{1}$	$2.11\times 10^{1}$	$5.32\times 10^{51}$	$1.88\times 10^{102}$	$6.63\times 10^{61}$	$4.46\times 10^{41}$	$4.85\times 10^{41}$
	*p*-value	<$10^{-11}$	<$10^{-11}$	<$10^{-11}$	0.0024	<$10^{-11}$	<$10^{-11}$	<$10^{-11}$	<$10^{-11}$	<$10^{-11}$
	Rank	9	10	5	3	8	17	9	13	14
F24	Mean	$3.11\times 10^{3}$	$3.09\times 10^{3}$	$3.03\times 10^{3}$	$2.94\times 10^{3}$	$3.08\times 10^{3}$	$3.81\times 10^{3}$	$3.03\times 10^{3}$	$3.14\times 10^{3}$	$3.02\times 10^{3}$
	Std	$6.07\times 10^{61}$	$6.61\times 10^{61}$	$1.39\times 10^{1}$	$2.09\times 10^{21}$	$2.55\times 10^{21}$	$2.51\times 10^{202}$	$6.79\times 10^{61}$	$3.69\times 10^{31}$	$6.49\times 10^{61}$
	*p*-value	<$10^{-11}$	<$10^{-11}$	<$10^{-11}$	0.0003	<$10^{-11}$	<$10^{-11}$	<$10^{-11}$	<$10^{-11}$	<$10^{-11}$
	Rank	13	10	8	3	9	17	8	14	15
F25	Mean	$2.92\times 10^{3}$	$3.01\times 10^{3}$	$3.05\times 10^{3}$	$3.04\times 10^{3}$	$3.28\times 10^{3}$	$1.93\times 10^{4}$	$2.97\times 10^{3}$	$5.93\times 10^{3}$	$3.05\times 10^{3}$
	Std	$2.18\times 10^{21}$	$6.69\times 10^{61}$	$2.24\times 10^{21}$	$3.97\times 10^{41}$	$1.60\times 10^{102}$	$4.58\times 10^{3}$	$4.77\times 10^{41}$	$1.35\times 10^{3}$	$5.33\times 10^{51}$
	*p*-value	0.0117	<$10^{-11}$	<$10^{-11}$	<$10^{-11}$	<$10^{-11}$	<$10^{-11}$	0.0013	<$10^{-11}$	<$10^{-11}$
	Rank	3	9	11	10	15	17	6	16	11
F26	Mean	$7.03\times 10^{3}$	$7.17\times 10^{3}$	$4.74\times 10^{3}$	$5.46\times 10^{3}$	$6.49\times 10^{3}$	$1.53\times 10^{4}$	$6.76\times 10^{3}$	$7.52\times 10^{3}$	$5.88\times 10^{3}$
	Std	$1.06\times 10^{3}$	$1.25\times 10^{3}$	$1.21\times 10^{3}$	$1.48\times 10^{3}$	$5.10\times 10^{502}$	$2.01\times 10^{3}$	$9.43\times 10^{902}$	$4.77\times 10^{402}$	$7.34\times 10^{702}$
	*p*-value	<$10^{-11}$	<$10^{-11}$	0.5038	0.1156	<$10^{-11}$	<$10^{-11}$	<$10^{-11}$	<$10^{-11}$	0.0001
	Rank	11	13	3	6	10	17	9	14	7
F27	Mean	$3.30\times 10^{3}$	$3.31\times 10^{3}$	$3.28\times 10^{3}$	$3.27\times 10^{3}$	$3.28\times 10^{3}$	$4.90\times 10^{3}$	$3.42\times 10^{3}$	$3.41\times 10^{3}$	$3.33\times 10^{3}$
	Std	$5.24\times 10^{51}$	$4.87\times 10^{41}$	$9.61\times 10^{0}$	$2.11\times 10^{21}$	$4.88\times 10^{41}$	$6.23\times 10^{602}$	$9.75\times 10^{91}$	$5.26\times 10^{51}$	$5.40\times 10^{51}$
	*p*-value	<$10^{-11}$	<$10^{-11}$	<$10^{-11}$	0.0001	0.0008	<$10^{-11}$	<$10^{-11}$	<$10^{-11}$	<$10^{-11}$
	Rank	7	8	4	1	4	17	12	11	10
F28	Mean	$3.31\times 10^{3}$	$3.41\times 10^{3}$	$3.41\times 10^{3}$	$3.44\times 10^{3}$	$3.58\times 10^{3}$	$1.39\times 10^{4}$	$3.33\times 10^{3}$	$5.01\times 10^{3}$	$3.51\times 10^{3}$
	Std	$3.71\times 10^{41}$	$7.56\times 10^{71}$	$2.33\times 10^{21}$	$4.66\times 10^{41}$	$1.37\times 10^{102}$	$2.14\times 10^{3}$	$4.68\times 10^{41}$	$8.36\times 10^{802}$	$1.19\times 10^{102}$
	*p*-value	0.2452	<$10^{-11}$	<$10^{-11}$	<$10^{-11}$	<$10^{-11}$	<$10^{-11}$	0.0087	<$10^{-11}$	<$10^{-11}$
	Rank	1	6	7	8	10	17	4	15	11
F29	Mean	$4.28\times 10^{3}$	$4.38\times 10^{3}$	$4.48\times 10^{3}$	$4.04\times 10^{3}$	$4.71\times 10^{3}$	$5.92\times 10^{4}$	$4.74\times 10^{3}$	$5.48\times 10^{3}$	$4.50\times 10^{3}$
	Std	$2.88\times 10^{302}$	$3.63\times 10^{302}$	$1.67\times 10^{102}$	$2.02\times 10^{202}$	$3.61\times 10^{302}$	$9.13\times 10^{4}$	$3.84\times 10^{302}$	$5.50\times 10^{302}$	$3.73\times 10^{302}$
	*p*-value	0.0002	<$10^{-11}$	<$10^{-11}$	0.1714	<$10^{-11}$	<$10^{-11}$	<$10^{-11}$	<$10^{-11}$	<$10^{-11}$
	Rank	3	7	8	3	13	17	14	16	9
F30	Mean	$2.54\times 10^{4}$	$8.02\times 10^{6}$	$2.66\times 10^{7}$	$1.36\times 10^{7}$	$3.41\times 10^{7}$	$5.88\times 10^{9}$	$1.17\times 10^{7}$	$1.68\times 10^{8}$	$2.62\times 10^{5}$
	Std	$1.95\times 10^{4}$	$9.48\times 10^{6}$	$7.76\times 10^{6}$	$8.74\times 10^{6}$	$2.91\times 10^{7}$	$3.08\times 10^{9}$	$9.29\times 10^{6}$	$9.75\times 10^{7}$	$3.30\times 10^{5}$
	*p*-value	0.0014	<$10^{-11}$	<$10^{-11}$	<$10^{-11}$	<$10^{-11}$	<$10^{-11}$	<$10^{-11}$	<$10^{-11}$	0.0003
	Rank	3	10	15	13	16	17	12	18	6
+/≈/−		17/6/6	29/0/0	26/2/1	23/4/2	28/1/0	29/0/0	29/0/0	29/0/0	27/2/0
Mean rank		5.31	9.31	9.00	5.17	12.66	17.21	7.31	14.83	9.62
Final ranking		5	11	9	3	15	17	8	16	13

**Table A7:** Comparison of HALA and other algorithms on the CEC2017 benchmark suite (Dim = 100) (excluding F2)—Part 1: HALA vs. ALA, LSHADE, LSHADE-SPACMA, AOOA, BAEO, BPBO, CCO, CEO.

Function	Metric	HALA	ALA	LSHADE	LSHADE- SPACMA	AOOA	BAEO	BPBO	CCO	CEO
F1	Mean	$2.63\times 10^{10}$	$2.18\times 10^{10}$	$1.15\times 10^{11}$	$1.12\times 10^{11}$	$8.12\times 10^{10}$	$6.67\times 10^{11}$	$3.31\times 10^{10}$	$5.11\times 10^{10}$	$1.19\times 10^{9}$
	Std	$7.74\times 10^{9}$	$4.85\times 10^{9}$	$1.92\times 10^{10}$	$1.73\times 10^{10}$	$1.26\times 10^{10}$	$3.70\times 10^{10}$	$6.10\times 10^{9}$	$1.29\times 10^{10}$	$1.81\times 10^{8}$
	*p*-value	–	0.0387	<$10^{-11}$	<$10^{-11}$	<$10^{-11}$	<$10^{-11}$	0.0024	<$10^{-11}$	<$10^{-11}$
	Rank	5	4	13	12	10	18	7	9	3
F3	Mean	$4.27\times 10^{5}$	$3.63\times 10^{5}$	$5.62\times 10^{5}$	$5.55\times 10^{5}$	$6.58\times 10^{5}$	$1.64\times 10^{11}$	$3.58\times 10^{5}$	$8.50\times 10^{5}$	$4.49\times 10^{5}$
	Std	$6.61\times 10^{4}$	$4.64\times 10^{4}$	$1.06\times 10^{5}$	$1.07\times 10^{5}$	$9.82\times 10^{4}$	$8.91\times 10^{11}$	$3.36\times 10^{4}$	$1.18\times 10^{5}$	$1.49\times 10^{5}$
	*p*-value	–	0.001	<$10^{-11}$	<$10^{-11}$	<$10^{-11}$	<$10^{-11}$	0.0002	<$10^{-11}$	0.5857
	Rank	5	3	11	10	12	18	2	15	7
F4	Mean	$4.43\times 10^{3}$	$3.08\times 10^{3}$	$2.61\times 10^{4}$	$2.50\times 10^{4}$	$1.34\times 10^{4}$	$3.43\times 10^{5}$	$4.70\times 10^{3}$	$1.05\times 10^{4}$	$1.19\times 10^{3}$
	Std	$1.07\times 10^{3}$	$7.40\times 10^{2}$	$6.86\times 10^{3}$	$4.97\times 10^{3}$	$3.99\times 10^{3}$	$4.45\times 10^{4}$	$8.58\times 10^{2}$	$3.66\times 10^{3}$	$7.84\times 10^{1}$
	*p*-value	–	0.0001	<$10^{-11}$	<$10^{-11}$	<$10^{-11}$	<$10^{-11}$	0.2134	<$10^{-11}$	<$10^{-11}$
	Rank	5	3	13	12	10	18	6	9	1
F5	Mean	$1.35\times 10^{3}$	$1.44\times 10^{3}$	$1.52\times 10^{3}$	$1.52\times 10^{3}$	$2.22\times 10^{3}$	$3.19\times 10^{3}$	$1.41\times 10^{3}$	$1.89\times 10^{3}$	$1.80\times 10^{3}$
	Std	$7.66\times 10^{1}$	$1.20\times 10^{2}$	$7.10\times 10^{1}$	$8.51\times 10^{1}$	$1.62\times 10^{2}$	$1.01\times 10^{2}$	$8.81\times 10^{1}$	$2.03\times 10^{2}$	$1.03\times 10^{2}$
	*p*-value	–	0.0053	<$10^{-11}$	<$10^{-11}$	<$10^{-11}$	<$10^{-11}$	0.0157	<$10^{-11}$	<$10^{-11}$
	Rank	1	3	5	5	14	18	2	12	10
F6	Mean	$6.55\times 10^{2}$	$6.59\times 10^{2}$	$6.52\times 10^{2}$	$6.54\times 10^{2}$	$7.06\times 10^{2}$	$7.61\times 10^{2}$	$6.64\times 10^{2}$	$6.85\times 10^{2}$	$6.83\times 10^{2}$
	Std	$5.89\times 10^{0}$	$8.70\times 10^{0}$	$6.82\times 10^{0}$	$7.82\times 10^{0}$	$8.24\times 10^{0}$	$5.71\times 10^{0}$	$5.74\times 10^{0}$	$8.72\times 10^{0}$	$3.80\times 10^{0}$
	*p*-value	–	0.0428	0.0571	0.6884	<$10^{-11}$	<$10^{-11}$	<$10^{-11}$	<$10^{-11}$	<$10^{-11}$
	Rank	3	5	1	2	13	18	8	15	14
F7	Mean	$2.80\times 10^{3}$	$2.60\times 10^{3}$	$2.99\times 10^{3}$	$2.82\times 10^{3}$	$4.72\times 10^{3}$	$1.36\times 10^{4}$	$2.67\times 10^{3}$	$6.45\times 10^{3}$	$4.19\times 10^{3}$
	Std	$2.50\times 10^{2}$	$1.99\times 10^{2}$	$2.62\times 10^{2}$	$1.87\times 10^{2}$	$7.36\times 10^{2}$	$7.09\times 10^{2}$	$2.37\times 10^{2}$	$5.56\times 10^{2}$	$1.62\times 10^{2}$
	*p*-value	–	0.001	0.027	0.959	<$10^{-11}$	<$10^{-11}$	0.0407	<$10^{-11}$	<$10^{-11}$
	Rank	3	2	6	4	13	18	5	16	12
F8	Mean	$1.65\times 10^{3}$	$1.71\times 10^{3}$	$1.79\times 10^{3}$	$1.82\times 10^{3}$	$2.50\times 10^{3}$	$3.49\times 10^{3}$	$1.70\times 10^{3}$	$2.20\times 10^{3}$	$2.07\times 10^{3}$
	Std	$9.21\times 10^{1}$	$9.16\times 10^{1}$	$7.56\times 10^{1}$	$6.81\times 10^{1}$	$1.76\times 10^{2}$	$1.19\times 10^{2}$	$8.85\times 10^{1}$	$1.57\times 10^{2}$	$8.06\times 10^{1}$
	*p*-value	–	0.0687	<$10^{-11}$	<$10^{-11}$	<$10^{-11}$	<$10^{-11}$	0.0495	<$10^{-11}$	<$10^{-11}$
	Rank	1	3	5	6	13	18	2	12	10
F9	Mean	$3.25\times 10^{4}$	$4.46\times 10^{4}$	$4.54\times 10^{4}$	$4.55\times 10^{4}$	$9.39\times 10^{4}$	$1.01\times 10^{5}$	$4.18\times 10^{4}$	$7.39\times 10^{4}$	$5.31\times 10^{4}$
	Std	$6.23\times 10^{3}$	$1.37\times 10^{4}$	$8.58\times 10^{3}$	$8.31\times 10^{3}$	$1.76\times 10^{4}$	$4.11\times 10^{3}$	$7.85\times 10^{3}$	$1.15\times 10^{4}$	$5.14\times 10^{3}$
	*p*-value	–	0.001	<$10^{-11}$	<$10^{-11}$	<$10^{-11}$	<$10^{-11}$	0.0001	<$10^{-11}$	<$10^{-11}$
	Rank	2	6	7	8	14	16	5	13	10
F10	Mean	$2.05\times 10^{4}$	$2.76\times 10^{4}$	$2.68\times 10^{4}$	$2.73\times 10^{4}$	$2.86\times 10^{4}$	$3.47\times 10^{4}$	$2.67\times 10^{4}$	$1.87\times 10^{4}$	$2.64\times 10^{4}$
	Std	$1.49\times 10^{3}$	$2.04\times 10^{3}$	$1.74\times 10^{3}$	$1.74\times 10^{3}$	$1.55\times 10^{3}$	$5.49\times 10^{2}$	$1.86\times 10^{3}$	$1.84\times 10^{3}$	$1.88\times 10^{3}$
	*p*-value	–	<$10^{-11}$	<$10^{-11}$	<$10^{-11}$	<$10^{-11}$	<$10^{-11}$	<$10^{-11}$	0.0002	<$10^{-11}$
	Rank	2	8	6	7	10	17	5	1	4
F11	Mean	$7.87\times 10^{4}$	$7.59\times 10^{4}$	$1.82\times 10^{5}$	$1.75\times 10^{5}$	$1.66\times 10^{5}$	$5.28\times 10^{9}$	$7.33\times 10^{4}$	$1.06\times 10^{5}$	$4.79\times 10^{4}$
	Std	$2.05\times 10^{4}$	$1.96\times 10^{4}$	$4.12\times 10^{4}$	$3.30\times 10^{4}$	$4.47\times 10^{4}$	$1.93\times 10^{10}$	$1.64\times 10^{4}$	$4.71\times 10^{4}$	$1.02\times 10^{4}$
	*p*-value	–	0.544	<$10^{-11}$	<$10^{-11}$	<$10^{-11}$	<$10^{-11}$	0.3286	0.0166	<$10^{-11}$
	Rank	5	4	13	12	11	18	3	8	2
F12	Mean	$2.10\times 10^{9}$	$1.37\times 10^{9}$	$3.30\times 10^{10}$	$3.00\times 10^{10}$	$1.24\times 10^{10}$	$3.89\times 10^{11}$	$4.05\times 10^{9}$	$5.88\times 10^{9}$	$6.18\times 10^{8}$
	Std	$9.07\times 10^{8}$	$5.05\times 10^{8}$	$8.84\times 10^{9}$	$8.55\times 10^{9}$	$2.68\times 10^{9}$	$4.65\times 10^{10}$	$1.13\times 10^{9}$	$4.87\times 10^{9}$	$1.99\times 10^{8}$
	*p*-value	–	0.0004	<$10^{-11}$	<$10^{-11}$	<$10^{-11}$	<$10^{-11}$	<$10^{-11}$	0.0003	<$10^{-11}$
	Rank	5	4	13	12	10	18	7	8	2
F13	Mean	$6.61\times 10^{6}$	$3.38\times 10^{6}$	$3.74\times 10^{9}$	$3.92\times 10^{9}$	$6.95\times 10^{8}$	$9.75\times 10^{10}$	$1.50\times 10^{8}$	$1.61\times 10^{7}$	$2.28\times 10^{7}$
	Std	$5.66\times 10^{6}$	$7.26\times 10^{6}$	$2.78\times 10^{9}$	$1.79\times 10^{9}$	$2.42\times 10^{8}$	$1.15\times 10^{10}$	$7.87\times 10^{7}$	$4.85\times 10^{7}$	$1.44\times 10^{7}$
	*p*-value	–	0.0003	<$10^{-11}$	<$10^{-11}$	<$10^{-11}$	<$10^{-11}$	<$10^{-11}$	0.0028	<$10^{-11}$
	Rank	5	4	12	13	8	18	6	7	3
F14	Mean	$3.20\times 10^{6}$	$2.81\times 10^{6}$	$8.05\times 10^{6}$	$6.98\times 10^{6}$	$3.57\times 10^{7}$	$1.10\times 10^{9}$	$2.81\times 10^{6}$	$7.66\times 10^{6}$	$3.72\times 10^{6}$
	Std	$1.64\times 10^{6}$	$2.08\times 10^{6}$	$4.68\times 10^{6}$	$3.25\times 10^{6}$	$2.02\times 10^{7}$	$4.79\times 10^{8}$	$1.42\times 10^{6}$	$5.73\times 10^{6}$	$9.72\times 10^{5}$
	*p*-value	–	0.1529	<$10^{-11}$	0.0001	<$10^{-11}$	<$10^{-11}$	0.2536	<$10^{-11}$	0.0545
	Rank	4	2	11	9	14	18	2	10	6
F15	Mean	$1.62\times 10^{5}$	$1.41\times 10^{6}$	$5.55\times 10^{8}$	$3.87\times 10^{8}$	$1.50\times 10^{8}$	$5.29\times 10^{10}$	$1.40\times 10^{7}$	$1.02\times 10^{6}$	$8.67\times 10^{6}$
	Std	$1.85\times 10^{5}$	$3.72\times 10^{6}$	$6.11\times 10^{8}$	$2.69\times 10^{8}$	$5.09\times 10^{7}$	$7.88\times 10^{9}$	$1.30\times 10^{7}$	$3.61\times 10^{6}$	$5.97\times 10^{6}$
	*p*-value	–	0.7189	<$10^{-11}$	<$10^{-11}$	<$10^{-11}$	<$10^{-11}$	<$10^{-11}$	0.3933	<$10^{-11}$
	Rank	1	5	13	11	7	18	4	3	6
F16	Mean	$6.94\times 10^{3}$	$7.94\times 10^{3}$	$8.58\times 10^{3}$	$8.83\times 10^{3}$	$1.28\times 10^{4}$	$4.46\times 10^{4}$	$8.59\times 10^{3}$	$8.68\times 10^{3}$	$8.60\times 10^{3}$
	Std	$7.34\times 10^{2}$	$1.06\times 10^{3}$	$6.26\times 10^{2}$	$7.25\times 10^{2}$	$1.63\times 10^{3}$	$7.67\times 10^{3}$	$8.99\times 10^{2}$	$1.29\times 10^{3}$	$9.08\times 10^{2}$
	*p*-value	–	0.001	<$10^{-11}$	<$10^{-11}$	<$10^{-11}$	<$10^{-11}$	<$10^{-11}$	<$10^{-11}$	<$10^{-11}$
	Rank	1	4	6	8	14	18	7	9	5
F17	Mean	$5.59\times 10^{3}$	$6.29\times 10^{3}$	$6.40\times 10^{3}$	$6.51\times 10^{3}$	$8.76\times 10^{3}$	$1.56\times 10^{8}$	$6.33\times 10^{3}$	$7.17\times 10^{3}$	$7.12\times 10^{3}$
	Std	$5.13\times 10^{2}$	$7.46\times 10^{2}$	$8.53\times 10^{2}$	$1.10\times 10^{3}$	$7.56\times 10^{2}$	$9.76\times 10^{7}$	$6.16\times 10^{2}$	$6.91\times 10^{2}$	$6.83\times 10^{2}$
	*p*-value	–	0.0003	<$10^{-11}$	0.0001	<$10^{-11}$	<$10^{-11}$	0.0001	<$10^{-11}$	<$10^{-11}$
	Rank	1	3	5	6	12	18	4	9	8
F18	Mean	$3.54\times 10^{6}$	$4.64\times 10^{6}$	$7.22\times 10^{6}$	$9.91\times 10^{6}$	$3.73\times 10^{7}$	$1.90\times 10^{9}$	$3.78\times 10^{6}$	$1.16\times 10^{7}$	$6.28\times 10^{6}$
	Std	$2.17\times 10^{6}$	$3.19\times 10^{6}$	$4.18\times 10^{6}$	$9.11\times 10^{6}$	$2.40\times 10^{7}$	$6.22\times 10^{8}$	$1.72\times 10^{6}$	$6.92\times 10^{6}$	$2.25\times 10^{6}$
	*p*-value	–	0.1846	0.0004	<$10^{-11}$	<$10^{-11}$	<$10^{-11}$	0.517	<$10^{-11}$	0.0002
	Rank	1	3	5	7	13	18	2	9	4
F19	Mean	$4.51\times 10^{5}$	$6.84\times 10^{5}$	$5.56\times 10^{8}$	$5.65\times 10^{8}$	$2.11\times 10^{8}$	$5.23\times 10^{10}$	$1.62\times 10^{7}$	$4.19\times 10^{6}$	$1.19\times 10^{7}$
	Std	$5.65\times 10^{5}$	$2.10\times 10^{6}$	$6.00\times 10^{8}$	$4.69\times 10^{8}$	$8.33\times 10^{7}$	$9.86\times 10^{9}$	$8.10\times 10^{6}$	$4.04\times 10^{6}$	$1.56\times 10^{7}$
	*p*-value	–	0.165	<$10^{-11}$	<$10^{-11}$	<$10^{-11}$	<$10^{-11}$	<$10^{-11}$	<$10^{-11}$	<$10^{-11}$
	Rank	1	3	13	14	10	18	6	5	7
F20	Mean	$5.47\times 10^{3}$	$6.65\times 10^{3}$	$6.33\times 10^{3}$	$6.49\times 10^{3}$	$6.68\times 10^{3}$	$8.60\times 10^{3}$	$6.16\times 10^{3}$	$6.24\times 10^{3}$	$6.57\times 10^{3}$
	Std	$4.52\times 10^{2}$	$5.49\times 10^{2}$	$4.18\times 10^{2}$	$6.43\times 10^{2}$	$7.66\times 10^{2}$	$2.94\times 10^{2}$	$4.80\times 10^{2}$	$6.42\times 10^{2}$	$5.83\times 10^{2}$
	*p*-value	–	<$10^{-11}$	<$10^{-11}$	<$10^{-11}$	<$10^{-11}$	<$10^{-11}$	0.0002	<$10^{-11}$	<$10^{-11}$
	Rank	1	8	5	7	9	18	4	6	3
F21	Mean	$3.15\times 10^{3}$	$3.28\times 10^{3}$	$3.37\times 10^{3}$	$3.34\times 10^{3}$	$4.22\times 10^{3}$	$5.56\times 10^{3}$	$3.26\times 10^{3}$	$3.99\times 10^{3}$	$4.70\times 10^{3}$
	Std	$8.86\times 10^{1}$	$1.18\times 10^{2}$	$8.37\times 10^{1}$	$7.45\times 10^{1}$	$1.73\times 10^{2}$	$2.20\times 10^{2}$	$8.49\times 10^{1}$	$2.01\times 10^{2}$	$2.20\times 10^{2}$
	*p*-value	–	0.0013	<$10^{-11}$	<$10^{-11}$	<$10^{-11}$	<$10^{-11}$	0.0001	<$10^{-11}$	<$10^{-11}$
	Rank	1	3	5	4	10	18	2	9	15
F22	Mean	$2.36\times 10^{4}$	$2.92\times 10^{4}$	$2.94\times 10^{4}$	$3.01\times 10^{4}$	$3.00\times 10^{4}$	$3.68\times 10^{4}$	$2.88\times 10^{4}$	$2.04\times 10^{4}$	$2.77\times 10^{4}$
	Std	$1.64\times 10^{3}$	$1.92\times 10^{3}$	$1.89\times 10^{3}$	$2.27\times 10^{3}$	$2.04\times 10^{3}$	$5.77\times 10^{2}$	$1.81\times 10^{3}$	$1.24\times 10^{3}$	$1.94\times 10^{3}$
	*p*-value	–	<$10^{-11}$	<$10^{-11}$	<$10^{-11}$	<$10^{-11}$	<$10^{-11}$	<$10^{-11}$	<$10^{-11}$	<$10^{-11}$
	Rank	2	8	9	11	10	18	7	1	6
F23	Mean	$3.65\times 10^{3}$	$3.79\times 10^{3}$	$3.94\times 10^{3}$	$3.92\times 10^{3}$	$4.82\times 10^{3}$	$8.70\times 10^{3}$	$3.93\times 10^{3}$	$4.83\times 10^{3}$	$5.72\times 10^{3}$
	Std	$9.42\times 10^{1}$	$1.52\times 10^{2}$	$9.15\times 10^{1}$	$1.23\times 10^{2}$	$2.90\times 10^{302}$	$6.41\times 10^{602}$	$1.04\times 10^{102}$	$2.56\times 10^{202}$	$5.60\times 10^{202}$
	*p*-value	–	0.0002	<$10^{-11}$	<$10^{-11}$	<$10^{-11}$	<$10^{-11}$	<$10^{-11}$	<$10^{-11}$	<$10^{-11}$
	Rank	1	2	5	4	9	18	6	10	15
F24	Mean	$4.40\times 10^{3}$	$4.51\times 10^{3}$	$4.96\times 10^{3}$	$4.95\times 10^{3}$	$5.81\times 10^{3}$	$1.50\times 10^{4}$	$4.53\times 10^{3}$	$6.13\times 10^{3}$	$7.80\times 10^{3}$
	Std	$2.15\times 10^{202}$	$2.19\times 10^{202}$	$3.02\times 10^{202}$	$2.63\times 10^{202}$	$5.13\times 10^{202}$	$1.37\times 10^{3}$	$1.03\times 10^{102}$	$4.09\times 10^{202}$	$5.91\times 10^{202}$
	*p*-value	–	0.1589	<$10^{-11}$	<$10^{-11}$	<$10^{-11}$	<$10^{-11}$	0.0117	<$10^{-11}$	<$10^{-11}$
	Rank	1	2	6	5	9	18	3	11	16
F25	Mean	$5.97\times 10^{3}$	$5.84\times 10^{3}$	$1.24\times 10^{4}$	$1.24\times 10^{4}$	$1.38\times 10^{4}$	$1.44\times 10^{5}$	$6.90\times 10^{3}$	$7.87\times 10^{3}$	$3.82\times 10^{3}$
	Std	$4.86\times 10^{202}$	$5.99\times 10^{202}$	$1.95\times 10^{3}$	$1.62\times 10^{3}$	$2.18\times 10^{3}$	$2.08\times 10^{4}$	$7.79\times 10^{702}$	$1.30\times 10^{3}$	$6.77\times 10^{1}$
	*p*-value	–	0.4908	<$10^{-11}$	<$10^{-11}$	<$10^{-11}$	<$10^{-11}$	<$10^{-11}$	<$10^{-11}$	<$10^{-11}$
	Rank	5	4	13	12	14	18	7	9	1
F26	Mean	$1.88\times 10^{4}$	$1.69\times 10^{4}$	$2.59\times 10^{4}$	$2.56\times 10^{4}$	$3.27\times 10^{4}$	$1.01\times 10^{5}$	$1.72\times 10^{4}$	$3.52\times 10^{4}$	$3.07\times 10^{4}$
	Std	$2.43\times 10^{3}$	$1.71\times 10^{3}$	$2.44\times 10^{3}$	$2.04\times 10^{3}$	$6.85\times 10^{3}$	$1.15\times 10^{4}$	$9.92\times 10^{2}$	$3.22\times 10^{3}$	$1.90\times 10^{3}$
	*p*-value	–	0.0032	<$10^{-11}$	<$10^{-11}$	<$10^{-11}$	<$10^{-11}$	0.0018	<$10^{-11}$	<$10^{-11}$
	Rank	4	2	8	7	12	18	3	14	11
F27	Mean	$3.98\times 10^{3}$	$3.78\times 10^{3}$	$5.11\times 10^{3}$	$5.08\times 10^{3}$	$5.19\times 10^{3}$	$1.85\times 10^{4}$	$4.41\times 10^{3}$	$5.12\times 10^{3}$	$5.00\times 10^{3}$
	Std	$1.72\times 10^{2}$	$1.27\times 10^{2}$	$3.82\times 10^{302}$	$3.78\times 10^{302}$	$5.15\times 10^{202}$	$1.86\times 10^{3}$	$2.92\times 10^{202}$	$5.61\times 10^{202}$	$5.33\times 10^{202}$
	*p*-value	–	<$10^{-11}$	<$10^{-11}$	<$10^{-11}$	<$10^{-11}$	<$10^{-11}$	<$10^{-11}$	<$10^{-11}$	<$10^{-11}$
	Rank	3	2	10	9	11	18	5	12	8
F28	Mean	$8.12\times 10^{3}$	$9.78\times 10^{3}$	$1.79\times 10^{4}$	$1.71\times 10^{4}$	$1.72\times 10^{4}$	$7.63\times 10^{4}$	$1.12\times 10^{4}$	$1.54\times 10^{4}$	$3.82\times 10^{3}$
	Std	$1.14\times 10^{3}$	$3.22\times 10^{3}$	$1.96\times 10^{3}$	$1.56\times 10^{3}$	$2.39\times 10^{3}$	$7.61\times 10^{3}$	$1.36\times 10^{3}$	$4.79\times 10^{3}$	$6.11\times 10^{1}$
	*p*-value	–	0.001	<$10^{-11}$	<$10^{-11}$	<$10^{-11}$	<$10^{-11}$	<$10^{-11}$	<$10^{-11}$	<$10^{-11}$
	Rank	6	8	14	13	12	18	9	11	1
F29	Mean	$8.94\times 10^{3}$	$8.66\times 10^{3}$	$1.20\times 10^{4}$	$1.22\times 10^{4}$	$1.62\times 10^{4}$	$2.78\times 10^{7}$	$1.07\times 10^{4}$	$1.18\times 10^{4}$	$1.11\times 10^{4}$
	Std	$1.02\times 10^{3}$	$7.88\times 10^{2}$	$1.43\times 10^{3}$	$1.29\times 10^{3}$	$2.11\times 10^{3}$	$1.94\times 10^{7}$	$7.60\times 10^{702}$	$1.55\times 10^{3}$	$1.47\times 10^{3}$
	*p*-value	–	0.2059	<$10^{-11}$	<$10^{-11}$	<$10^{-11}$	<$10^{-11}$	<$10^{-11}$	<$10^{-11}$	<$10^{-11}$
	Rank	3	2	9	10	14	18	5	8	6
F30	Mean	$1.05\times 10^{7}$	$5.12\times 10^{6}$	$2.51\times 10^{9}$	$2.97\times 10^{9}$	$1.14\times 10^{9}$	$8.27\times 10^{10}$	$1.55\times 10^{8}$	$9.06\times 10^{7}$	$5.31\times 10^{7}$
	Std	$8.28\times 10^{6}$	$2.90\times 10^{6}$	$1.85\times 10^{9}$	$1.61\times 10^{9}$	$4.27\times 10^{8}$	$1.52\times 10^{10}$	$5.76\times 10^{7}$	$7.35\times 10^{7}$	$1.93\times 10^{7}$
	*p*-value	–	0.0036	<$10^{-11}$	<$10^{-11}$	<$10^{-11}$	<$10^{-11}$	<$10^{-11}$	<$10^{-11}$	<$10^{-11}$
	Rank	4	3	13	14	11	18	7	6	5
+/≈/−		–	12/9/8	28/1/0	27/2/0	29/0/0	29/0/0	22/5/2	27/1/1	20/3/6
Mean rank		2.55	3.69	7.62	7.28	10.76	17.69	4.72	7.93	6.28
Final ranking		1	2	9	8	13	18	4	10	6

**Table A8:** Comparison of HALA and other algorithms on the CEC2017 benchmark suite (Dim = 100) (excluding F2)—Part 2: HALA vs. CQALA, DFL, DMOA, DHOA, FGO, KLA, PGA, SO, SOO (continued from Table A7).

Function	Metric	CQALA	DFL	DMOA	DHOA	FGO	KLA	PGA	SO	SOO
F1	Mean	$2.27\times 10^{10}$	$1.78\times 10^{10}$	$2.28\times 10^{10}$	$4.17\times 10^{10}$	$1.04\times 10^{11}$	$6.42\times 10^{11}$	$2.00\times 10^{10}$	$3.58\times 10^{11}$	$8.72\times 10^{10}$
	Std	$9.34\times 10^{9}$	$6.01\times 10^{9}$	$1.39\times 10^{9}$	$9.25\times 10^{9}$	$1.30\times 10^{10}$	$5.33\times 10^{10}$	$4.59\times 10^{9}$	$3.47\times 10^{10}$	$1.84\times 10^{10}$
	*p*-value	0.2134	0.0003	0.0472	<$10^{-11}$	<$10^{-11}$	<$10^{-11}$	0.0028	<$10^{-11}$	<$10^{-11}$
	Rank	8	2	11	14	15	17	6	16	10
F3	Mean	$4.81\times 10^{5}$	$1.12\times 10^{6}$	$5.33\times 10^{5}$	$4.41\times 10^{5}$	$7.39\times 10^{5}$	$1.92\times 10^{13}$	$6.98\times 10^{5}$	$1.15\times 10^{6}$	$1.11\times 10^{6}$
	Std	$2.16\times 10^{5}$	$1.85\times 10^{5}$	$4.52\times 10^{4}$	$2.96\times 10^{4}$	$1.01\times 10^{5}$	$5.10\times 10^{13}$	$1.01\times 10^{5}$	$9.84\times 10^{4}$	$2.68\times 10^{5}$
	*p*-value	0.1306	<$10^{-11}$	<$10^{-11}$	0.2536	<$10^{-11}$	<$10^{-11}$	<$10^{-11}$	<$10^{-11}$	<$10^{-11}$
	Rank	8	16	9	6	13	17	14	3	1
F4	Mean	$3.67\times 10^{3}$	$3.92\times 10^{3}$	$3.20\times 10^{3}$	$5.20\times 10^{3}$	$1.35\times 10^{4}$	$3.47\times 10^{5}$	$4.57\times 10^{3}$	$8.69\times 10^{4}$	$1.43\times 10^{4}$
	Std	$1.26\times 10^{3}$	$9.38\times 10^{902}$	$2.36\times 10^{202}$	$1.22\times 10^{3}$	$2.91\times 10^{3}$	$6.00\times 10^{4}$	$1.38\times 10^{3}$	$1.92\times 10^{4}$	$3.60\times 10^{3}$
	*p*-value	0.0387	0.0897	<$10^{-11}$	0.0407	<$10^{-11}$	<$10^{-11}$	0.9263	<$10^{-11}$	<$10^{-11}$
	Rank	4	7	2	8	11	17	14	16	15
F5	Mean	$1.36\times 10^{3}$	$2.02\times 10^{3}$	$1.65\times 10^{3}$	$1.51\times 10^{3}$	$1.91\times 10^{3}$	$2.41\times 10^{3}$	$1.78\times 10^{3}$	$2.68\times 10^{3}$	$1.61\times 10^{3}$
	Std	$4.21\times 10^{41}$	$1.25\times 10^{102}$	$3.17\times 10^{31}$	$6.40\times 10^{61}$	$1.06\times 10^{102}$	$1.99\times 10^{102}$	$1.66\times 10^{102}$	$1.41\times 10^{102}$	$8.25\times 10^{81}$
	*p*-value	0.36	<$10^{-11}$	<$10^{-11}$	<$10^{-11}$	<$10^{-11}$	<$10^{-11}$	<$10^{-11}$	<$10^{-11}$	<$10^{-11}$
	Rank	2	16	8	6	13	17	11	18	9
F6	Mean	$6.55\times 10^{2}$	$6.96\times 10^{2}$	$6.46\times 10^{2}$	$6.65\times 10^{2}$	$6.92\times 10^{2}$	$7.00\times 10^{2}$	$6.89\times 10^{2}$	$7.41\times 10^{2}$	$6.70\times 10^{2}$
	Std	$2.87\times 10^{0}$	$1.78\times 10^{1}$	$4.99\times 10^{0}$	$4.33\times 10^{0}$	$9.52\times 10^{0}$	$4.56\times 10^{0}$	$7.12\times 10^{0}$	$8.51\times 10^{0}$	$5.12\times 10^{0}$
	*p*-value	0.959	<$10^{-11}$	0.0001	<$10^{-11}$	<$10^{-11}$	<$10^{-11}$	<$10^{-11}$	<$10^{-11}$	<$10^{-11}$
	Rank	4	15	6	9	14	16	13	17	11
F7	Mean	$3.46\times 10^{3}$	$6.33\times 10^{3}$	$2.28\times 10^{3}$	$4.03\times 10^{3}$	$4.70\times 10^{3}$	$1.01\times 10^{4}$	$4.20\times 10^{3}$	$1.07\times 10^{4}$	$3.72\times 10^{3}$
	Std	$1.66\times 10^{202}$	$8.52\times 10^{802}$	$4.07\times 10^{41}$	$3.17\times 10^{302}$	$2.90\times 10^{302}$	$2.12\times 10^{3}$	$1.19\times 10^{3}$	$7.55\times 10^{702}$	$3.37\times 10^{302}$
	*p*-value	<$10^{-11}$	<$10^{-11}$	<$10^{-11}$	<$10^{-11}$	<$10^{-11}$	<$10^{-11}$	<$10^{-11}$	<$10^{-11}$	<$10^{-11}$
	Rank	7	17	1	9	14	15	11	16	10
F8	Mean	$1.67\times 10^{3}$	$2.33\times 10^{3}$	$1.92\times 10^{3}$	$1.82\times 10^{3}$	$2.21\times 10^{3}$	$2.72\times 10^{3}$	$2.09\times 10^{3}$	$2.87\times 10^{3}$	$1.90\times 10^{3}$
	Std	$5.06\times 10^{51}$	$1.37\times 10^{102}$	$3.85\times 10^{41}$	$4.96\times 10^{41}$	$9.50\times 10^{91}$	$1.71\times 10^{102}$	$1.24\times 10^{102}$	$1.35\times 10^{102}$	$7.96\times 10^{81}$
	*p*-value	0.544	<$10^{-11}$	<$10^{-11}$	<$10^{-11}$	<$10^{-11}$	<$10^{-11}$	<$10^{-11}$	<$10^{-11}$	<$10^{-11}$
	Rank	4	14	7	5	11	17	9	16	8
F9	Mean	$2.52\times 10^{4}$	$1.27\times 10^{5}$	$3.21\times 10^{4}$	$4.42\times 10^{4}$	$9.02\times 10^{4}$	$1.90\times 10^{5}$	$8.55\times 10^{4}$	$1.80\times 10^{5}$	$3.92\times 10^{4}$
	Std	$1.51\times 10^{3}$	$2.34\times 10^{4}$	$8.44\times 10^{3}$	$6.19\times 10^{3}$	$1.13\times 10^{4}$	$3.67\times 10^{4}$	$1.75\times 10^{4}$	$2.18\times 10^{4}$	$5.34\times 10^{3}$
	*p*-value	<$10^{-11}$	<$10^{-11}$	0.5999	<$10^{-11}$	<$10^{-11}$	<$10^{-11}$	<$10^{-11}$	<$10^{-11}$	0.0002
	Rank	1	15	4	8	12	17	11	16	9
F10	Mean	$1.73\times 10^{4}$	$2.94\times 10^{4}$	$3.28\times 10^{4}$	$2.36\times 10^{4}$	$3.28\times 10^{4}$	$3.53\times 10^{4}$	$2.29\times 10^{4}$	$3.30\times 10^{4}$	$2.42\times 10^{4}$
	Std	$2.31\times 10^{3}$	$1.14\times 10^{3}$	$4.37\times 10^{2}$	$1.82\times 10^{3}$	$1.43\times 10^{3}$	$1.03\times 10^{3}$	$2.16\times 10^{3}$	$7.51\times 10^{702}$	$1.67\times 10^{3}$
	*p*-value	0.0001	<$10^{-11}$	<$10^{-11}$	<$10^{-11}$	<$10^{-11}$	<$10^{-11}$	<$10^{-11}$	<$10^{-11}$	<$10^{-11}$
	Rank	3	12	15	9	14	16	8	13	11
F11	Mean	$8.97\times 10^{4}$	$1.92\times 10^{5}$	$1.68\times 10^{5}$	$9.01\times 10^{4}$	$1.77\times 10^{5}$	$8.67\times 10^{9}$	$1.42\times 10^{5}$	$5.68\times 10^{5}$	$2.39\times 10^{5}$
	Std	$1.63\times 10^{4}$	$5.16\times 10^{4}$	$2.16\times 10^{4}$	$1.58\times 10^{4}$	$3.45\times 10^{4}$	$2.68\times 10^{10}$	$3.62\times 10^{4}$	$1.30\times 10^{5}$	$7.50\times 10^{4}$
	*p*-value	0.0125	<$10^{-11}$	<$10^{-11}$	0.0077	<$10^{-11}$	<$10^{-11}$	<$10^{-11}$	<$10^{-11}$	<$10^{-11}$
	Rank	6	12	10	7	9	17	11	15	14
F12	Mean	$3.03\times 10^{9}$	$3.54\times 10^{9}$	$8.23\times 10^{9}$	$4.03\times 10^{9}$	$2.55\times 10^{10}$	$3.84\times 10^{11}$	$1.79\times 10^{9}$	$8.20\times 10^{10}$	$1.55\times 10^{10}$
	Std	$3.99\times 10^{9}$	$1.12\times 10^{9}$	$8.98\times 10^{8}$	$1.27\times 10^{9}$	$6.12\times 10^{9}$	$4.57\times 10^{10}$	$6.91\times 10^{8}$	$1.77\times 10^{10}$	$4.75\times 10^{9}$
	*p*-value	0.7189	0.0001	<$10^{-11}$	<$10^{-11}$	<$10^{-11}$	<$10^{-11}$	0.2989	<$10^{-11}$	<$10^{-11}$
	Rank	6	9	14	11	15	17	3	16	10
F13	Mean	$1.28\times 10^{8}$	$9.61\times 10^{7}$	$1.47\times 10^{9}$	$2.11\times 10^{8}$	$4.24\times 10^{9}$	$9.71\times 10^{10}$	$2.32\times 10^{6}$	$1.56\times 10^{10}$	$8.23\times 10^{8}$
	Std	$6.80\times 10^{8}$	$9.87\times 10^{7}$	$1.78\times 10^{8}$	$1.50\times 10^{8}$	$1.46\times 10^{9}$	$1.46\times 10^{10}$	$4.94\times 10^{5}$	$3.67\times 10^{9}$	$6.03\times 10^{8}$
	*p*-value	0.0387	<$10^{-11}$	<$10^{-11}$	<$10^{-11}$	<$10^{-11}$	<$10^{-11}$	0.0001	<$10^{-11}$	<$10^{-11}$
	Rank	9	8	14	10	15	16	1	17	12
F14	Mean	$4.46\times 10^{6}$	$7.52\times 10^{6}$	$1.72\times 10^{7}$	$9.10\times 10^{6}$	$3.89\times 10^{7}$	$1.17\times 10^{9}$	$9.81\times 10^{6}$	$5.66\times 10^{7}$	$1.68\times 10^{7}$
	Std	$1.58\times 10^{6}$	$7.28\times 10^{6}$	$4.23\times 10^{6}$	$3.07\times 10^{6}$	$1.72\times 10^{7}$	$6.27\times 10^{8}$	$5.60\times 10^{6}$	$2.78\times 10^{7}$	$1.18\times 10^{7}$
	*p*-value	0.0013	0.0012	<$10^{-11}$	<$10^{-11}$	<$10^{-11}$	<$10^{-11}$	<$10^{-11}$	<$10^{-11}$	<$10^{-11}$
	Rank	7	8	13	12	15	17	16	3	14
F15	Mean	$9.94\times 10^{5}$	$4.16\times 10^{7}$	$5.92\times 10^{8}$	$7.33\times 10^{7}$	$1.55\times 10^{9}$	$5.33\times 10^{10}$	$5.80\times 10^{5}$	$4.15\times 10^{9}$	$4.78\times 10^{7}$
	Std	$3.36\times 10^{6}$	$4.78\times 10^{7}$	$1.00\times 10^{8}$	$3.94\times 10^{7}$	$6.94\times 10^{8}$	$1.07\times 10^{10}$	$1.31\times 10^{5}$	$1.08\times 10^{9}$	$6.17\times 10^{7}$
	*p*-value	0.8451	<$10^{-11}$	<$10^{-11}$	<$10^{-11}$	<$10^{-11}$	<$10^{-11}$	<$10^{-11}$	<$10^{-11}$	<$10^{-11}$
	Rank	5	10	15	9	16	17	2	18	8
F16	Mean	$7.38\times 10^{3}$	$9.39\times 10^{3}$	$1.08\times 10^{4}$	$7.76\times 10^{3}$	$1.25\times 10^{4}$	$3.85\times 10^{4}$	$1.03\times 10^{4}$	$1.55\times 10^{4}$	$8.44\times 10^{3}$
	Std	$1.05\times 10^{3}$	$1.02\times 10^{3}$	$4.66\times 10^{2}$	$8.20\times 10^{802}$	$9.14\times 10^{902}$	$7.84\times 10^{3}$	$1.06\times 10^{3}$	$1.41\times 10^{3}$	$9.09\times 10^{902}$
	*p*-value	0.1779	<$10^{-11}$	<$10^{-11}$	0.0016	<$10^{-11}$	<$10^{-11}$	<$10^{-11}$	<$10^{-11}$	<$10^{-11}$
	Rank	2	10	12	5	13	17	11	16	8
F17	Mean	$6.86\times 10^{3}$	$7.89\times 10^{3}$	$7.99\times 10^{3}$	$6.18\times 10^{3}$	$1.03\times 10^{4}$	$1.03\times 10^{8}$	$7.24\times 10^{3}$	$3.35\times 10^{4}$	$8.26\times 10^{3}$
	Std	$9.85\times 10^{902}$	$9.51\times 10^{902}$	$3.84\times 10^{302}$	$5.68\times 10^{502}$	$8.31\times 10^{802}$	$8.98\times 10^{7}$	$8.77\times 10^{802}$	$4.16\times 10^{4}$	$2.16\times 10^{3}$
	*p*-value	<$10^{-11}$	<$10^{-11}$	<$10^{-11}$	0.0006	<$10^{-11}$	<$10^{-11}$	<$10^{-11}$	<$10^{-11}$	<$10^{-11}$
	Rank	5	10	11	2	14	17	7	16	12
F18	Mean	$4.63\times 10^{6}$	$1.03\times 10^{7}$	$2.47\times 10^{7}$	$6.76\times 10^{6}$	$5.18\times 10^{7}$	$1.94\times 10^{9}$	$1.25\times 10^{7}$	$1.44\times 10^{8}$	$1.41\times 10^{7}$
	Std	$1.98\times 10^{6}$	$6.62\times 10^{6}$	$5.65\times 10^{6}$	$4.02\times 10^{6}$	$1.75\times 10^{7}$	$8.64\times 10^{8}$	$4.93\times 10^{6}$	$5.39\times 10^{7}$	$1.01\times 10^{7}$
	*p*-value	0.0175	<$10^{-11}$	<$10^{-11}$	0.0006	<$10^{-11}$	<$10^{-11}$	<$10^{-11}$	<$10^{-11}$	<$10^{-11}$
	Rank	6	8	12	7	15	17	10	18	11
F19	Mean	$5.67\times 10^{5}$	$9.21\times 10^{7}$	$5.96\times 10^{8}$	$5.89\times 10^{7}$	$1.32\times 10^{9}$	$5.77\times 10^{10}$	$4.37\times 10^{7}$	$4.80\times 10^{9}$	$1.27\times 10^{8}$
	Std	$9.98\times 10^{5}$	$5.91\times 10^{7}$	$9.75\times 10^{7}$	$3.73\times 10^{7}$	$6.63\times 10^{8}$	$1.03\times 10^{10}$	$3.39\times 10^{7}$	$1.71\times 10^{9}$	$1.92\times 10^{8}$
	*p*-value	0.9426	<$10^{-11}$	<$10^{-11}$	<$10^{-11}$	<$10^{-11}$	<$10^{-11}$	<$10^{-11}$	<$10^{-11}$	<$10^{-11}$
	Rank	4	11	15	9	16	17	8	18	12
F20	Mean	$5.44\times 10^{3}$	$7.09\times 10^{3}$	$7.54\times 10^{3}$	$5.53\times 10^{3}$	$8.00\times 10^{3}$	$9.10\times 10^{3}$	$5.53\times 10^{3}$	$8.25\times 10^{3}$	$6.36\times 10^{3}$
	Std	$4.95\times 10^{502}$	$5.76\times 10^{502}$	$3.33\times 10^{302}$	$5.86\times 10^{502}$	$2.39\times 10^{202}$	$3.73\times 10^{302}$	$3.28\times 10^{302}$	$4.18\times 10^{202}$	$5.20\times 10^{502}$
	*p*-value	0.7813	<$10^{-11}$	<$10^{-11}$	0.9426	<$10^{-11}$	<$10^{-11}$	0.4653	<$10^{-11}$	<$10^{-11}$
	Rank	2	10	12	5	14	17	3	15	11
F21	Mean	$3.59\times 10^{3}$	$4.04\times 10^{3}$	$3.41\times 10^{3}$	$3.24\times 10^{3}$	$3.82\times 10^{3}$	$5.20\times 10^{3}$	$3.88\times 10^{3}$	$4.43\times 10^{3}$	$3.60\times 10^{3}$
	Std	$2.11\times 10^{202}$	$1.59\times 10^{102}$	$3.19\times 10^{31}$	$7.18\times 10^{71}$	$1.45\times 10^{102}$	$2.88\times 10^{202}$	$1.77\times 10^{102}$	$1.44\times 10^{102}$	$1.60\times 10^{102}$
	*p*-value	<$10^{-11}$	<$10^{-11}$	<$10^{-11}$	0.0006	<$10^{-11}$	<$10^{-11}$	<$10^{-11}$	<$10^{-11}$	<$10^{-11}$
	Rank	6	11	4	2	8	17	9	14	7
F22	Mean	$2.13\times 10^{4}$	$3.13\times 10^{4}$	$3.46\times 10^{4}$	$2.45\times 10^{4}$	$3.51\times 10^{4}$	$3.76\times 10^{4}$	$2.50\times 10^{4}$	$3.45\times 10^{4}$	$2.69\times 10^{4}$
	Std	$1.50\times 10^{3}$	$1.08\times 10^{3}$	$5.47\times 10^{502}$	$5.42\times 10^{3}$	$1.03\times 10^{3}$	$8.88\times 10^{802}$	$1.76\times 10^{3}$	$8.18\times 10^{802}$	$1.67\times 10^{3}$
	*p*-value	<$10^{-11}$	<$10^{-11}$	<$10^{-11}$	0.0068	<$10^{-11}$	<$10^{-11}$	0.0087	<$10^{-11}$	<$10^{-11}$
	Rank	3	12	15	5	16	17	13	14	4
F23	Mean	$4.16\times 10^{3}$	$4.65\times 10^{3}$	$3.99\times 10^{3}$	$3.81\times 10^{3}$	$4.29\times 10^{3}$	$6.54\times 10^{3}$	$4.43\times 10^{3}$	$4.75\times 10^{3}$	$4.38\times 10^{3}$
	Std	$2.01\times 10^{202}$	$1.91\times 10^{102}$	$5.97\times 10^{61}$	$8.41\times 10^{81}$	$1.53\times 10^{102}$	$4.47\times 10^{402}$	$2.16\times 10^{202}$	$1.30\times 10^{102}$	$1.94\times 10^{202}$
	*p*-value	<$10^{-11}$	<$10^{-11}$	<$10^{-11}$	<$10^{-11}$	<$10^{-11}$	<$10^{-11}$	<$10^{-11}$	<$10^{-11}$	<$10^{-11}$
	Rank	7	11	3	2	8	17	12	13	14
F24	Mean	$5.50\times 10^{3}$	$6.23\times 10^{3}$	$4.36\times 10^{3}$	$4.49\times 10^{3}$	$4.78\times 10^{3}$	$1.17\times 10^{4}$	$5.49\times 10^{3}$	$5.88\times 10^{3}$	$5.58\times 10^{3}$
	Std	$4.70\times 10^{402}$	$4.58\times 10^{402}$	$3.71\times 10^{31}$	$1.35\times 10^{102}$	$1.68\times 10^{102}$	$1.26\times 10^{3}$	$2.94\times 10^{202}$	$2.09\times 10^{202}$	$3.24\times 10^{202}$
	*p*-value	<$10^{-11}$	<$10^{-11}$	0.5577	0.0519	<$10^{-11}$	<$10^{-11}$	<$10^{-11}$	<$10^{-11}$	<$10^{-11}$
	Rank	10	14	4	7	8	17	9	12	13
F25	Mean	$5.07\times 10^{3}$	$6.34\times 10^{3}$	$5.77\times 10^{3}$	$7.66\times 10^{3}$	$1.64\times 10^{4}$	$1.50\times 10^{5}$	$5.81\times 10^{3}$	$6.84\times 10^{4}$	$9.90\times 10^{3}$
	Std	$5.90\times 10^{502}$	$8.37\times 10^{702}$	$2.52\times 10^{202}$	$8.96\times 10^{802}$	$2.39\times 10^{3}$	$2.48\times 10^{4}$	$5.81\times 10^{502}$	$9.44\times 10^{3}$	$1.50\times 10^{3}$
	*p*-value	<$10^{-11}$	0.0859	0.0627	<$10^{-11}$	<$10^{-11}$	<$10^{-11}$	0.4908	<$10^{-11}$	<$10^{-11}$
	Rank	6	10	8	11	15	17	9	16	12
F26	Mean	$2.65\times 10^{4}$	$3.49\times 10^{4}$	$1.80\times 10^{4}$	$2.87\times 10^{4}$	$2.12\times 10^{4}$	$9.36\times 10^{4}$	$3.07\times 10^{4}$	$2.99\times 10^{4}$	$2.87\times 10^{4}$
	Std	$2.32\times 10^{3}$	$3.86\times 10^{3}$	$2.53\times 10^{3}$	$1.86\times 10^{3}$	$1.39\times 10^{3}$	$1.40\times 10^{4}$	$4.88\times 10^{3}$	$2.16\times 10^{3}$	$2.57\times 10^{3}$
	*p*-value	<$10^{-11}$	<$10^{-11}$	0.3086	<$10^{-11}$	0.0001	<$10^{-11}$	<$10^{-11}$	<$10^{-11}$	<$10^{-11}$
	Rank	5	13	1	9	3	17	10	12	8
F27	Mean	$4.27\times 10^{3}$	$5.06\times 10^{3}$	$4.35\times 10^{3}$	$4.58\times 10^{3}$	$4.74\times 10^{3}$	$1.65\times 10^{4}$	$5.66\times 10^{3}$	$6.24\times 10^{3}$	$4.96\times 10^{3}$
	Std	$2.74\times 10^{202}$	$4.87\times 10^{402}$	$1.24\times 10^{102}$	$2.29\times 10^{202}$	$2.82\times 10^{202}$	$1.99\times 10^{3}$	$6.42\times 10^{602}$	$5.26\times 10^{502}$	$4.68\times 10^{402}$
	*p*-value	<$10^{-11}$	<$10^{-11}$	<$10^{-11}$	<$10^{-11}$	<$10^{-11}$	<$10^{-11}$	<$10^{-11}$	<$10^{-11}$	<$10^{-11}$
	Rank	4	11	6	7	8	17	13	15	12
F28	Mean	$5.25\times 10^{3}$	$1.48\times 10^{4}$	$6.30\times 10^{3}$	$9.40\times 10^{3}$	$1.63\times 10^{4}$	$7.91\times 10^{4}$	$9.07\times 10^{3}$	$3.05\times 10^{4}$	$1.30\times 10^{4}$
	Std	$6.11\times 10^{602}$	$4.89\times 10^{3}$	$3.13\times 10^{302}$	$9.31\times 10^{902}$	$2.29\times 10^{3}$	$7.83\times 10^{3}$	$1.75\times 10^{3}$	$3.92\times 10^{3}$	$1.87\times 10^{3}$
	*p*-value	<$10^{-11}$	<$10^{-11}$	<$10^{-11}$	0.0003	<$10^{-11}$	<$10^{-11}$	0.0166	<$10^{-11}$	<$10^{-11}$
	Rank	4	10	5	7	12	17	8	15	9
F29	Mean	$9.12\times 10^{3}$	$1.41\times 10^{4}$	$1.15\times 10^{4}$	$1.12\times 10^{4}$	$1.47\times 10^{4}$	$7.99\times 10^{4}$	$1.56\times 10^{4}$	$7.99\times 10^{4}$	$1.11\times 10^{4}$
	Std	$8.61\times 10^{802}$	$2.79\times 10^{3}$	$5.57\times 10^{502}$	$1.02\times 10^{3}$	$1.48\times 10^{3}$	$4.21\times 10^{4}$	$2.23\times 10^{3}$	$4.21\times 10^{4}$	$1.15\times 10^{3}$
	*p*-value	0.7189	<$10^{-11}$	<$10^{-11}$	<$10^{-11}$	<$10^{-11}$	<$10^{-11}$	<$10^{-11}$	<$10^{-11}$	<$10^{-11}$
	Rank	4	12	7	6	11	16	13	15	5
F30	Mean	$1.37\times 10^{8}$	$5.91\times 10^{8}$	$1.17\times 10^{9}$	$4.25\times 10^{8}$	$2.80\times 10^{9}$	$8.59\times 10^{10}$	$4.11\times 10^{8}$	$9.00\times 10^{9}$	$4.91\times 10^{8}$
	Std	$3.02\times 10^{8}$	$2.60\times 10^{8}$	$1.73\times 10^{8}$	$1.68\times 10^{8}$	$1.02\times 10^{9}$	$1.43\times 10^{10}$	$1.09\times 10^{8}$	$2.05\times 10^{9}$	$7.67\times 10^{8}$
	*p*-value	<$10^{-11}$	<$10^{-11}$	<$10^{-11}$	<$10^{-11}$	<$10^{-11}$	<$10^{-11}$	<$10^{-11}$	<$10^{-11}$	<$10^{-11}$
	Rank	8	12	15	10	16	17	9	18	11
+/≈/−		19/7/3	28/1/0	23/4/2	24/3/2	29/0/0	29/0/0	27/2/0	29/0/0	26/2/1
Mean rank		4.83	9.34	7.69	6.28	11.62	17.03	7.31	12.34	7.83
Final ranking		3	11	7	5	14	17	9	15	10

**Table A9:** Comparison of HALA and other algorithms on the CEC2022 benchmark suite (Dim = 10)—Part 1: HALA vs. ALA, LSHADE, LSHADE-SPACMA, AOOA, BAEO, BPBO, CCO, CEO.

Function	Metric	HALA	ALA	LSHADE	LSHADE- SPACMA	AOOA	BAEO	BPBO	CCO	CEO
F1	Mean	$3.02\times 10^{2}$	$3.00\times 10^{2}$	$2.37\times 10^{3}$	$2.72\times 10^{3}$	$1.15\times 10^{3}$	$2.62\times 10^{4}$	$3.51\times 10^{2}$	$3.01\times 10^{2}$	$3.02\times 10^{2}$
	Std	$3.60\times 10^{0}$	$2.61\times 10^{-1}$	$1.55\times 10^{3}$	$1.79\times 10^{3}$	$9.75\times 10^{2}$	$7.83\times 10^{3}$	$7.26\times 10^{1}$	$5.86\times 10^{0}$	$1.73\times 10^{0}$
	*p*-value	–	0.0032	<$10^{-11}$	<$10^{-11}$	<$10^{-11}$	<$10^{-11}$	<$10^{-11}$	0.0006	0.1064
	Rank	3	1	13	14	10	17	9	2	3
F2	Mean	$4.04\times 10^{2}$	$4.09\times 10^{2}$	$4.15\times 10^{2}$	$4.14\times 10^{2}$	$4.30\times 10^{2}$	$3.64\times 10^{3}$	$4.07\times 10^{2}$	$4.27\times 10^{2}$	$4.15\times 10^{2}$
	Std	$4.01\times 10^{0}$	$1.29\times 10^{1}$	$1.80\times 10^{1}$	$1.37\times 10^{1}$	$2.87\times 10^{1}$	$1.78\times 10^{3}$	$2.27\times 10^{0}$	$3.09\times 10^{1}$	$2.61\times 10^{1}$
	*p*-value	–	0.0098	0.0007	0.0002	<$10^{-11}$	<$10^{-11}$	0.0008	0.0002	0.0157
	Rank	1	3	10	9	14	17	2	13	10
F3	Mean	$6.00\times 10^{2}$	$6.00\times 10^{2}$	$6.00\times 10^{2}$	$6.00\times 10^{2}$	$6.21\times 10^{2}$	$6.70\times 10^{2}$	$6.05\times 10^{2}$	$6.25\times 10^{2}$	$6.45\times 10^{2}$
	Std	$2.98\times 10^{-1}$	$6.26\times 10^{-2}$	$3.65\times 10^{-1}$	$4.99\times 10^{-1}$	$1.04\times 10^{1}$	$8.37\times 10^{0}$	$1.96\times 10^{0}$	$1.10\times 10^{1}$	$1.30\times 10^{1}$
	*p*-value	–	0.035	0.1359	0.3493	<$10^{-11}$	<$10^{-11}$	<$10^{-11}$	<$10^{-11}$	<$10^{-11}$
	Rank	1	1	1	1	13	18	6	14	17
F4	Mean	$8.15\times 10^{2}$	$8.19\times 10^{2}$	$8.10\times 10^{2}$	$8.08\times 10^{2}$	$8.35\times 10^{2}$	$8.93\times 10^{2}$	$8.24\times 10^{2}$	$8.44\times 10^{2}$	$8.41\times 10^{2}$
	Std	$5.45\times 10^{0}$	$8.45\times 10^{0}$	$2.59\times 10^{0}$	$3.08\times 10^{0}$	$1.33\times 10^{1}$	$1.06\times 10^{1}$	$8.18\times 10^{0}$	$1.87\times 10^{1}$	$9.01\times 10^{0}$
	*p*-value	–	0.0157	0.0007	0.0002	<$10^{-11}$	<$10^{-11}$	<$10^{-11}$	<$10^{-11}$	<$10^{-11}$
	Rank	5	7	3	1	12	18	9	15	13
F5	Mean	$9.04\times 10^{2}$	$9.01\times 10^{2}$	$9.01\times 10^{2}$	$9.02\times 10^{2}$	$1.13\times 10^{3}$	$2.26\times 10^{3}$	$9.21\times 10^{2}$	$1.44\times 10^{3}$	$1.46\times 10^{3}$
	Std	$4.27\times 10^{0}$	$6.93\times 10^{-1}$	$1.75\times 10^{0}$	$4.86\times 10^{0}$	$1.92\times 10^{2}$	$2.39\times 10^{2}$	$2.01\times 10^{1}$	$3.02\times 10^{2}$	$1.76\times 10^{2}$
	*p*-value	–	0.001	0.0002	0.0047	<$10^{-11}$	<$10^{-11}$	<$10^{-11}$	<$10^{-11}$	<$10^{-11}$
	Rank	4	1	1	3	13	17	8	15	16
F6	Mean	$1.89\times 10^{3}$	$2.36\times 10^{3}$	$2.05\times 10^{3}$	$2.29\times 10^{3}$	$4.71\times 10^{3}$	$1.56\times 10^{9}$	$2.34\times 10^{3}$	$4.59\times 10^{3}$	$2.39\times 10^{3}$
	Std	$3.42\times 10^{1}$	$1.42\times 10^{3}$	$2.98\times 10^{2}$	$6.80\times 10^{2}$	$2.23\times 10^{3}$	$1.04\times 10^{9}$	$8.70\times 10^{2}$	$2.47\times 10^{3}$	$6.52\times 10^{2}$
	*p*-value	–	0.023	<$10^{-11}$	0.0001	<$10^{-11}$	<$10^{-11}$	0.0001	<$10^{-11}$	<$10^{-11}$
	Rank	1	7	3	6	13	18	5	12	8
F7	Mean	$2.02\times 10^{3}$	$2.02\times 10^{3}$	$2.01\times 10^{3}$	$2.01\times 10^{3}$	$2.04\times 10^{3}$	$2.17\times 10^{3}$	$2.03\times 10^{3}$	$2.06\times 10^{3}$	$2.11\times 10^{3}$
	Std	$8.16\times 10^{0}$	$8.15\times 10^{0}$	$9.02\times 10^{0}$	$6.86\times 10^{0}$	$1.77\times 10^{1}$	$3.32\times 10^{1}$	$9.23\times 10^{0}$	$2.50\times 10^{1}$	$5.32\times 10^{1}$
	*p*-value	–	0.0016	0.0571	0.0004	<$10^{-11}$	<$10^{-11}$	<$10^{-11}$	<$10^{-11}$	<$10^{-11}$
	Rank	8	8	1	1	11	18	10	15	16
F8	Mean	$2.21\times 10^{3}$	$2.22\times 10^{3}$	$2.22\times 10^{3}$	$2.22\times 10^{3}$	$2.23\times 10^{3}$	$1.01\times 10^{4}$	$2.22\times 10^{3}$	$2.23\times 10^{3}$	$2.26\times 10^{3}$
	Std	$8.26\times 10^{0}$	$6.90\times 10^{0}$	$6.45\times 10^{0}$	$5.88\times 10^{0}$	$4.65\times 10^{0}$	$2.78\times 10^{4}$	$4.38\times 10^{0}$	$7.70\times 10^{0}$	$2.09\times 10^{1}$
	*p*-value	–	0.0157	0.0196	0.0111	<$10^{-11}$	<$10^{-11}$	<$10^{-11}$	<$10^{-11}$	<$10^{-11}$
	Rank	1	3	3	3	9	18	3	9	16
F9	Mean	$2.53\times 10^{3}$	$2.53\times 10^{3}$	$2.55\times 10^{3}$	$2.55\times 10^{3}$	$2.56\times 10^{3}$	$3.13\times 10^{3}$	$2.53\times 10^{3}$	$2.53\times 10^{3}$	$2.55\times 10^{3}$
	Std	$1.46\times 10^{-13}$	$5.75\times 10^{-1}$	$1.96\times 10^{1}$	$1.89\times 10^{1}$	$3.66\times 10^{1}$	$2.02\times 10^{2}$	$1.51\times 10^{-1}$	$1.28\times 10^{1}$	$5.08\times 10^{1}$
	*p*-value	–	0.3173	<$10^{-11}$	<$10^{-11}$	<$10^{-11}$	<$10^{-11}$	<$10^{-11}$	<$10^{-11}$	<$10^{-11}$
	Rank	1	1	11	11	15	18	1	1	11
F10	Mean	$2.52\times 10^{3}$	$2.54\times 10^{3}$	$2.53\times 10^{3}$	$2.52\times 10^{3}$	$2.50\times 10^{3}$	$3.40\times 10^{3}$	$2.50\times 10^{3}$	$2.50\times 10^{3}$	$2.79\times 10^{3}$
	Std	$4.27\times 10^{1}$	$1.06\times 10^{2}$	$4.64\times 10^{1}$	$3.74\times 10^{1}$	$3.69\times 10^{-1}$	$4.67\times 10^{2}$	$2.34\times 10^{-1}$	$7.14\times 10^{-1}$	$3.39\times 10^{2}$
	*p*-value	–	0.9099	0.6733	0.959	0.0571	<$10^{-11}$	0.0897	0.0859	<$10^{-11}$
	Rank	7	11	9	7	1	17	1	1	16
F11	Mean	$2.67\times 10^{3}$	$2.66\times 10^{3}$	$2.74\times 10^{3}$	$2.76\times 10^{3}$	$2.77\times 10^{3}$	$4.75\times 10^{3}$	$2.66\times 10^{3}$	$2.81\times 10^{3}$	$2.85\times 10^{3}$
	Std	$1.13\times 10^{2}$	$1.57\times 10^{2}$	$3.67\times 10^{1}$	$8.35\times 10^{1}$	$1.33\times 10^{2}$	$5.70\times 10^{2}$	$6.61\times 10^{1}$	$1.73\times 10^{2}$	$1.22\times 10^{2}$
	*p*-value	–	0.147	0.0087	0.0009	0.0007	<$10^{-11}$	0.2369	0.0016	<$10^{-11}$
	Rank	4	2	6	8	9	18	2	11	16
F12	Mean	$2.86\times 10^{3}$	$2.86\times 10^{3}$	$2.87\times 10^{3}$	$2.87\times 10^{3}$	$2.87\times 10^{3}$	$3.22\times 10^{3}$	$2.86\times 10^{3}$	$2.87\times 10^{3}$	$2.92\times 10^{3}$
	Std	$1.50\times 10^{0}$	$1.54\times 10^{0}$	$2.53\times 10^{0}$	$2.54\times 10^{0}$	$5.04\times 10^{0}$	$1.56\times 10^{2}$	$1.38\times 10^{0}$	$2.32\times 10^{0}$	$5.74\times 10^{1}$
	*p*-value	–	0.0855	0.0001	0.0005	<$10^{-11}$	<$10^{-11}$	0.0047	<$10^{-11}$	<$10^{-11}$
	Rank	1	1	6	6	6	18	1	6	16
+/≈/−		–	5/4/3	7/3/2	7/2/3	11/1/0	12/0/0	10/2/0	10/1/1	11/1/0
Mean rank (global)		2.58	3.17	5.08	5.08	9.92	17.75	4.00	9.25	12.83
Final ranking (global)		1	2	5	5	14	18	3	11	15

**Table A10:** Comparison of HALA and other algorithms on the CEC2022 benchmark suite (Dim = 10)—Part 2: HALA vs. CQALA, DFL, DMOA, DHOA, FGO, KLA, PGA, SO, SOO (continued from Table A9).

Function	Metric	CQALA	DFL	DMOA	DHOA	FGO	KLA	PGA	SO	SOO
F1	Mean	$3.05\times 10^{2}$	$3.17\times 10^{2}$	$1.59\times 10^{3}$	$4.41\times 10^{2}$	$1.85\times 10^{3}$	$6.94\times 10^{4}$	$3.05\times 10^{2}$	$1.99\times 10^{4}$	$3.44\times 10^{3}$
	Std	$1.01\times 10^{1}$	$3.49\times 10^{1}$	$5.53\times 10^{2}$	$7.95\times 10^{1}$	$6.35\times 10^{2}$	$6.18\times 10^{4}$	$4.93\times 10^{0}$	$5.27\times 10^{3}$	$3.04\times 10^{3}$
	*p*-value	0.382	0.0157	<$10^{-11}$	<$10^{-11}$	<$10^{-11}$	<$10^{-11}$	0.0001	<$10^{-11}$	<$10^{-11}$
	Rank	5	8	12	10	15	18	5	16	17
F2	Mean	$4.08\times 10^{2}$	$4.17\times 10^{2}$	$4.22\times 10^{2}$	$4.14\times 10^{2}$	$4.23\times 10^{2}$	$3.70\times 10^{3}$	$4.33\times 10^{2}$	$4.82\times 10^{2}$	$4.17\times 10^{2}$
	Std	$1.33\times 10^{1}$	$2.42\times 10^{1}$	$1.48\times 10^{1}$	$2.01\times 10^{1}$	$1.88\times 10^{1}$	$1.64\times 10^{3}$	$3.74\times 10^{1}$	$2.90\times 10^{1}$	$2.59\times 10^{1}$
	*p*-value	0.0081	0.0002	<$10^{-11}$	0.0185	<$10^{-11}$	<$10^{-11}$	0.0004	<$10^{-11}$	0.0001
	Rank	4	11	15	8	16	18	17	18	11
F3	Mean	$6.15\times 10^{2}$	$6.08\times 10^{2}$	$6.08\times 10^{2}$	$6.04\times 10^{2}$	$6.12\times 10^{2}$	$6.42\times 10^{2}$	$6.11\times 10^{2}$	$6.34\times 10^{2}$	$6.09\times 10^{2}$
	Std	$8.38\times 10^{0}$	$7.79\times 10^{0}$	$1.42\times 10^{0}$	$1.97\times 10^{0}$	$8.86\times 10^{0}$	$8.12\times 10^{0}$	$6.00\times 10^{0}$	$1.02\times 10^{1}$	$5.96\times 10^{0}$
	*p*-value	<$10^{-11}$	<$10^{-11}$	<$10^{-11}$	<$10^{-11}$	<$10^{-11}$	<$10^{-11}$	<$10^{-11}$	<$10^{-11}$	<$10^{-11}$
	Rank	12	7	7	5	11	16	10	15	9
F4	Mean	$8.21\times 10^{2}$	$8.34\times 10^{2}$	$8.35\times 10^{2}$	$8.10\times 10^{2}$	$8.42\times 10^{2}$	$8.52\times 10^{2}$	$8.24\times 10^{2}$	$8.65\times 10^{2}$	$8.20\times 10^{2}$
	Std	$5.17\times 10^{0}$	$1.23\times 10^{1}$	$4.94\times 10^{0}$	$3.38\times 10^{0}$	$8.21\times 10^{0}$	$8.94\times 10^{0}$	$7.17\times 10^{0}$	$1.23\times 10^{1}$	$1.05\times 10^{1}$
	*p*-value	0.0001	<$10^{-11}$	<$10^{-11}$	0.0002	<$10^{-11}$	<$10^{-11}$	0.0001	<$10^{-11}$	0.0428
	Rank	6	10	11	3	13	17	8	16	5
F5	Mean	$1.05\times 10^{3}$	$9.84\times 10^{2}$	$9.30\times 10^{2}$	$9.07\times 10^{2}$	$9.91\times 10^{2}$	$3.11\times 10^{3}$	$9.10\times 10^{2}$	$1.79\times 10^{3}$	$9.67\times 10^{2}$
	Std	$9.61\times 10^{1}$	$1.42\times 10^{2}$	$6.89\times 10^{0}$	$7.21\times 10^{0}$	$4.49\times 10^{1}$	$9.36\times 10^{2}$	$2.26\times 10^{1}$	$3.61\times 10^{2}$	$6.17\times 10^{1}$
	*p*-value	<$10^{-11}$	0.0001	<$10^{-11}$	0.0157	<$10^{-11}$	<$10^{-11}$	0.2369	<$10^{-11}$	<$10^{-11}$
	Rank	12	10	9	6	11	18	7	16	13
F6	Mean	$2.10\times 10^{3}$	$5.66\times 10^{3}$	$4.17\times 10^{5}$	$2.60\times 10^{3}$	$1.03\times 10^{6}$	$1.27\times 10^{9}$	$4.21\times 10^{3}$	$2.64\times 10^{6}$	$3.51\times 10^{3}$
	Std	$3.31\times 10^{2}$	$2.11\times 10^{3}$	$3.17\times 10^{5}$	$1.34\times 10^{3}$	$1.57\times 10^{6}$	$8.99\times 10^{8}$	$1.82\times 10^{3}$	$2.12\times 10^{6}$	$1.67\times 10^{3}$
	*p*-value	0.0001	<$10^{-11}$	<$10^{-11}$	<$10^{-11}$	<$10^{-11}$	<$10^{-11}$	<$10^{-11}$	<$10^{-11}$	<$10^{-11}$
	Rank	2	11	15	9	16	17	10	14	13
F7	Mean	$2.05\times 10^{3}$	$2.04\times 10^{3}$	$2.05\times 10^{3}$	$2.03\times 10^{3}$	$2.05\times 10^{3}$	$2.10\times 10^{3}$	$2.04\times 10^{3}$	$2.09\times 10^{3}$	$2.04\times 10^{3}$
	Std	$5.09\times 10^{1}$	$3.50\times 10^{1}$	$5.96\times 10^{0}$	$1.03\times 10^{1}$	$1.43\times 10^{1}$	$5.04\times 10^{1}$	$1.79\times 10^{1}$	$1.88\times 10^{1}$	$2.03\times 10^{1}$
	*p*-value	0.0001	<$10^{-11}$	<$10^{-11}$	0.0002	<$10^{-11}$	<$10^{-11}$	<$10^{-11}$	<$10^{-11}$	<$10^{-11}$
	Rank	11	8	11	6	11	16	8	14	8
F8	Mean	$2.22\times 10^{3}$	$2.24\times 10^{3}$	$2.23\times 10^{3}$	$2.22\times 10^{3}$	$2.23\times 10^{3}$	$3.75\times 10^{3}$	$2.23\times 10^{3}$	$2.24\times 10^{3}$	$2.22\times 10^{3}$
	Std	$8.33\times 10^{0}$	$3.57\times 10^{1}$	$2.13\times 10^{0}$	$4.47\times 10^{0}$	$6.00\times 10^{0}$	$7.94\times 10^{3}$	$2.37\times 10^{1}$	$7.78\times 10^{0}$	$5.34\times 10^{0}$
	*p*-value	<$10^{-11}$	<$10^{-11}$	<$10^{-11}$	<$10^{-11}$	<$10^{-11}$	<$10^{-11}$	<$10^{-11}$	<$10^{-11}$	<$10^{-11}$
	Rank	3	13	9	3	9	17	9	13	3
F9	Mean	$2.54\times 10^{3}$	$2.53\times 10^{3}$	$2.55\times 10^{3}$	$2.53\times 10^{3}$	$2.54\times 10^{3}$	$3.04\times 10^{3}$	$2.54\times 10^{3}$	$2.56\times 10^{3}$	$2.53\times 10^{3}$
	Std	$3.73\times 10^{1}$	$1.47\times 10^{1}$	$7.48\times 10^{0}$	$9.69\times 10^{-2}$	$3.19\times 10^{1}$	$1.99\times 10^{2}$	$3.65\times 10^{1}$	$2.26\times 10^{1}$	$3.66\times 10^{0}$
	*p*-value	0.0679	<$10^{-11}$	<$10^{-11}$	<$10^{-11}$	<$10^{-11}$	<$10^{-11}$	<$10^{-11}$	<$10^{-11}$	<$10^{-11}$
	Rank	8	5	11	1	8	17	8	15	1
F10	Mean	$2.61\times 10^{3}$	$2.70\times 10^{3}$	$2.50\times 10^{3}$	$2.52\times 10^{3}$	$2.50\times 10^{3}$	$3.38\times 10^{3}$	$2.56\times 10^{3}$	$2.50\times 10^{3}$	$2.59\times 10^{3}$
	Std	$3.68\times 10^{1}$	$2.82\times 10^{2}$	$3.04\times 10^{-1}$	$4.44\times 10^{1}$	$5.34\times 10^{-1}$	$6.62\times 10^{2}$	$6.51\times 10^{1}$	$9.77\times 10^{-1}$	$2.05\times 10^{2}$
	*p*-value	<$10^{-11}$	0.0001	0.0859	0.1306	0.0571	<$10^{-11}$	0.0003	0.0571	0.0053
	Rank	14	15	1	8	1	16	13	1	12
F11	Mean	$2.76\times 10^{3}$	$2.83\times 10^{3}$	$2.73\times 10^{3}$	$2.65\times 10^{3}$	$2.80\times 10^{3}$	$4.72\times 10^{3}$	$2.83\times 10^{3}$	$2.84\times 10^{3}$	$2.79\times 10^{3}$
	Std	$1.56\times 10^{2}$	$1.88\times 10^{2}$	$1.40\times 10^{1}$	$7.63\times 10^{1}$	$1.28\times 10^{2}$	$6.07\times 10^{2}$	$1.95\times 10^{2}$	$1.64\times 10^{2}$	$1.49\times 10^{2}$
	*p*-value	0.0598	0.0001	0.0148	0.7655	0.0005	<$10^{-11}$	0.0001	0.0001	0.0001
	Rank	8	14	7	1	10	17	14	15	9
F12	Mean	$2.88\times 10^{3}$	$2.87\times 10^{3}$	$2.87\times 10^{3}$	$2.87\times 10^{3}$	$2.86\times 10^{3}$	$2.95\times 10^{3}$	$2.87\times 10^{3}$	$2.87\times 10^{3}$	$2.87\times 10^{3}$
	Std	$2.70\times 10^{1}$	$1.96\times 10^{1}$	$4.60\times 10^{-1}$	$1.41\times 10^{0}$	$1.38\times 10^{0}$	$6.60\times 10^{1}$	$1.90\times 10^{0}$	$1.60\times 10^{0}$	$6.65\times 10^{0}$
	*p*-value	<$10^{-11}$	<$10^{-11}$	<$10^{-11}$	<$10^{-11}$	0.0015	<$10^{-11}$	<$10^{-11}$	<$10^{-11}$	<$10^{-11}$
	Rank	15	10	6	10	1	17	10	10	10
+/≈/−		9/3/0	12/0/0	11/1/0	9/2/1	11/1/0	12/0/0	11/1/0	11/1/0	12/0/0
Mean rank		8.25	9.25	8.67	4.58	9.67	17.00	9.00	13.42	7.92
Final ranking		8	11	9	4	13	17	10	16	7

## References

[1] Pan J.S., Hu P., Snášel V., Chu S.C. (2023). A survey on binary metaheuristic algorithms and their engineering applications. Artif. Intell. Rev..

[2] Ouyang H., Lin X., Li S., Gao L., Houssein E.H. (2025). Recent metaheuristic algorithms for multi-objective feature selection: Review, applications, open issues and challenges. Clust. Comput..

[3] Chen Q. (2026). Financial cost optimization of urban water resource scheduling using genetic algorithms: A metaheuristic approach. Expert Syst. Appl..

[4] Peng Y., Gu S., Liang Y., Ouyang K., Li Y., Wang K., Wu G., Fan C. (2025). Wave Optics Optimizer: A novel meta-heuristic algorithm for engineering optimization. Commun. Nonlinear Sci. Numer. Simul..

[5] Yadav S., Ravi V., Kalyanakrishnan S. (2025). Hybrids of Reinforcement Learning and Evolutionary Computation in Finance: A Survey. ACM Comput. Surv..

[6] Zhang R., Wang J., Liu C., Su K., Ishibuchi H., Jin Y. (2025). Synergistic integration of metaheuristics and machine learning: Latest advances and emerging trends. Artif. Intell. Rev..

[7] Rajwar K., Deep K., Das S. (2023). An exhaustive review of the metaheuristic algorithms for search and optimization: Taxonomy, applications, and open challenges. Artif. Intell. Rev..

[8] Yang L., Xia Y., Ye L., Gao R., Zhan Y. (2023). A fully hybrid algorithm for deadline constrained workflow scheduling in clouds. IEEE Trans. Cloud Comput..

[9] Li Y., He Y., Lin J., Xu Z., Zhang S. (2025). A reinforcement learning-based population hyper-heuristic for energy-efficient cloud workflow scheduling problem. IEEE Trans. Serv. Comput..

[10] Ye L., Yang L., Xia Y., Zhao X. (2024). A Cost-Driven Intelligence Scheduling Approach for Deadline-Constrained IoT Workflow Applications in Cloud Computing. IEEE Internet Things J..

[11] Daoud M.S., Shehab M., Al-Mimi H.M., Abualigah L., Zitar R.A., Shambour M.K.Y. (2023). Gradient-based optimizer (GBO): A review, theory, variants, and applications. Arch. Comput. Methods Eng..

[12] Hussain K., Mohd Salleh M.N., Cheng S., Shi Y. (2019). Metaheuristic research: A comprehensive survey. Artif. Intell. Rev..

[13] Abualigah L., Elaziz M.A., Khasawneh A.M., Alshinwan M., Ibrahim R.A., Al-Qaness M.A., Mirjalili S., Sumari P., Gandomi A.H. (2022). Meta-heuristic optimization algorithms for solving real-world mechanical engineering design problems: A comprehensive survey, applications, comparative analysis, and results. Neural Comput. Appl..

[14] Xiao Y., Cui H., Khurma R.A., Castillo P.A. (2025). Artificial lemming algorithm: A novel bionic meta-heuristic technique for solving real-world engineering optimization problems. Artif. Intell. Rev..

[15] Wolpert D.H., Macready W.G. (1997). No free lunch theorems for optimization. IEEE Trans. Evol. Comput..

[16] Yang K., Zheng J., Zou J., Yu F., Yang S. (2023). A dual-population evolutionary algorithm based on adaptive constraint strength for constrained multi-objective optimization. Swarm Evol. Comput..

[17] Chen S., Wan H., Peng B., Quan R., Chang Y., Derigent W. (2026). Accurate multi-step wind and solar power forecasting based on multi-scale convolutional Kolmogorov-Arnold network and improved Lemming-optimized attention fusion. Eng. Appl. Artif. Intell..

[18] Xie Y., Sun Z., Yuan K., Sun Z. (2025). 3D UAV Route Optimization in Complex Environments Using an Enhanced Artificial Lemming Algorithm. Symmetry.

[19] Zhu X., Jia C., Zhao J., Xia C., Peng W., Huang J., Li L. (2025). An Enhanced Artificial Lemming Algorithm and Its Application in UAV Path Planning. Biomimetics.

[20] Tanabe R., Fukunaga A. Success-history based parameter adaptation for Differential Evolution. Proceedings of the 2013 IEEE Congress on Evolutionary Computation.

[21] Tanabe R., Fukunaga A.S. Improving the search performance of SHADE using linear population size reduction. Proceedings of the 2014 IEEE Congress on Evolutionary Computation (CEC).

[22] Mohamed A.W., Hadi A.A., Fattouh A.M., Jambi K.M. LSHADE with semi-parameter adaptation hybrid with CMA-ES for solving CEC 2017 benchmark problems. Proceedings of the 2017 IEEE Congress on Evolutionary Computation (CEC).

[23] Brest J., Maučec M.S., Bošković B. (2017). Single objective real-parameter optimization: Algorithm jSO. Proceedings of the 2017 IEEE Congress on Evolutionary Computation (CEC), Donostia, Spain, 5–8 June 2017.

[24] Omidvar M.N., Li X., Mei Y., Yao X. (2014). Cooperative co-evolution with differential grouping for large scale optimization. IEEE Trans. Evol. Comput..

[25] Mallipeddi R., Suganthan P.N., Pan Q., Tasgetiren M.F. (2011). Differential evolution algorithm with ensemble of parameters and mutation strategies. Appl. Soft Comput..

[26] Wang Y., Cai Z., Zhang Q. (2011). Differential evolution with composite trial vector generation strategies and control parameters. IEEE Trans. Evol. Comput..

[27] Wu G., Mallipeddi R., Suganthan P.N., Wang R., Chen H. (2016). Differential evolution with multi-population based ensemble of mutation strategies. Inf. Sci..

[28] Brest J., Greiner S., Bošković B., Mernik M., Žumer V. (2006). Self-adapting control parameters in differential evolution: A comparative study on numerical benchmark problems. IEEE Trans. Evol. Comput..

[29] Erlich I., Venayagamoorthy G.K., Worawat N. (2010). A mean-variance optimization algorithm. Proceedings of the 2010 IEEE World Congress on Computational Intelligence (WCCI), Barcelona, Spain, 18–23 July 2010.

[30] Hansen N. (2009). Benchmarking a BI-population CMA-ES on the BBOB-2009 function testbed. Proceedings of the 11th Annual Conference Companion on Genetic and Evolutionary Computation Conference: Late Breaking Papers.

[31] Zhang J., Sanderson A.C. (2009). JADE: Adaptive differential evolution with optional external archive. IEEE Trans. Evol. Comput..

[32] Wu G., Mallipeddi R., Suganthan P.N. (2017). Problem Definitions and Evaluation Criteria for the CEC 2017 Competition on Constrained Real-Parameter Optimization.

[33] Biedrzycki R. (2025). Analysis and Simplification of the Winner of the CEC 2022 Optimization Competition on Single Objective Bound Constrained Search. Evol. Comput..

[34] Derrac J., García S., Molina D., Herrera F. (2011). A practical tutorial on the use of nonparametric statistical tests as a methodology for comparing evolutionary and swarm intelligence algorithms. Swarm Evol. Comput..

[35] Saheed Y.K., Balogun B.F., Odunayo B.J., Abdulsalam M. (2023). Microarray Gene Expression Data Classification via Wilcoxon Sign Rank Sum and Novel Grey Wolf Optimized Ensemble Learning Models. IEEE/ACM Trans. Comput. Biol. Bioinform..

[36] Theodorsson-Norheim E. (1987). Friedman and Quade tests: BASIC computer program to perform nonparametric two-way analysis of variance and multiple comparisons on ranks of several related samples. Comput. Biol. Med..

[37] Wang R.B., Hu R.B., Geng F.D., Xu L., Chu S.C., Pan J.S., Meng Z.Y., Mirjalili S. (2025). The Animated Oat Optimization Algorithm: A nature-inspired metaheuristic for engineering optimization and a case study on Wireless Sensor Networks. Knowl.-Based Syst..

[38] Zhao S., Meng F., Cai L., Yang R. (2025). Boomerang aerodynamic ellipse optimizer: A human game-inspired optimization technique for numerical optimization and multilevel thresholding image segmentation. Math. Comput. Simul..

[39] Ghasemi M., Akbari M.A., Zare M., Mirjalili S., Deriche M., Abualigah L., Khodadadi N. (2025). Birds of prey-based optimization (BPBO): A metaheuristic algorithm for optimization. Evol. Intell..

[40] Wang T.L., Gu S.W., Liu R.J., Chen L.Q., Wang Z., Zeng Z.Q. (2025). Cuckoo catfish optimizer: A new meta-heuristic optimization algorithm. Artif. Intell. Rev..

[41] Dong Y., Zhang S., Zhang H., Zhou X., Jiang J. (2025). Chaotic evolution optimization: A novel metaheuristic algorithm inspired by chaotic dynamics. Chaos Solitons Fractals.

[42] Çelik E., Houssein E.H., Abdel-Salam M., Oliva D., Tejani G.G., Öztürk N., Sharma S.K., Baljon M. (2025). Novel distance-fitness learning scheme for ameliorating metaheuristic optimization. Eng. Sci. Technol. Int. J..

[43] Lang Y., Gao Y. (2025). Dream Optimization Algorithm (DOA): A novel metaheuristic optimization algorithm inspired by human dreams and its applications to real-world engineering problems. Comput. Methods Appl. Mech. Eng..

[44] Mohammed B.O., Aghdasi H.S., Salehpour P. (2025). Dhole optimization algorithm: A new metaheuristic algorithm for solving optimization problems. Clust. Comput..

[45] Abdel-Basset M., Mohamed R., Abouhawwash M. (2025). Fungal growth optimizer: A novel nature-inspired metaheuristic algorithm for stochastic optimization. Comput. Methods Appl. Mech. Eng..

[46] Ghasemi M., Khodadadi N., Trojovskỳ P., Li L., Mansor Z., Abualigah L., Alharbi A.H., El-Kenawy E.S.M. (2025). Kirchhoff’s law algorithm (KLA): A novel physics-inspired non-parametric metaheuristic algorithm for optimization problems. Artif. Intell. Rev..

[47] Bohat V.K., Hashim F.A., Batra H., Elaziz M.A. (2025). Phototropic growth algorithm: A novel metaheuristic inspired from phototropic growth of plants. Knowl.-Based Syst..

[48] Hussein N.K., Qaraad M., El Najjar A.M., Farag M., Elhosseini M.A., Mirjalili S., Guinovart D. (2025). Schrödinger optimizer: A quantum duality-driven metaheuristic for stochastic optimization and engineering challenges. Knowl.-Based Syst..

[49] Rodan A., Al-Tamimi A.K., Al-Alnemer L., Mirjalili S. (2025). Stellar oscillation optimizer: A nature-inspired metaheuristic optimization algorithm. Clust. Comput..

[50] Arcuri A., Fraser G. (2013). Parameter tuning or default values? An empirical investigation in search-based software engineering. Empir. Softw. Eng..

[51] Coello C.A.C. (2002). Theoretical and numerical constraint-handling techniques used with evolutionary algorithms: A survey of the state of the art. Comput. Methods Appl. Mech. Eng..

[52] Rajeswara Rao B., Tiwari R. (2007). Optimum design of rolling element bearings using genetic algorithms. Mech. Mach. Theory.

[53] Tzanetos A., Blondin M. (2023). A qualitative systematic review of metaheuristics applied to tension/compression spring design problem: Current situation, recommendations, and research direction. Eng. Appl. Artif. Intell..

[54] Chickermane H., Gea H.C. (1996). Structural optimization using a new local approximation method. Int. J. Numer. Methods Eng..

[55] Rao R., Savsani V., Vakharia D. (2011). Teaching–learning-based optimization: A novel method for constrained mechanical design optimization problems. Comput.-Aided Des..

